# Wide-scale geographical analysis of genetic ancestry in the South African Coloured population

**DOI:** 10.1186/s12915-025-02317-5

**Published:** 2025-07-22

**Authors:** Imke Lankheet, Rickard Hammarén, Lucía Ximena Alva Caballero, Maximilian Larena, Helena Malmström, Cecile Jolly, Himla Soodyall, Michael de Jongh, Carina Schlebusch

**Affiliations:** 1 Human Evolution, Department of Organismal Biology, Evolutionary Biology Centre, Uppsala University, Uppsala, Sweden; 2 Division of Human Genetics, School of Pathology, Faculty of Health Sciences, University of the Witwatersrand, Johannesburg, South Africa; 3 Academy of Science of South Africa, Pretoria, South Africa; 4 Department of Anthropology and Archaeology, University of South Africa, Pretoria, South Africa; 5 Palaeo-Research Institute, University of Johannesburg, Johannesburg, South Africa; 6 SciLife Lab, Uppsala, Sweden

**Keywords:** South African Coloured population, Genetic admixture, Khoe-San ancestry, Sex-biased admixture

## Abstract

**Background:**

The South African Coloured (SAC) population, a prominent admixed population in South Africa, reflects centuries of migration, admixture, and historical segregation. Descendants of local Khoe-San and Bantu-speaking populations, European settlers, and enslaved individuals from Africa and Asia, SAC individuals embody diverse ancestries. This study investigates the genetic makeup of SAC individuals, utilizing autosomal genotypes, mitochondrial DNA and Y-chromosome data. We analyse new genotype data for 125 SAC individuals from seven locations.

**Results:**

Our analysis, based on a dataset comprising 356 SAC individuals from 22 geographic locations, revealed significant regional variations in ancestry. Khoe-San ancestry predominates in 14 locations, highlighting its lasting influence. Inland regions exhibit higher proportions of Khoe-San ancestry, eastern regions show more Bantu-speaker/West African ancestry, and western/coastal areas, particularly around Cape Town, display increased Asian ancestry. Additionally, sex-biased admixture ratios show male-biased admixture from East Africans and Europeans, and female-biased admixture from Khoe-San populations, which is supported by mitochondrial and Y-chromosome data.

**Conclusions:**

The observed patterns of significant regional variation in ancestry reflect historical migrations and settlement patterns. This research underscores the importance of studying the SAC population to understand South Africa’s historical migrations, providing insights into the complex genetic heritage of South Africans.

**Supplementary Information:**

The online version contains supplementary material available at 10.1186/s12915-025-02317-5.

## Background

The South African Coloured (SAC) population is among the most admixed populations in the world, and SAC individuals trace their genetic roots to local Khoe-San and Bantu-speaking groups, European colonists, and enslaved people from other regions in Africa and from Asia. Genetically, Khoe-San populations represent one of the two branches of the earliest population divergence of the human population tree and therefore show high genetic diversity [1–3]. They also host early diverging mitochondrial and Y-chromosome lineages [4–7]. Until approximately 2000 years ago, the San ancestors were the only inhabitants of Southern Africa and they practised hunter-gathering [8]. Around 2000 years ago, East-African pastoralists arrived in Southern Africa, and admixed with the local San populations [1, 2, 9–13], giving rise to the Khoekhoe herding groups. Today, Khoe-San is the term used to refer to the hunter-gatherer San and the herder Khoekhoe collectively [14, 15]. The arrival of East-African pastoralists was followed by the arrival of Bantu-speaking groups practicing agriculture and carrying West African ancestry around 1800 years ago as part of the Bantu expansion [16–20]. The colonial times introduced both European and Asian ancestries into Southern Africa [21]. In 1652, the Dutch East India company founded a small refueling station that gradually grew over the decades into what became known as the Cape Colony and later on as Cape Town. The Dutch settlers interacted heavily with the local Khoekhoe communities from the very foundation of the colony. They traded for cattle and, as time went by, some Khoekhoe would work on settler farmsteads [22]. There were disproportionately few women among the settlers in the colony which led to formal and informal unions between European men and Khoekhoe women [22].

Over time, non-Europeans in the colony became less accepted, leading to the formation of a distinct community. Sometime after 1700, the term “Cape coloureds” emerged to refer to people of mixed ancestry [23]. The Cape coloureds were descendants of Khoe-San, Bantu-speaking populations, European settlers and enslaved people from the West and East Coast of Africa, the Indian subcontinent, Madagascar, and Indonesia, brought to South Africa during the slave trade period (1658–1806) [22]. The apartheid regime, the institutionalised racial segregation in place from 1948 to the early 1990 s, enhanced the unity of the South African Coloured group identity [22, 24].

Currently, the SAC population is the largest admixed population in the country [24]. They constitute more than half of the population of the Western Cape Province today, with large presences in the Northern and Eastern Cape provinces as well, see Fig. 2F. The majority of the SAC speak Afrikaans as their first language, 75.8% according to the 2011 South Africa (SA) census, and most SAC individuals are Christian. Religion differentiates SAC from the Cape Malay population, who practice Islam [23] and are also the result of admixture events between Africans and Asians [25]. Despite the term “Coloured” originating as a construct during the apartheid regime, its usage persists in contemporary South Africa, albeit with varied acceptance.

A number of studies has investigated the genetics of the SAC individuals [13, 26–29], mainly of those living close to Cape Town. They confirm the inferences drawn from historical records: the six main demographic groups that contributed to the genetic pool of the SAC were the Khoe-San (19.1–43.0%), Bantu-speakers/West Africans (17.9–33.0%), East Africans (< 3%), South Asians/Indians, Southeast Asians (Asian ancestries: 9.0–19.9%) and Europeans (19.3–38.5%). A mitochondrial DNA study revealed that the Khoe-San had a large maternal contribution to the SAC (60.0%), while the West Eurasian/European maternal contribution was very limited (4.6%) [27]. However, most of these studies focused on single locations and the majority of locations were close to Cape Town.

In this study, we combined newly generated genome-wide data from 125 individuals self-identifying as SAC with previously published genetic data from SAC individuals. The new dataset includes individuals from a broad range of geographic locations across the SAC community’s distribution, allowing, for the first time, an investigation into the geographical differences in ancestry contributions among the SAC. Based on written history and previous genetic studies, we can formalise several hypotheses about what to expect from the data we generated. We hypothesise that European ancestry would be highest in the western regions, particularly around Cape Town, due to the influx of European colonialists. Conversely, we expect Bantu-speaker ancestry to be more prevalent in the east, reflecting the historical expansion of Bantu-speaking groups as far south as the Fish River in the present-day Eastern Cape [9, 30, 31]. By integrating genome-wide data with uniparental markers, we also explored sex-biased admixture patterns. Given the historical records of formal and informal unions between predominantly male European migrants and Khoe-San women [22], we expect to observe a female-biased Khoe-San gene flow, and a male-biased European gene flow. This pattern was also observed in a previous genetic study on the SAC population from Wellington [13]. Our present analysis provides a more comprehensive view of the ancestral genetic components across widely distributed SAC populations, allowing us to describe geographic differences in ancestry contributions, investigate differences in maternal and paternal lineages using mitogenomes and Y chromosomes, and explore sex-biased admixture patterns through X-to-autosomal comparisons.

## Results

In this study, we aim to provide a comprehensive analysis of the genetic ancestry of the South African Coloured (SAC) population by investigating genome-wide data from 356 (125 new) individuals self-identifying as SAC, from 22 (7 new) locations in South Africa (Additional File 1: Fig. S1). Building upon previous genetic research, our investigation encompasses a thorough examination of ancestral genetic components within the SAC, for the first time focusing on geographically dispersed SAC groups. Employing a combination of genomic techniques, including analysis of mitogenomes, Y chromosomes, and X-to-autosomal comparisons, we investigate the complexities of admixture events and explore geographic variations in ancestral contributions. Through these approaches, we seek to elucidate the complex genetic make up of SAC and shed light on the historical and demographic factors that have shaped this diverse population.

### Autosomal ancestry contribution in geographically dispersed SAC groups

**Fig. 1:**
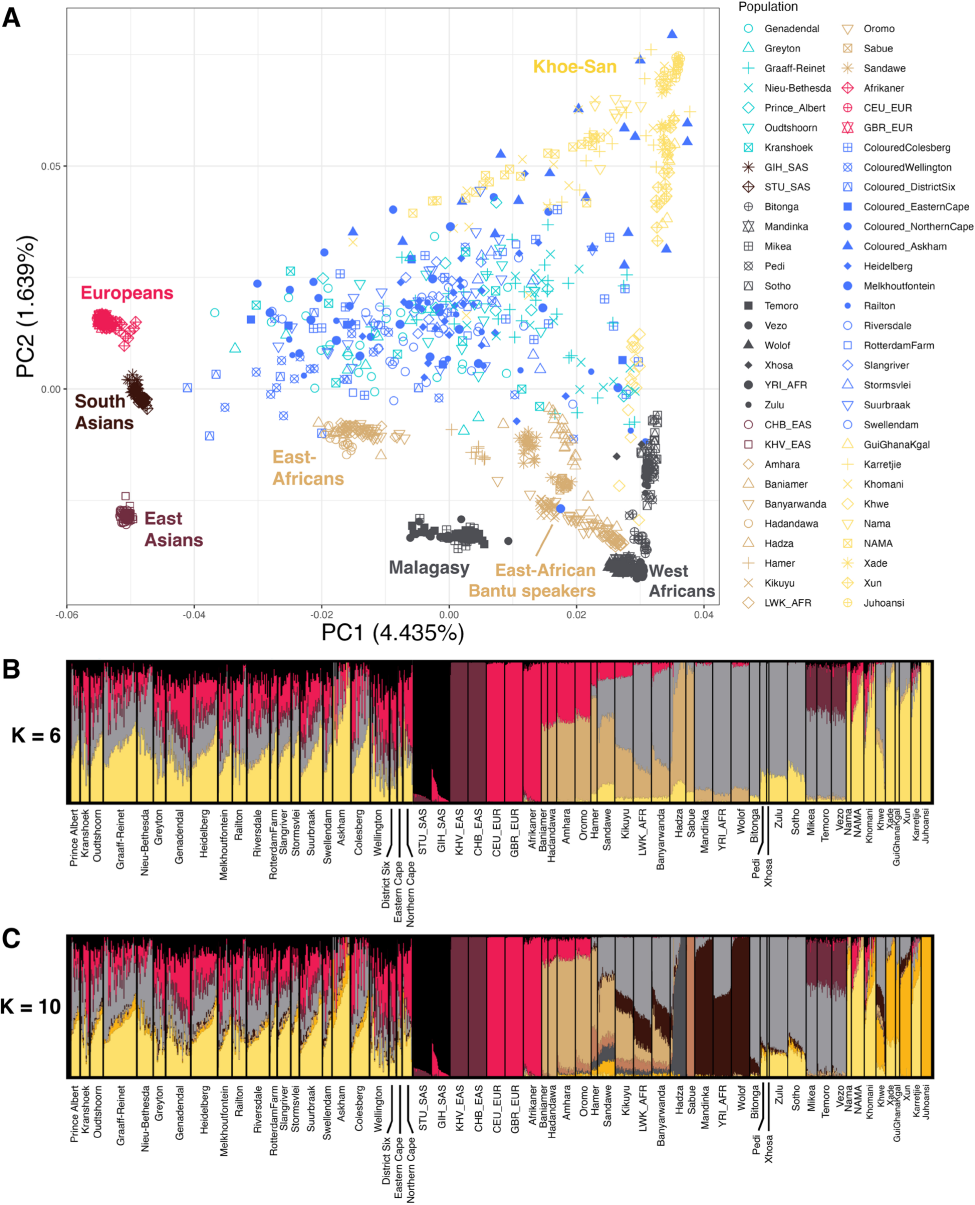
Population structure and genetic affinities of South African Coloured population. Principal component analysis (PCA) and ADMIXTURE results for the populations in our dataset, including 356 SAC individuals. In **A**, principal component analysis (PCA) results are shown, where PC1 and PC2 are plotted against each other. Labels according to continental groups were added *a posteriori* to help with legibility. The new SAC samples are shown in light blue, the previously published ones in dark blue. For geographical origins of populations, see Additional File 1: Fig. S1. Other PCA projections can be found in Additional File 2: Fig. S2. **B** and **C** show ADMIXTURE results, visualized using PONG for *K* = 6 and *K* = 10, respectively. ADMIXTURE results for *K *= 2 to *K *= 12 can be found in Additional File 6: Fig. S6. GIH_SAS are the Gujarati Indians, STU_SAS are the Sri Lankan Tamil, YRI_AFR are the Yoruba from Nigeria, CHB_EAS are the Han from China, KHV_EAS are the Kinh from Vietnam, LWK_AFR are the Luhya from Kenya, CEU_EUR are Utah residents with Northern and Western European ancestry, and the GBR_EUR are the British in England and Scotland

We created a database consisting of 356 SAC individuals and 847 reference individuals. To capture the major genetic variation between continental groups and to investigate the affiliations of SAC individuals in this genetic space, we applied principal component analysis (PCA) to our dataset. The first principal component separates the out-of-Africa populations from the Khoe-San and West-African populations, while the second principal component represents the variation between Khoe-San and West African-related ancestries (Fig. 1A). The SAC individuals are observed scattered in-between these extremes, with some individuals associating more with either Khoe-San, non-African or West African groups. Moreover, PC3 separates East Asian and European ancestry, with South Asians grouping between these two extremes (Additional File 2: Fig. S2). Certain SAC individuals are off-set towards the Asian extreme, suggesting increased ancestry contributions from Asians. Analysing the average PC values per population (Additional File 3: Fig. S3) reveals a noticeable west-to-east pattern in the PCA. The western locations District Six, Wellington, Genadendal, and Greyton tend to cluster nearer to European populations, while the eastern locations Graaff-Reinet and Nieu-Bethesda show closer proximity to Khoe-San and West-African/Bantu-speaking groups. The three other new locations, Kranshoek, Oudtshoorn, and Prince Albert, which are located geographically in between the previously mentioned groups, also occupy the space in the PCA plot between these groups. Among the three, Kranshoek is closest to Genadendal and Greyton in the PCA plot. *F*_st_ values were calculated for each pairwise population comparison and can be found in Additional File 4: Fig. S4. This figure reiterates the clusters observed in the PCA, where the groups that cluster together in the PCA have low pairwise *F*_st_ values, indicating genetic similarity. The SAC populations display low *F*_st_ values, forming a genetically close group in the *F*_st_ analyses, despite their extensive genetic heterogeneity in the ADMIXTURE and PCA results. A Mantel test was performed to assess the correlation between genetic differentiation (*F*_st_) and geographic distance among the 22 SAC populations. The analysis revealed a significant positive correlation (*r *= 0.5171, *p *= 0.0017, 9999 permutations), suggesting that geographically distant SAC groups exhibit greater genetic differentiation.

Uniform manifold approximation and projection for dimension reduction (UMAP) identifies the major variation in the data and reduces it to two dimensions, thus allowing a graphical overview of the variation [32]. Unlike PCA, UMAP aims to capture more of the global variation [32]. The UMAP analysis recapitulates the major continental ancestries within the dataset, with the more drifted out-of-Africa populations forming tightly clustered groups away from each other (Additional File 5: Fig. S5). Khoe-San, SAC, and Bantu-speaker related ancestry populations form a larger group in the centre of the UMAP. Most of the SAC are positioned close to the Khoe-San populations but are drawn towards either the European or Bantu-speaker related ancestry. Some SAC individuals cluster firmly with other populations, rather than with the other SAC individuals. Three individuals from District Six, Northern Cape, and Genadendal are closely associated with the European populations. Three other individuals are associated with South Asians, two from Wellington and one from District Six. Additionally, five SAC individuals from various locations group with the South African Bantu-speaking populations. It is not surprising to see these SAC individuals group with these respective groups. Khoe-San have admixture with Bantu-speakers and Europeans. Similarly, Bantu-speakers have admixture with Khoe-San. This makes these ancestries somewhat continuous and this continuity in ancestries naturally leads to some SAC individuals grouping with their parental/donor groups.

To further investigate the population structure and ancestral contributions to the SAC populations, we performed unsupervised ADMIXTURE analysis for *K* = 2 to *K* = 12 (Additional File 6: Fig. S6). At *K* = 6 (Fig. 1B), we identified components corresponding to major continental and regional groups: Khoe-San, European, West African/Bantu-speakers, East African, East Asian, and South Asian. Compared to *K* = 6, *K* = 10 revealed additional clusters: one associated with the East African Hadza, another associated with the East African Sabue, a cluster separating northern from southern Khoe-San populations, and a cluster separating Bantu-speakers from West African non-Bantu Niger-Congo speakers. *K* = 10 (Fig. 1C) had the lowest cross-validation error (Additional File 7: Fig. S7). Average admixture fractions at *K* = 6 and *K* = 10 are shown in Additional File 22: Table S1 and S2, respectively. From the 22 locations with SAC individuals, Khoe-San ancestry is predominant at 14 locations, including the new locations of Graaff-Reinet, Nieu-Bethesda, Kranshoek, Oudtshoorn and Prince Albert. Khoe-San ancestry ranges from 12.0% (District Six) to 69.0% (Askham) across all sites, averaging at 33.4%. Based on *K* = 10 ADMIXTURE results, we can conclude that this observed Khoe-San ancestry is mostly southern Khoe-San rather than northern Khoe-San (yellow vs gold respectively in Fig. 1C). This was confirmed by *f*4-statistics in the form *f*4 (Chimp, SAC, Ju/’hoansi, Karretjie) (Additional File 8: Fig. S8). European ancestry is predominant in seven locations, including Genadendal and Greyton. Generally, European ancestry ranges between 9.2% (Nieu-Bethesda) and 40.5% (Northern Cape) in the studied SAC populations, with an average of 21.7%. In Railton, West-African ancestry constituted the largest proportion (32.8%) while the West-African ancestry was lowest in Northern Cape (9.4%).

At *K* = 9, ADMIXTURE analysis separates the West African ancestral component into a cluster maximised in West African non-Bantu Niger-Congo speakers (dark-brown) and another in Bantu-speaking populations (light grey) (Additional File 6: Fig. S6). From this *K* and higher, the component found among the SAC is mostly related to Bantu-speaker ancestry rather than non-Bantu Niger-Congo speakers. In addition, to directly evaluate the genetic affinity of West African/Bantu-speaker ancestry found in SAC, we conducted the test *f*4 (Chimp, SAC, YRI_AFR, Zulu) (Additional File 9: Fig. S9). All SAC groups, except the Coloured from Askham exhibit greater genetic affinity to the Yorubans relative to the South African Bantu-speaking Zulu.

*F*4-statistics were computed to assess the genetic affinity of the Asian component in the SAC population (*f*4 (Chimp, SAC, CHB_EAS, GIH_SAS)), where CHB_EAS are the Han Chinese (East Asian) and GIH_SAS are the Gujarati Indians (South Asians) (Additional File 10: Fig. S10). Positive values for most SAC groups imply more genetic affinity with South Asians rather than East Asians. The ADMIXTURE results also highlight an additional interesting aspect about the ancestry of the studied SAC populations, namely the presence of a genetic component shared with the Malagasy populations. From *K* = 4 to *K* = 10, the genetics of the Malagasy populations (Mikea, Temoro, Vezo) can be explained as being comprised mainly of two clusters; a West African cluster ($\sim$60%), and a East Asian cluster ($\sim$40%). However, from *K* = 11, the Malagasy populations get their own cluster (royal blue), with minor West African and East Asian contributions. This cluster can also be observed in the various SAC populations, at low percentages (average across all the studied SAC locations is 5.8%). Positive values for *f*4-statistics in the form *f*4 (Chimp, SAC, Malagasy, South Asian) (Additional File 11: Fig. S11) for all SAC groups imply they possess greater genetic affinity with South Asians relative to Malagasy people.

**Fig. 2:**
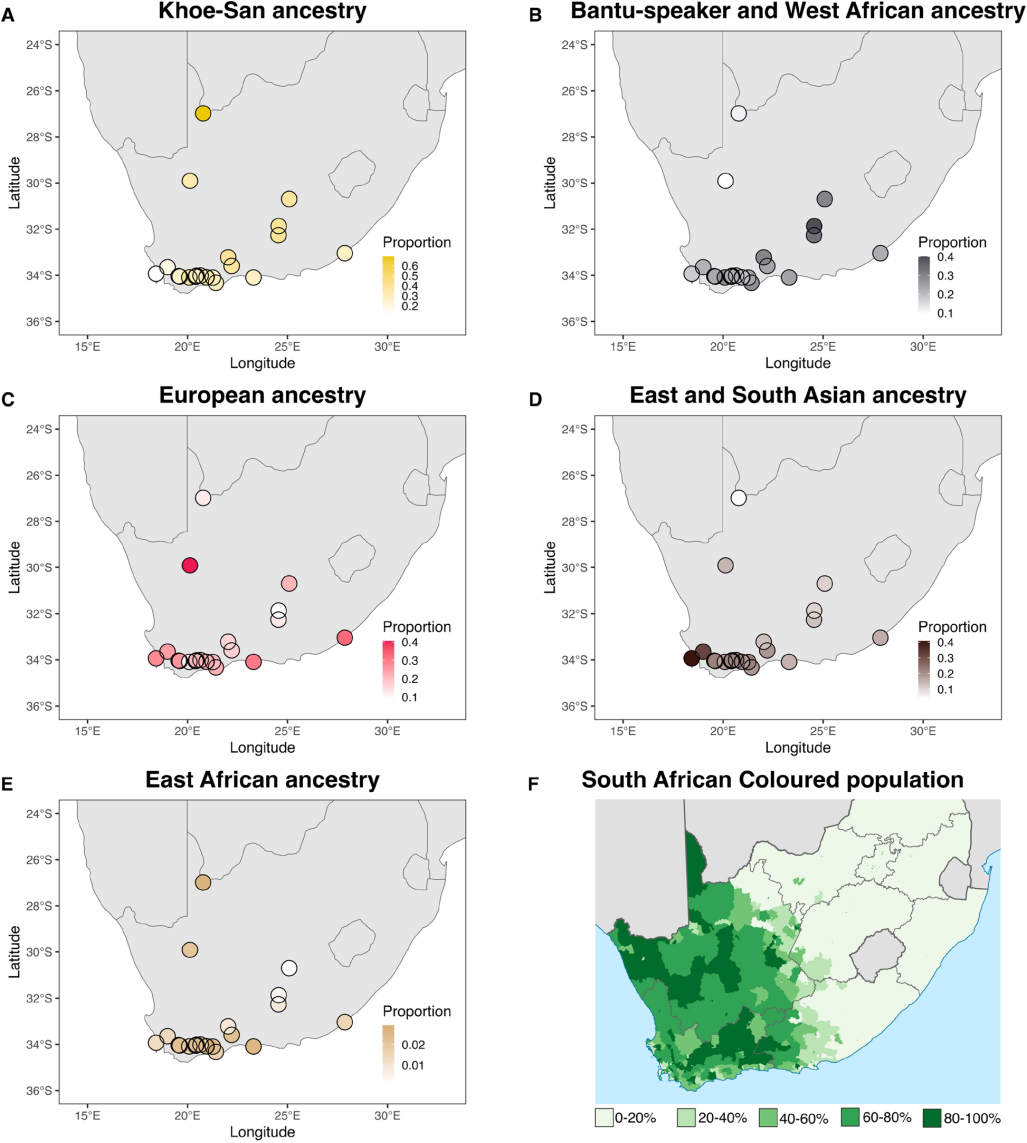
Visualisation of averaged ADMIXTURE derived ancestry proportions from *K* = 6 plotted by sampling locations. For each ancestry, the average ancestry proportion as reported by ADMIXTURE was calculated per site and visualized on a map. The colour scale is relative to the maximum value of each fraction of admixture. A depicts the component associated to Khoe-San ancestry, the corresponding is shown for **B** West African and Bantu-speaker ancestry, **C** European ancestry, **D** South Asian and East Asian ancestry combined, and **E** East African ancestry. In **F**, the proportion of SAC people among the inhabitants is shown per region in South Africa. Based on the 2011 census. Adapted from https://commons.wikimedia.org/wiki/File:South_Africa_2011_Coloured_population_proportion_map.svg, Public Domain

As the ADMIXTURE at *K* = 6 captures best the diversity in ancestries in the SAC, the average ADMIXTURE derived ancestral fractions for *K* = 6 were plotted on a map of the southern part of South Africa, to investigate spatial patterns of the different major ancestries (Fig. 2). The ancestry fractions were also plotted against latitude and longitude (Additional File 12: Fig. S12) and a linear model was fitted through the data. Khoe-San ancestry is larger towards the inland regions and towards the east, with the linear model for latitude having a significant *p*-value of 0.000259. Bantu-speaker ancestry proportions are higher in the most eastern localities (*p*-value of the linear model describing the correlation with longitude is significant at 0.0311). The combined East and South Asian ancestry is highest close to Cape Town and decreases the more north and east one goes (*p*-values of 0.00197 and 0.00467, respectively). East African ancestry is smaller than the other ancestries (0.1–2.9%), but geographical differences can be observed with higher East African ancestries along coastal regions and along the Gariep river valley (northern-most point). The correlation of East African ancestry with longitude is significant (*p*-value = 0.0239). The European-related ancestry is highest along the coast, with the exception of the Northern Cape site. The correlations of European-related ancestry to latitude and longitude are not significant. To account for biases due to small sample sizes at individual locations, we performed the same analysis, removing sites with fewer than 10 individuals (Additional File 13: Fig. S13. Four sites were removed, and all *p*-values that were significant in the previous analysis, remained significant. Additionally, in this more stringent analysis, European ancestry correlated significantly (*p*-value of 0.045) with longitude, with higher levels of European ancestry in the west. To investigate the effect of individual sites on the significance of a correlation, we performed a leave-one-out analysis. In this analysis, the *p*-value for the relationship between each ancestry and latitude or longitude was recalculated after removing one site at a time (Additional File 14: Fig. S14). We observe that the Askham site has a big influence on the significancy of the correlation between Khoe-San ancestry and latitude.

### Admixture dating

SAC individuals trace their ancestry to major ancestral groups that might have admixed during different time periods. We employed a local ancestry estimation method to discern the mosaic composition of the genomes of SAC individuals, delineating which segments most likely originated from each parental population. The constructed ancestries typically reflect the major continental ancestries that we get from ADMIXTURE, see the 1-*F*_st_ plots in Additional File 15: Fig. S15 and Additional File 16: Fig. S16 (figures for additional SAC sites available on request). However, in some cases several ancestries belong to the same continental ancestry. One such case is Oudtshoorn, where the fourth and fifth ancestries are closest to the Khomani and Karretjie respectively, both southern Khoe-San groups.

Subsequently, using this information, we retrieved the admixture dates of these parental populations from the co-ancestry curves as generated by MOSAIC and MALDER (Additional File 17: Fig. S17 and Additional File 18: Fig. S18). Most of the dates fall within less than ten generations, overlapping with the time period since European colonisation. Within the MOSAIC analysis, nine SAC populations display admixture dates that are above 50 generations ago (corresponding to 1450 years, assuming a generation time of 29 years [33]). Seven of these older admixture dates are associated with Khoe-San and East African ancestry, two of them with European and South Asian ancestry.

### Patterns of sex-biased admixture

**Fig. 3:**
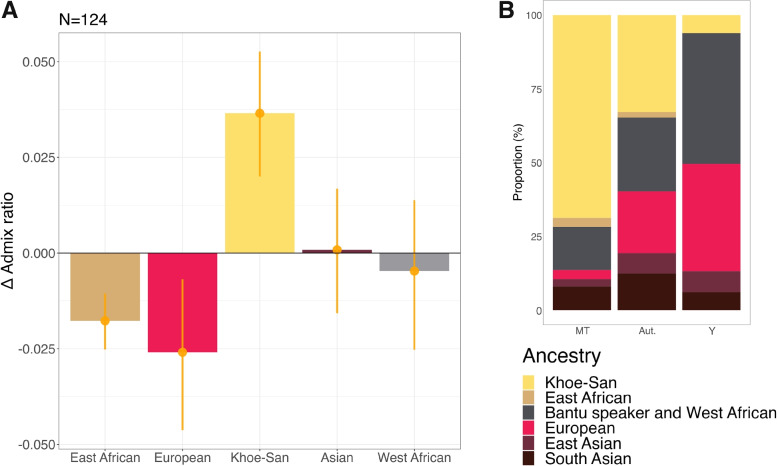
Average sex-biased admixture among the SAC people. In **A**, X-to-autosomal difference ratios for each of the five ancestries, averaged over the seven investigated sites are shown. X and autosomal proportions were bootstrapped (10,000 times) and average X-to-autosomal difference ratios were calculated for each of the five ancestries, as well as standard deviations. The error bars indicate the 95% confidence interval. Negative X-to-autosomal difference ratios are indicative of male-biased admixture for that ancestry, positive X-to-autosomal difference ratios are indicative of a female-biased admixture for that ancestry. Results from sex-biased admixture analyses per site can be found in Additional File 20: Fig. S20. In **B** ancestries associated with the mitochondrial and Y genomes, as well as the autosomal proportions are shown for all studied SAC individuals

Previous studies have shown that the admixture events that shaped the SAC population were sex-biased [13, 27], indicating that the extent of male and female genetic contribution from different admixing populations varied. Here, we investigate the sex-biased nature of the admixture events shaping the SAC populations further by performing supervised ADMIXTURE for the autosomes and X-chromosome (Additional File 19: Fig. S19) and looking at the $\Delta$Admix ratios of East-African, European, Khoe-San, Asian and West-African ancestry (Fig. 3A). We observe negative $\Delta$Admix ratio values with 95% confidence intervals not overlapping zero for East-African and European ancestries (− 0.0177 and − 0.0259, respectively), indicating male-biased admixture from East-Africans and Europeans. We observe a positive $\Delta$Admix ratio with 95% confidence intervals not overlapping zero for Khoe-San ancestry (0.0365), indicating female-biased ancestry from Khoe-San people. For both Asian and West African ancestries, $\Delta$Admix ratios are not significantly differing from zero. This analysis was also performed per site (Additional File 20: Fig. S20), and although all $\Delta$Admix ratios associated with European ancestry are negative and all those associated with Khoe-San ancestry are positive, the 95% confidence intervals often overlap zero, due to smaller sample sizes.

These signals of sex-biased admixture are further supported with data from the uni-parental markers of these individuals. The mitochondrial genome and the Y-chromosome allow for the study of maternal and paternal lineages separately in a population. We generated novel mitochondrial DNA sequences for 72 SAC individuals and determined the Y-chromosome haplogroups for 67 newly genotyped SAC individuals using SNAPPY [34]. We combined these data with the individuals from the reference datasets. Our results show that mitochondrial haplogroups associated with Khoe-San ancestry are more frequent in the SAC populations than the Y chromosome haplogroups associated to Khoe-San ancestry (Fig. 3B). The opposite pattern is observed for West-African and European associated uniparental markers. Haplogroups associated with East-African ancestry become less frequent as we move from mitochondrial genomes to Y chromosomes, but fractions are low (< 0.031). For both Asian ancestries, no clear pattern can be observed. Additional File 21: Fig. S21 shows the associations of the mitochondrial genomes, autosomes and Y chromosomes to the six different ancestries for each of the separate sites. These results are also stated in Additional File 22: Table S3 and S4. Large differences in continental distributions can be observed between sites such as Genadendal, Graaff-Reinet and Askham (numbers of individuals in Additional File 22: Table S5). Genadendal, located in the west, generally shows more European ancestry in autosomes, and more mitochondrial and Y chromosomal haplogroups associated with Europeans. This contrasts with the locations of Graaff-Reinet and Askham, situated more to the east and north, respectively. Elevated Khoe-San ancestry can be observed at Askham for all three genetic markers (MT, autosomes and Y), whereas elevated Bantu-speaker ancestry is evident for all markers in Graaff-Reinet.

## Discussion

In this study, we have analysed genome-wide data from 125 SAC individuals, coming from seven different locations in South Africa. Combining this information with previously published genetic data, our investigation encompasses a thorough examination of ancestral genetic components within the SAC, spanning a wide geographic distribution. We have investigated the complexities of admixture events that shaped the SAC population and explored potential geographic variations in ancestral contributions. We find evidence of geographical stratification of genetic ancestries in agreement with historical information.

### Ancestry proportions in the South African Coloured population

Our analysis of the general genetic background of the SAC population through PCA and ADMIXTURE (Fig. 1) supports the previously identified ancestral components: Khoe-San, West African and Bantu-speaker, European, East African, South and East Asian [13, 26–28]. We identified heterogeneity within the SAC, with ancestry proportions differing substantially across individuals (Fig. 1B and C). This variation likely reflects complex historical and demographic processes, including differential migration patterns, local founder effects, and varying degrees of admixture over time (1652–present), which will include very recent admixture in some individuals.

Results from the ADMIXTURE analysis, Fig. 1B and C, align with previous studies [1, 13, 28] indicating that the primary genetic ancestry found among the SAC is Khoe-San and that the SAC are very heterogeneous. ADMIXTURE at *K* = 8 (Additional File 6: Fig. S6) splits the northern from the southern Khoe-San populations, thereby revealing for the first time that the Khoe-San ancestry in the SAC is mostly southern Khoe-San-related (Nama, Karretjie, and Khomani). ADMIXTURE at *K* = 9 separates the West African ancestral component into a component maximised in West African non-Bantu Niger-Congo speakers (dark brown) and another in Bantu-speaking populations (light grey) (Additional File 6: Fig. S6). The analysis at *K* = 10 reveals low contribution (0.4–3.3%) of the West African ancestry in the SAC. Slaves were brought to the Cape colony from the West African kingdom of Dahomey and from Angola in 1658, and were a part of the founding population of the SAC [22]. However, low West African ancestry in the South African Coloured population can be explained by the historical patterns of the Cape Colony’s slave trade. According to Shell [35], approximately 63,000 enslaved individuals were brought to the Cape between 1658 and 1807. However, only a small proportion originated from West Africa (2.5%), while a much larger share came from the East African coast (26.4%). This limited West African contribution to the enslaved population at the Cape is a key factor in the low levels of West African ancestry observed in the SAC population today. This would be supported by the ADMIXTURE analysis, which reports low West African ancestry contributions among the SAC, and around 22.5% (minimum 7.6, maximum 39.5) of the Bantu-speaker-associated ancestry. According to the ADMIXTURE analysis, these initial enslaved individuals seem to have contributed a small but consistent amount of ancestry to the SAC communities. However, the *f*4-statistics in the form *f*4 (Chimp, SAC, YRI_AFR, Zulu) indicate a greater genetic influence from the West-African Yoruba (Additional File 9: Fig. S9). This mismatch with the ADMIXTURE results has been observed in other studies as well, and is called “neighbour repulsion” [36], where the neighbouring populations (Zulu in this case) received independent gene flow from an external source after their split from the West-Africans. In Afrikaners, the West African non-Bantu Niger-Congo speaker associated component contributes more than the Bantu-speaker associated component [21]. This difference in ancestry contributions based on ADMIXTURE likely reflects different patterns of historical admixture for the SAC and Afrikaner populations. West African admixture into Afrikaners likely occurred during the early phases of founding of the colony, with slaves of West African origin, while most of the West African component in the SAC groups was most likely contributed through continued admixture with Bantu-speakers in the contact zone towards the east.

District Six and Wellington have relatively high South Asian ancestry contributions (Additional File 22: Table S1), likely due to the specific social dynamics at these sites. The District Six community was formed by formerly enslaved people, merchants, and immigrants. Cape Malays, brought as part of the slave trade, composed an essential portion of the founding community, along with the Xhosa people. Afrikaners composed only a small part of the residents of District Six until apartheid laws declared it a “whites-only” area in 1966, causing many people to be forcibly relocated [37]. Today, more than 90% of its inhabitants are SAC [38]. Similarly to District Six, Wellington was founded in 1699 as an agricultural town. Until the first part of the 20th century, it was mainly composed of SAC residents, many of whom were Muslims and of Asian descent [39].

Our ADMIXTURE analysis at *K* = 6 also highlights the genetic contributions of Asian populations to the SAC population. With the average South Asian contribution at 12.1%, it is roughly twice as large as the contribution from East Asians. *F*4-values in the form *f*4 (Chimp, SAC, CHB_EAS, GIH_SAS) are positive for most SAC groups, supporting more admixture from South Asians (Additional File 10: Fig. S10). Thus, we conclude that most of the Asian slaves were brought from South Asia, and to a lesser extent from East Asia. This corresponds to the historical record stating that the Dutch East India Company imported slaves from Indonesia to South Africa [40].

Through ADMIXTURE and subsequent *f*4-statistics, we also elucidate for the first time the contribution of Malagasy populations to the SAC population. At *K* = 11, the Malagasy populations get their own cluster (royal blue), with some minor West-African and East-Asian contributions (Additional File 6: Fig. S6). This Malagasy population genetic cluster can also be observed in the various SAC populations (averaging at 5.8%). We computed *f*4-statistics in the form *f*4 (Chimp, SAC, Vezo, GIH_SAS) (Additional File 10: Fig. S11) and show that there is less genetic affinity of the SAC to the Malagasy, when compared to the South Asian population. This is not to say that no admixture occurred with Malagasy people, just that South Asians have made a larger contribution compared to the Malagasy contribution. The Malagasy ancestry found in the SAC population is also consistent with the historical record that the Dutch East India Company imported slaves from Madagascar to South Africa [40].

### Regional differences in observed ancestry proportions

We collected data from seven new locations to further identify regional variations in SAC ancestries. While the average continental ancestry is generally comparable across sites, some regional variation is evident. This may be shaped by historical settlement patterns, trade routes, and localized genetic drift, as well as differences in colonial and post-colonial demographic influences. The ancestry proportions at *K* = 6 on a map of South Africa (Fig. 2 and Additional File 12: Fig. S12 and Additional File 13: Fig. S13) reveal various interesting trends. The Bantu-speaker ancestry shows higher contributions in the East, and lower contributions in the West. This can largely be attributed to the dominant presence of Bantu-speaking groups in the eastern regions of South Africa, where the Fish River marks the historical limit of the Bantu expansion [9, 22]. The high prevalence of Khoe-San ancestry in the SAC in the inland regions and toward the east reflects the influence of the Cape colony and the increased admixture from Europeans and enslaved people from Asia and Madagascar in the areas closer to the coast and to Cape Town. In the Northern Cape region, Khoe-San ancestry is high (33.4–69.0%), and Bantu-speaker and West African ancestry is low (9.4–11.6%). The SAC of the Northern Cape can be traced back to the Nama herder groups who resided in Namaqualand (South Africa) and Namibia, local San hunter-gatherer groups, and to European settlers who moved into these interior areas. Thus, the Nama people likely contributed to the high Khoe-San genetic ancestry in the SAC individuals in the Northern Cape. This is supported by MOSAIC analyses, which find low F_st_ values for the Nama as a source population for the SAC at Askham and Northern Cape (Additional File 15: Fig. S15 and Additional File 16: Fig. S16). While investigating the effect of individual sites on the significance of a correlation between ancestries and latitude or longitude, we observed that the Askham site has a substantial influence on the significance of the correlation between Khoe-San ancestry and latitude. This suggests that the observed pattern may be strongly driven by the unique characteristics of the Askham site, which should be taken into consideration when interpreting the results. It does not necessarily imply that the correlation is incorrect, but rather that the Askham site may have a disproportionate impact on the overall relationship, and thus caution is needed when applying the findings to other regions. This encourages the inclusion of more SAC populations from inland regions of the Northern Cape province in future studies. The low Bantu-speaker (and West African ancestry) component in Askham and the Northern Cape site points to limited admixture with Bantu-speakers. The distribution pattern for combined Asian ancestries and, to a large extent, European ancestry exhibits an interesting contrast. In the Cape region, high contributions from Asian and European ancestries can be observed, gradually decreasing as one moves eastwards. This phenomenon finds its roots in the historical influx of European settlers into the Cape Colony, with its centre and entry point at the Cape of Good Hope (current-day Cape Town), accompanied by enslaved people from Asia and other parts of Africa. Although East African ancestry proportions are very low in comparison to the other ancestries, it is higher along coastal regions and along the Gariep river valley (northern-most point) correlating with the past distribution of Khoekhoe herder groups (vs. San hunter-gatherer groups) [8, 41].

### Dating admixture events in the SAC population

Since the SAC individuals trace their ancestries to various continental and sub-continental sources, we set out to investigate when these populations admixed. We identify that most of the admixture dates fall within less than ten generations, aligning with the anticipated timeframe for the formation of the Cape Colony. The admixture dating using MOSAIC (Additional File 17: Fig. S17) also identified several admixture events that can be correlated with the formation of the Khoekhoe with the arrival of East African pastoralists in southern Africa [13, 42–44]. This can be seen in a few dates that are very old (> 50 generations ago), which has previously been shown in a study by Vicente et al. [13]. These exact dates should, however, be viewed with caution as they are based on small fractions of ancestry, have deep time estimates and have parental source groups that might be distant from actual source groups. This uncertainty is reflected in the co-ancestry graphs that the dates are inferred from, in which the Khoe-San vs. East African admixture estimates are the least robust of the analyses (figures available on request). The East African ancestry contribution is the smallest according to the ADMIXTURE results (Additional File 22: Table S1) and minor ancestries are problematic for the proper fitting of co-ancestry curves. Two of the admixture dates older than 50 generations ago can be attributed to European and South Asian ancestries, reflecting admixture events happening outside the African continent before these ancestries were introduced during colonial times, possibly related to Eurasian trade routes such as the Silk Road (200 BCE–1450 AD). The earlier admixture dates identified by MOSAIC could not be independently verified using a MALDER analysis (Additional File 18: Fig. S18). This discrepancy likely stems from the fact that these earlier admixture events involve only small ancestry proportions. While both MOSAIC and MALDER can analyse multiple admixture events, MOSAIC can process data from multiple source populations without requiring pre-specified surrogate populations. Moreover, MALDER is based on the decay of linkage disequilibrium (LD) over time, which allows it to estimate admixture dates but may be less sensitive to complex or multiple overlapping events. MOSAIC, on the other hand, estimates admixture dates by performing local ancestry inference. This can provide more detailed results by explicitly modelling the ancestry tracts along the genome. The discrepancy between the two methods for older admixture events could arise because LD-based methods such as MALDER, are more affected by genetic drift and subsequent recombination, which can obscure older signals. Given that MOSAIC directly reconstructs ancestry tracts, it may be better suited for resolving complex or ancient admixture histories.

The admixture events with Bantu-speakers (unlabelled in Additional File 17: Fig. S17) mostly occurred during and after colonial times. This indicates, for the first time in the SAC, that most of the admixture between Khoe-San and Bantu-speakers also occurred after colonial times, most likely due to the disruptions and population mobility that the colonial times instigated. Even the admixture events between Asian and West-African/Bantu-speaker ancestries are all between 3.9 and 11.7 generations ago. Malagasy populations are known to be the result of an admixture event between Austronesian and Bantu sources around 20 to 32 generations ago [45]. These sources are supported by the ADMIXTURE analysis in this study (Fig. 1B and C). However, we do not observe the same generation time-frame for the admixture event between Asian and West-African/Bantu-speaker ancestries in the SAC individuals, possibly indicating that most of these ancestries came from Asian, West-Africans, and Bantu-speakers directly, and not from Malagasy populations. This observation fits with the small Malagasy contributions observed at *K* = 11 (Additional File 6: Fig. S6) and is in line with what has been observed in other SAC populations [13].

### Sex-biased nature of admixture events in the South African Coloured

Unequal power relationships such as colonialism and enslavement can lead to sex-biased admixture. This was also the case among the SAC. There were disproportionately few women among the European settlers [22] and Khoekhoe women constituted the majority of the maternal contribution for the SAC groups [27]. Additional investigations into the sex-biased nature of the admixture events shaping the SAC using X-chromosome and autosomes inferred a male-biased influence from East Africans, Asians, and Europeans, and a female-bias from Khoe-San and West-African individuals [13]. In the current study, we also observe a male-biased admixture from East Africans and Europeans, and a female-biased admixture from Khoe-San. The Asian ancestry shows a very small female bias and the West-African ancestry a male bias (both trends are not statistically significant). The investigation of sex-biased patterns at individual sites (Additional File 20: Fig. S20) highlights the heterogeneous nature of the SAC population. Although non-significant, West-African sex-biased admixture ratios are female-biased in some sites (Genadendal, Greyton, Oudtshoorn, and Kranshoek), while being male-biased in other, more northeastern sites (Graaff-Reinet, Nieu-Bethesda and Prince Albert). This suggests that different social structures or patterns influenced sex-biased admixture across sites. As such, patterns observed at individual sites should not be used to generalise about the entire SAC population, as each site may reflect distinct local dynamics. The sex-biased admixture in the SAC is supported by the findings from uniparental markers; mitogenomes and Y chromosomes. Mitochondrial haplogroups associated with Khoe-San ancestry are more frequent in the SAC populations than the Y chromosome haplogroups associated to Khoe-San ancestry (Fig. 3B). The opposite pattern can be observed for West-African and European associated mitochondrial and Y chromosome haplogroups. Since the mitochondrial genome is inherited through the female line and Y chromosome completely through the male line, we observe them as the extremes when it comes to differences between the contribution of the two sexes, whereas the autosomal ancestries are observed somewhere in the middle of these two. We also note the regional variation between the male and female contributions from different populations across the sites (Additional File 21: Fig. S21), again highlighting the genetic heterogeneity of the SAC population. Apartheid-era legislation in South Africa, particularly the Prohibition of Mixed Marriages Act (1949) and the Immorality Act (1950), legally enforced racial segregation by prohibiting marriages and sexual relationships between individuals classified as belonging to different racial groups. These restrictions likely contributed to genetic discontinuities by reducing opportunities for gene flow between populations during this period.

Due to its unique inheritance pattern, the X chromosome has a smaller effective population size, making it more susceptible to genetic drift compared to autosomes, especially in small or bottlenecked populations. This increased stochasticity is important to consider when interpreting sex-biased admixture ratios. In the current study, we also analyse uniparental markers such as mtDNA and the Y chromosome to validate the patterns observed in X-to-autosomal ratios. mtDNA and Y chromosome estimates are however even more influenced by genetic drift, compared to the X-to-autosomal ratios.

### Implications for anthropology and public health

The genetic data of the SAC population supports the distinctiveness of their culture; the Fst values indicate a clear genetic grouping, showing that despite their heterogeneity, they form a distinct cluster at the genetic level. This reinforces that their cohesion is not solely cultural but also rooted in shared genetic characteristics, originating from shared parental population contributions. Moreover, our study highlights the substantial genetic heterogeneity within a population that sometimes is considered as a homogeneous population group. This has important implications for public health research, particularly in the design and interpretation of case-control studies. A failure to account for this genetic diversity may lead to confounding effects, reducing the validity of associations between genetic markers and disease phenotypes. To mitigate these issues, it is essential to carefully match case and control groups, ideally using individuals from geographically proximate populations that share similar ancestry proportions as we show that there is a significant positive correlation between Fst values and geographic distance. This approach can help minimise population stratification and improve the robustness of genetic epidemiological studies. Beyond public health, our findings also contribute to anthropological research by shedding light on historical patterns of population structure and admixture. The genetic diversity we observe reflects complex social histories, including patterns of migration, marriage restrictions, and cultural practices. Understanding these historical influences can provide deeper insights into how genetic and social factors have shaped South African populations over time. Future interdisciplinary research integrating genetics with historical and ethnographic data may further elucidate the intricate relationship between genetic variation and sociocultural processes in the SAC.

### Limitations due to sample size and sample distribution

One limitation of this study is the relatively small, although biggest to date, sample size of 356 individuals. While this sample is sufficient for our analyses, it may not allow the detection of more subtle patterns. Moreover, small sample sizes at specific locations can bias the conclusions.

Additionally, as we show that the SAC are quite heterogenous, the limited sample size and geographical representation may impact the generalizability of the results. Expanding the samples to include a broader (geographical) representation of SAC individuals would mitigate these issues. Another important consideration is the potential for biases in the sampling process. For example, voluntary participation might introduce selection bias, as individuals who choose to participate may differ systematically from those who do not. Similarly, logistical constraints in recruitment and sampling may have led to overrepresentation or underrepresentation of certain geographical regions or subpopulations within the SAC. Future studies should aim to address these limitations by continuing to improve geographical representation. Nevertheless, the current study provides valuable insights and establishes a foundation for future work.

## Conclusions

In this study, we analysed new genotype array data for 125 South African Coloured individuals and built upon research to describe the genetics of one the most admixed populations in the world, the SAC. The Khoe-San people, especially the southern Khoe-San, played a major role in the foundation of the SAC, with their ancestry contribution ranging from 12.0 to 69.0% across all investigated sites. By adding genetic data from seven new geographically dispersed sites, we were able to better investigate geographical differentiation in ancestry proportions and we identified higher Khoe-San contribution in inland regions and toward the east, and higher Bantu-speaker contributions in eastern regions, whereas the Asian ancestry is higher in western regions. Near Cape Town and in the Western Cape province, the non-African ancestry is especially high, reflecting the historically greater density of European colonists and slaves in those locations. We infer that the admixture events shaping the SAC were in many ways sex-biased; mainly female-biased from Khoe-San people and male-biased from both East Africans and Europeans. Altogether, this study highlights the intricate admixture history and diverse ancestry of the SAC population.

## Methods

### Sampling and genome-wide SNP typing

Saliva samples were obtained with written informed consent, from 152 SAC individuals from seven sites in South Africa using an Oragene DNA OG-500 kit; two in the Eastern Cape Province (Graaff-Reinet($N=45$) and Nieu-Bethesda($N=20$)), and five in the Western Cape Province (Genadendal ($N=29$), Greyton ($N=16$), Kranshoek ($N=11$), Oudtshoorn ($N=17$), and Prince Albert ($N=14$)). These locations were chosen by co-author MDJ (anthropologist), who had active research and contacts in the studied areas. The locations are located in regions where the SAC are dominant, and at the same focusing on a good geographical representation. Sample collection of SAC, Khoe-San and Khoe-San descendent groups was approved by the University of the Witwatersrand Human Research Ethics board, clearance numbers M980553 and M180655, with renewals M050902, M090576, M1604104, and approved by the National Ethics review board of Sweden, clearance number Dnr2021-01448.

DNA was extracted using the prepIT L2P extraction protocol and followed the procedure described in [13]. The data were generated in four genotyping runs on the Illumina Infinium^TM^ H3Africa Consortium Array by the SNP&SEQ Technology Platform in Uppsala, Sweden. Datasets were analysed using GenomeStudio 2.0.3 and aligned to the Human Genome build version 37 (hg19). A total of 2,267,346 SNP markers were collected in genotyping run 1, 2, and 3, and 2,271,503 SNP markers were collected in run 4.

### Quality filtering and autosomal dataset merging

Genotype data from 152 SAC individuals was merged with the same dataset as used in [13, 13, 46–52] and with data from additional sources [1, 21, 28, 45] (see Additional File 22: Table S6). These reference populations were selected based on findings from previous studies. The Petersen dataset was converted to hg37 positions with the LiftOver tool from the University of California Santa Cruz (https://genome.ucsc.edu/cgi-bin/hgLiftOver). PLINK v1.90b4.9 [53] was used to carry out data processing and quality filtering. Before merging the datasets, duplicate SNPs were removed, only overlapping SNPs between datasets were kept, and C/G and A/T SNPs were eliminated to prevent strand flipping errors. Moreover, 5 individuals with genotyping missingness over 15% were excluded and SNPs with less than 10% genotyping rate were excluded. Hardy–Weinberg Equilibrium (HWE) was set to 0.00001 to avoid potential genotyping errors. KING [54] was used to identify relatedness between individuals and 22 individuals were removed because they were related second-degree or closer to another individual in the dataset. SNPs with less than 10% genotyping rate were excluded again. SNPs in LD were removed. Each of the comparative populations was randomly sub-sampled to 30 individuals to avoid a sample-size bias. The final dataset comprised 162 382 SNPs and 1203 individuals, of which 356 were SAC individuals and 125 were newly typed SAC individuals. Geographic information of the SAC individuals from previously published data was obtained from their respective publications (Additional File 1: Fig. S1).

### Population structure inferences

Unsupervised population structure inference analysis for *K* = 2 to *K* = 12 was performed with ADMIXTURE [55] version 1.3.0 using a random seed each time, 50 iterations. PONG version 1.5 [56] was used to visualize the results and find the major mode and pairwise similarity. The optimal *K*-value was determined by averaging the *K*-values reported by ADMIXTURE. Principal component analysis (PCA) was performed using the program smartpca, from the Eigensoft package (version 7.2.1) [57, 58]. Uniform Manifold Approximation and Projection for Dimension Reduction (UMAP) was performed on the genotypes directly using the umap-learn python library version 0.5.3. Fst values were calculated using PLINK for all pairwise population comparisons, and vizualized in a heatmap using the ggplot package in *R*. To assess the correlation between genetic differentiation and geographic distance, a Mantel test was performed using the vegan package in R. Pairwise Fst values between populations were used to construct a genetic distance matrix, while geographic distances were calculated from latitude and longitude coordinates using the Haversine formula. Both matrices were converted to distance objects and compared using a Mantel test (9999 permutations) to evaluate statistical significance.

As the ADMIXTURE at *K* = 6 captures best the diversity in ancestries in the SAC, the average ADMIXTURE derived ancestral fractions for *K* = 6 were plotted on a map of the southern part of South Africa, to investigate spatial patterns of the different major ancestries. The ancestry fractions were also plotted against latitude and longitude and a linear model was fitted through the data using the lm function in *R*. This was done including all 22 sites, as well as only for the 18 sites with sample sizes of at least 10 individuals. To investigate the effect of individual sites on the significance of a correlation, we performed a leave-one-out analysis. In this analysis, the *p*-value for the relationship between each ancestry and latitude or longitude was recalculated after removing one site at a time.

### Phasing, local ancestry estimation and admixture dating

Phasing was carried out out using SHAPEIT version 2.r837 [59] using the 1000 genomes phase 3 reference genomes [49]. Misaligned sites between the reference dataset and the panel were excluded. Local ancestry estimation was performed using MOSAIC version 1.5.0 compiled and ran under R v4.3.2 [60], setting source populations to five (-a 5), to represent the major ancestries in the SAC. MOSAIC was run for chromosomes 1 to 22 (-c 1:22) using 38 panel populations as potential sources (Additional File 22: Table S6). Accuracies of estimated local ancestry (expected r-squared values) are reported in Additional File 22: Table S7. Admixture dates were gathered from the reported dates from the co-ancestry curves. The origin of each reconstructed ancestry was determined through *F*_st_ to the reference populations using MOSAIC. MALDER [61] was also used to estimate admixture dates, using five populations as reference populations (Juhoansi, YRI_AFR, CEU_EUR; STU_SAS), representing the five main ancestries among the SAC.

### Formal tests of admixture

The dataset was merged with a chimpanzee genome and *f*4-statistics were computed using popstats [62, 63] in the format *f*4 (Chimp,SAC,Pop1,Pop2). It was used to test whether SAC individuals were more admixed with Pop1 or Pop2. If the *f*4-value is significantly negative, it implies gene flow between SAC and Pop1 (or Chimp and Pop2). If it is significantly positive, it implies gene flow between SAC and Pop2 (or Chimp and Pop1).

### Sex-biased admixture

X-chromosome/autosomal ratios were computed for the SAC individuals. Genotyping data from the new individuals were merged with a comparative dataset consisting of 20 Central Europeans, 20 Sri Lankan Tamil, 20 Nigerian Yoruba, 20 Ethiopian Amhara and 17 Namibian Ju/’hoansi. The data were filtered as described in the section *Quality filtering and autosomal dataset merging*. To avoid differences in chromosome size affecting the admixture proportions, chromosome 1 to 6 were cut to the length of the X-chromosome (180 centiMorgan), and chromosome 7, 10 and 12 were selected as they roughly have the same length (in centiMorgan) as the X-chromosome. For the autosomes, the number of SNPs was downsampled to the number of SNPs on the X-chromosome (7452 SNPs). Supervised ADMIXTURE (*K* = 5) was run separately for each of the autosomes and the X-chromosome, 50 iterations each [55]. Results were visualized with Pong [56]. The ADMIXTURE results provided the ancestry proportions on the X-chromosome per individual and per ancestry. Average autosomal proportions were calculated from the ADMIXTURE runs of each of the autosomes, for each individual and each ancestry. Female X-chromosomal proportions were weighed twice, as females have two X-chromosomes and males one [64]. Corrected X and autosomal proportions were bootstrapped (10,000 times) and average X-to-autosomal difference ratios were calculated as in [65] for each of the five ancestries as follows:

$$\begin{aligned} \overline{{\Delta }Admix}= F_{anc,total} * (F_{anc,X} -F_{anc,auto})/(F_{anc,X} + F_{anc,auto}) \end{aligned}$$

where $F_{anc,total}$ is the genome-wide admixture proportion for a given ancestry, $F_{anc,X}$ is the X chromosome admixture proportion for a given ancestry and $F_{anc,auto}$ is the autosomal admixture proportion for a given ancestry. Negative X-to-autosomal difference ratios are indicative of male-biased admixture for that ancestry, positive X-to-autosomal difference ratios are indicative of a female-biased admixture for that ancestry.

### Uniparental markers

Barcoded primers [66] were used to amplify the full mitochondrial sequences from 72 SAC individuals. Using a uniquely barcoded primer combination for every sample, we performed a PCR to amplify the whole mitochondrial genome (30× (98 °C, 10 s; 67 °C, 15 min); 4 °C $\infty$) (300 ng DNA, 2.4 nM primers, 200 µM of each dNTP, 1× PCR buffer and 1.25 U Takara LA Taq polymerase in 25 µl reaction). Specificity of PCR products was confirmed on a 1% agarose gel and purified with AMPure PB beads. Concentrations of the cleaned PCR products were measured (Qubit). Samples were pooled (100 ng/sample). The pool was purified with 0.5× volumes AMPure PB beads. Elution was performed in 10 mM Tris-HCl, pH 8.5. Concentration of the cleaned pool was measured on the Qubit. The full mitochondrial genomes were sequenced on the PacBio Sequel II. Demultiplexing of the sequencing data was performed by Uppsala Genome Centre (UGC) at NGI-SciLifeLab using the SMRT analysis pipeline (www.pacb.com/products-and-services/analytical-software/smrt-analysis/). The full mtDNA sequence reads were mapped to the Revised Cambridge Reference Sequence (rCRS, NCBI accession number: NC_012920.1) to create BAM files. These were converted to FASTA files using DeepVariant (version 1.3.0, settings: –model_type=PACBIO) and bcftools consensus (version 1.12). Mitochondrial haplogroups were assigned using HaploGrep3 [67]. All haplogroups were associated with an ancestry, according to literature (see Additional File 22: Table S8).

Y chromosomal haplogroups were assigned for all 119 males using SNAPPY [34] and all haplogroups were associated with an ancestry, according to literature (see Additional File 22: Table S9).

## Supplementary information

Additional file 1. Geographic distribution of sampling locations. In A, locations of all populations including reference populations are shown. In B, a zoom-in of the South African region where the new samples are from is shown. The new SAC locations are shown in light blue, the previously published locations in dark blue.

Additional file 2. Principal Component analysis of the dataset. Within parenthesis is the PC loading. PC3 is plotted against PC2 and PC1, A and B respectively. The new SAC samples are shown in light blue, the previously published SAC samples in dark blue.

Additional file 3. Principal Component analysis depicting the average and standard deviations of the PC values of the populations. On the axes, within parenthesis is the PC loading. In B, a zoom in of the plot with the SAC populations is shown. The new SAC locations are shown in light blue, the previously published locations in dark blue.

Additional file 4. Heatmap showing the Fst values between pairwise population comparisons. Low Fst values (indicating higher genetic similarity) are indicated in red, and high Fst values (indicating lower genetic similarity) are indicated in blue.

Additional file 5. Uniform Manifold Approximation and Projection for dimension reduction (UMAP) of the populations in the dataset. Projections are based on genotype calls. Colours indicate continental ancestry. Labels according to continental groups were added *a posteriori* to help with legibility.

Additional file 6. PONG visualization of unsupervised ADMIXTURE analysis with 50 iterations for the full dataset, for ancestries K = 2 to K = 12. The best identified K through cross validation was K = 10 (Additional File 7: Fig. S7).

Additional file 7. Cross validation (CV) error for K = 2 to K =12, averaged over the 50 repetitions. The K with the lowest CV error was K = 10 (horizontal blue line).

Additional file 8. Values of admixture f4-statistic in the form *f4(Chimp, SAC, Ju/’hoansi, Karretjie)*. Positive values indicate more genetic affinity with Karretjie, negative values indicate more genetic affinity with the Ju/’hoansi.

Additional file 9. Values of admixture f4-statistic in the form *f4(Chimp, SAC, YRI_AFR, Zulu)*. Positive values indicate more genetic affinity with Zulu, negative values indicate more genetic affinity with the Yoruba (YRI_AFR).

Additional file 10. Values of admixture f4-statistic in the form *f4(Chimp, SAC, CHB EAS, GIH SAS)*. Positive values indicate more genetic affinity with GIH_SAS, negative values indicate more genetic affinity with CHB_EAS.

Additional file 11. Values of admixture f4-statistic in the form *f4(Chimp, SAC, Vezo, GIH SAS)*. Positive values indicate more genetic affinity with GIH_SAS, negative values indicate more genetic affinity with Vezo.

Additional file 12. Averaged ADMIXTURE derived ancestry proportions from K = 6 plotted against latitude and longitude. For each ancestry, the average ancestry proportion as reported by ADMIXTURE was calculated per site and then plotted against latitude (left panels) and longitude (right panels). A linear model was fitted through the data and the $R^2$ is indicated, indicating how well the model fits the variance in the data. South and East Asian ancestries were combined in D).

Additional file 13. Averaged ADMIXTURE derived ancestry proportions from K = 6 plotted against latitude and longitude. For each ancestry, the average ancestry proportion as reported by ADMIXTURE was calculated per site and then plotted against latitude (left panels) and longitude (right panels). Sites with less than 10 individuals were removed for this analysis. A linear model was fitted through the data and the $R^2$ is indicated, indicating how well the model fits the variance in the data. South and East Asian ancestries were combined in D).

Additional file 14. *P*-value changes in the leave-one-out analysis. This figure displays the results of a leave-one-out analysis, where the *p*-value for the relationship between each ancestry and latitude or longitude was recalculated after removing one site at a time. Each point represents the *p*-value for a model excluding a specific site, and the dashed red line indicates the *p*-value from the full model (using all sites).

Additional file 15. 1-F_st_ values from MOSAIC for the 5 constructed ancestries for Askham.

Additional file 16. 1-F_st_ values from MOSAIC for the 5 constructed ancestries for Coloured Northern Cape.

Additional file 17. Inferred admixture dates (MOSAIC) for the 5-way admixture scenario for the 22 SAC populations using all reference populations as putative sources. Dots labeled with “EA & KS” indicate admixture events between Khoe-San and East African constructed ancestries, as determined by F_st_. Dots labeled with “EU & SA” indicate admixture events between European and South Asian constructed ancestries. Sites are shown from West (high) to East (low) on the y-axis. X-axis on top shows the time in years, x-axis at the bottom shows time in generations ago.

Additional file 18. Inferred admixture dates (MALDER) for the 5-way admixture scenario for the SAC populations using Juhoansi, YRI_AFR, Amhara, STU_SAS, and CEU_EUR as putative sources. Sites are shown from West (high) to East (low) on the y-axis. X-axis shows time in generations ago.

Additional file 19. Supervised ADMIXTURE results per chromosome, visualized using PONG for K = 5. These ADMIXTURE results were used to calculate the $\Delta$Admix ratios depicted in Figure 3. Each ADMIXTURE run is based on 13,000 SNPs.

Additional file 20. $\Delta$Admix ratios for each of the seven ancestries, shown separately for the seven investigated sites. X and autosomal proportions were bootstrapped (10,000 times) and average X-to-autosomal difference ratios were calculated for each of the five ancestries, as well as standard deviations. The error bars indicate the 95% confidence interval. Negative X-to-autosomal difference ratios are indicative of male-biased admixture for that ancestry, positive X-to-autosomal difference ratios are indicative of a female-biased admixture for that ancestry.

Additional file 21. Maternal, autosomal, and paternal ancestries at each location with SAC individuals. Where no information about maternal or paternal ancestries was available, these are not shown.

Additional file 22: Table S1. Admixture proportions at K = 6 for the 22 SAC populations. Newly investigated sites are denoted in bold. Table S2. Admixture proportions at K = 10 for the 22 SAC populations. Newly investigated sites are denoted in bold. Table S3. Mitochondrial ancestries at the 16 sites for which mitochondrial sequences were available. Newly investigated sites are denoted in bold. Table S4. Y chromosome ancestries at the 22 sites. Newly investigated sites are denoted in bold. Table S5. Number of individuals used at each site to make inferences about mitochondrial, autosomal and Y chromosomal ancestries. Table S6. List of populations included in the dataset. Table S7. Expected r-squared values for the local ancestry inference from MOSAIC for each of the 22 sites. Table S8. Mitochondrial DNA haplogroup assignment by ancestry. Table S9. Y-chromosome haplogroup assignment by ancestry [68–90].

## Data Availability

All data generated or analysed during this study are included in this published article, its supplementary information files and publicly available repositories. The generated genotype data is available for academic research use through the European Genome-Phenome Archive with accession number EGAD50000000513 (152 individuals) and Data Access Committee EGAC50000000240. Scripts are available on Zenodo (DOI:10.5281/zenodo.15622835).

## Abbreviations

CHB_EAS: Han Chinese
GIH_SAS: Gujarati Indians
HWE: Hardy-Weinberg Equilibrium
LD: Linkage disequilibrium
PCA: Principal component analysis
SA: South Africa
SAC: South African Coloured
SNP: Single nucleotide polymorphism
UMAP: Uniform Manifold Approximation and Projection for Dimension Reduction
YRI_AFR: Yorubans
