# Supplementary figures and images for: Wide-scale geographical analysis of genetic ancestry in the South African Coloured population
Source: BMC Biol. 2025 Jul 22;23:219. doi: 10.1186/s12915-025-02317-5 (PMC12281806; doi:10.1186/s12915-025-02317-5)

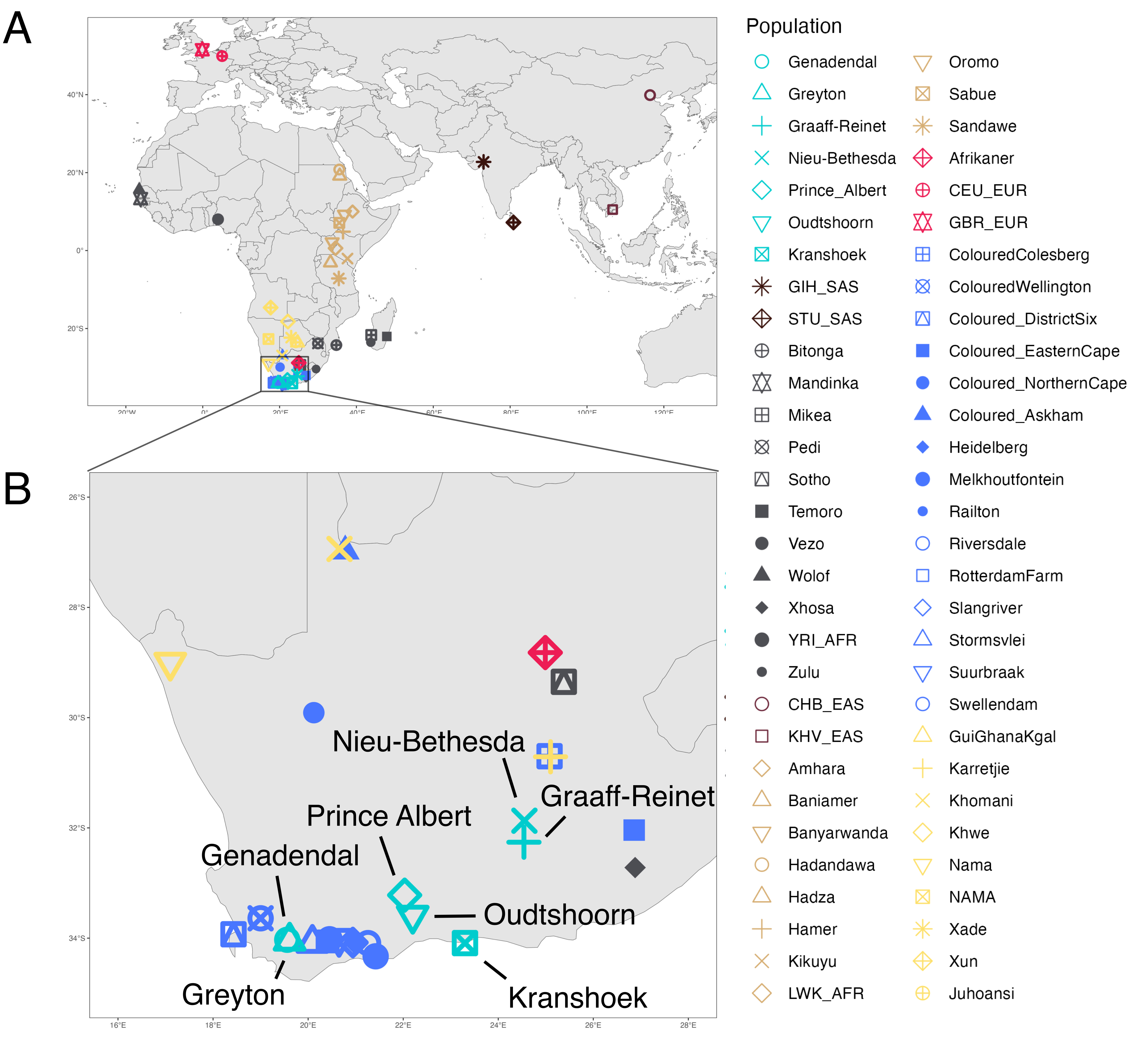

Supplement: Supplementary file 1 — Additional file 1. Geographic distribution of sampling locations. In A, locations of all populations including reference populations are shown. In B, a zoom-in of the South African region where the new samples are from is shown. The new SAC locations are shown in light blue, the previously published locations in dark blue. [file 12915_2025_2317_MOESM1_ESM.jpeg]

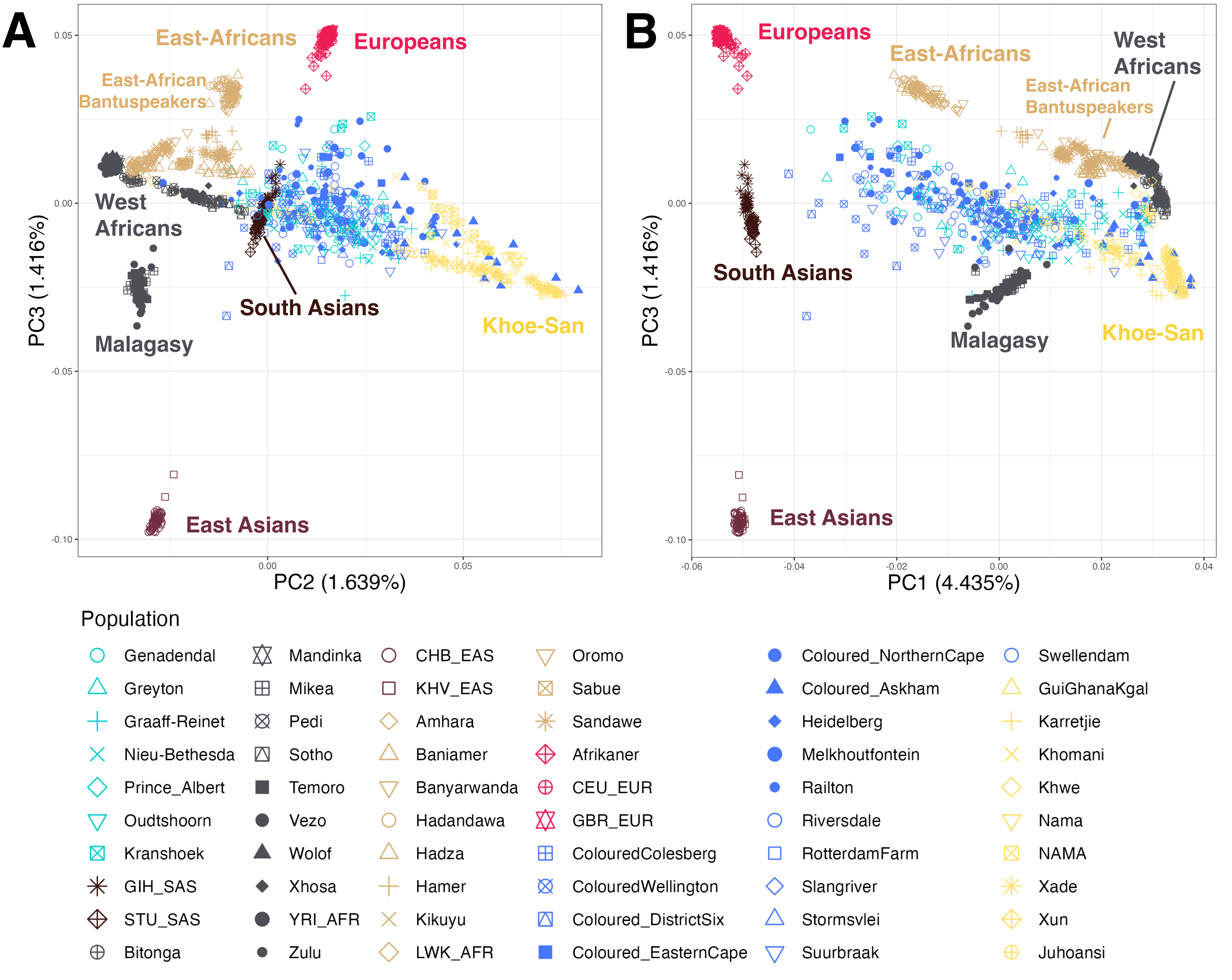

Supplement: Supplementary file 2 — Additional file 2. Principal Component analysis of the dataset. Within parenthesis is the PC loading. PC3 is plotted against PC2 and PC1, A and B respectively. The new SAC samples are shown in light blue, the previously published SAC samples in dark blue. [file 12915_2025_2317_MOESM2_ESM.jpeg]

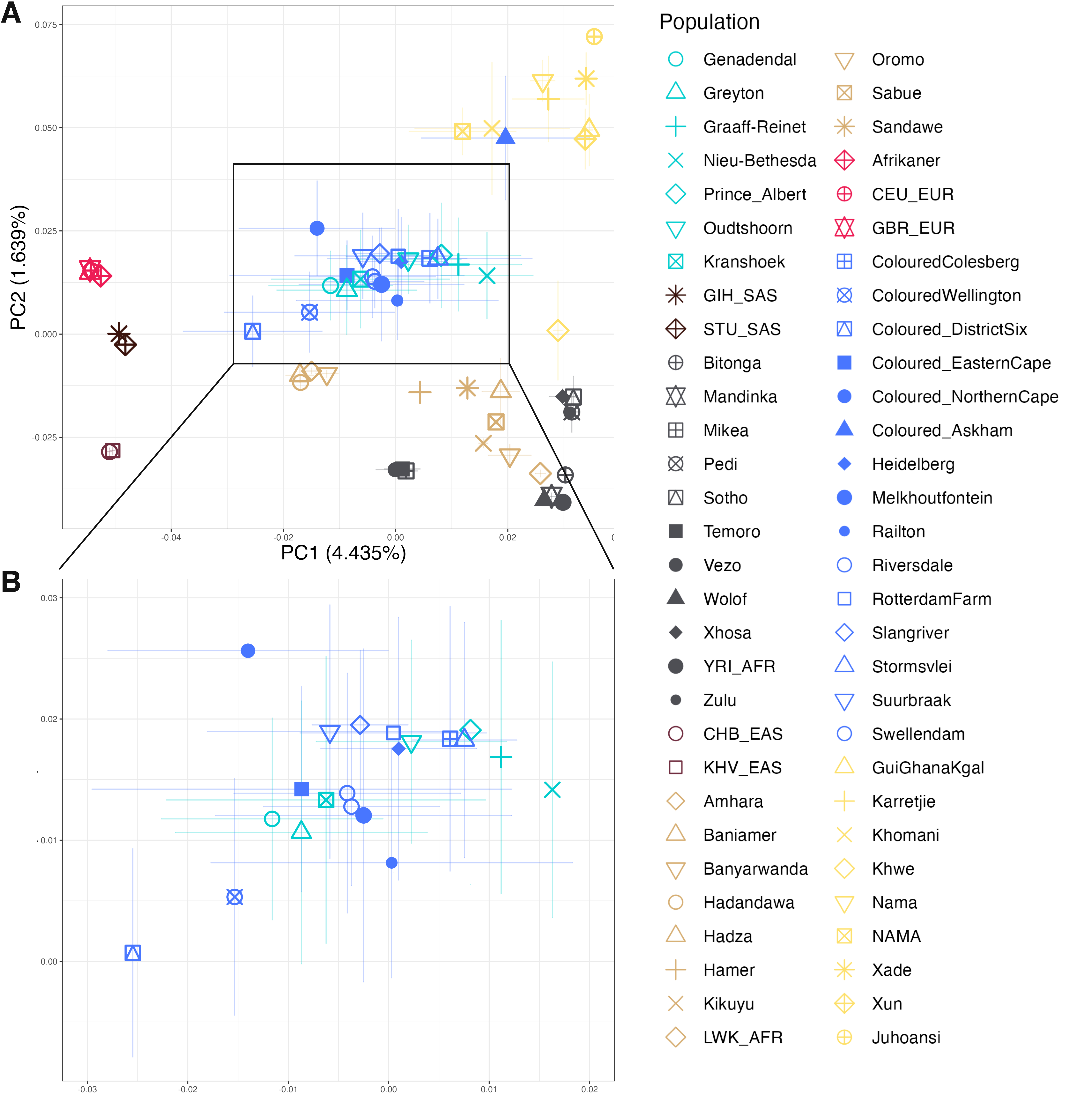

Supplement: Supplementary file 3 — Additional file 3. Principal Component analysis depicting the average and standard deviations of the PC values of the populations. On the axes, within parenthesis is the PC loading. In B, a zoom in of the plot with the SAC populations is shown. The new SAC locations are shown in light blue, the previously published locations in dark blue. [file 12915_2025_2317_MOESM3_ESM.jpeg]

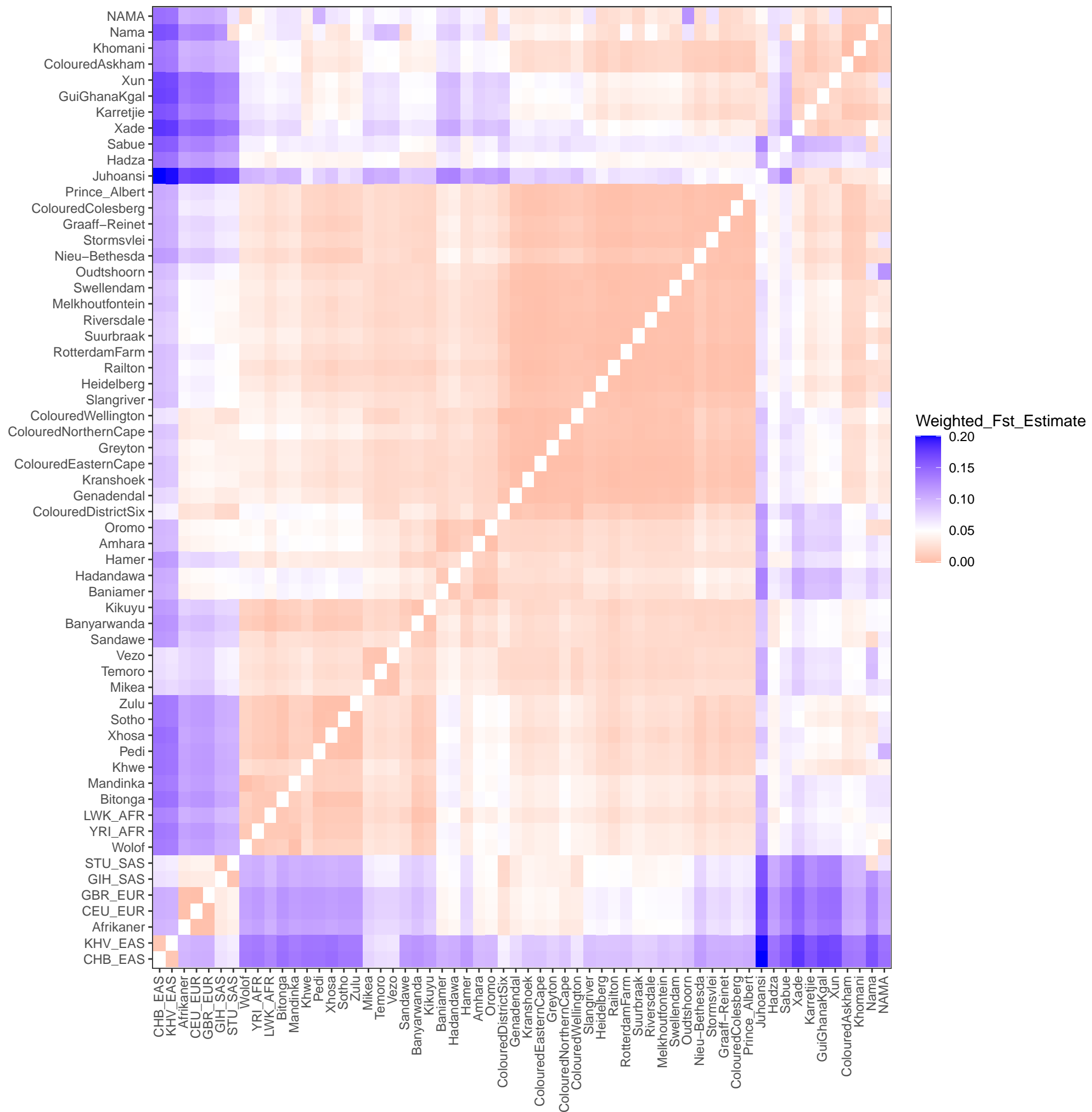

Supplement: Supplementary file 4 — Additional file 4. Heatmap showing the Fst values between pairwise population comparisons. Low Fst values (indicating higher genetic similarity) are indicated in red, and high Fst values (indicating lower genetic similarity) are indicated in blue. [file 12915_2025_2317_MOESM4_ESM.pdf]

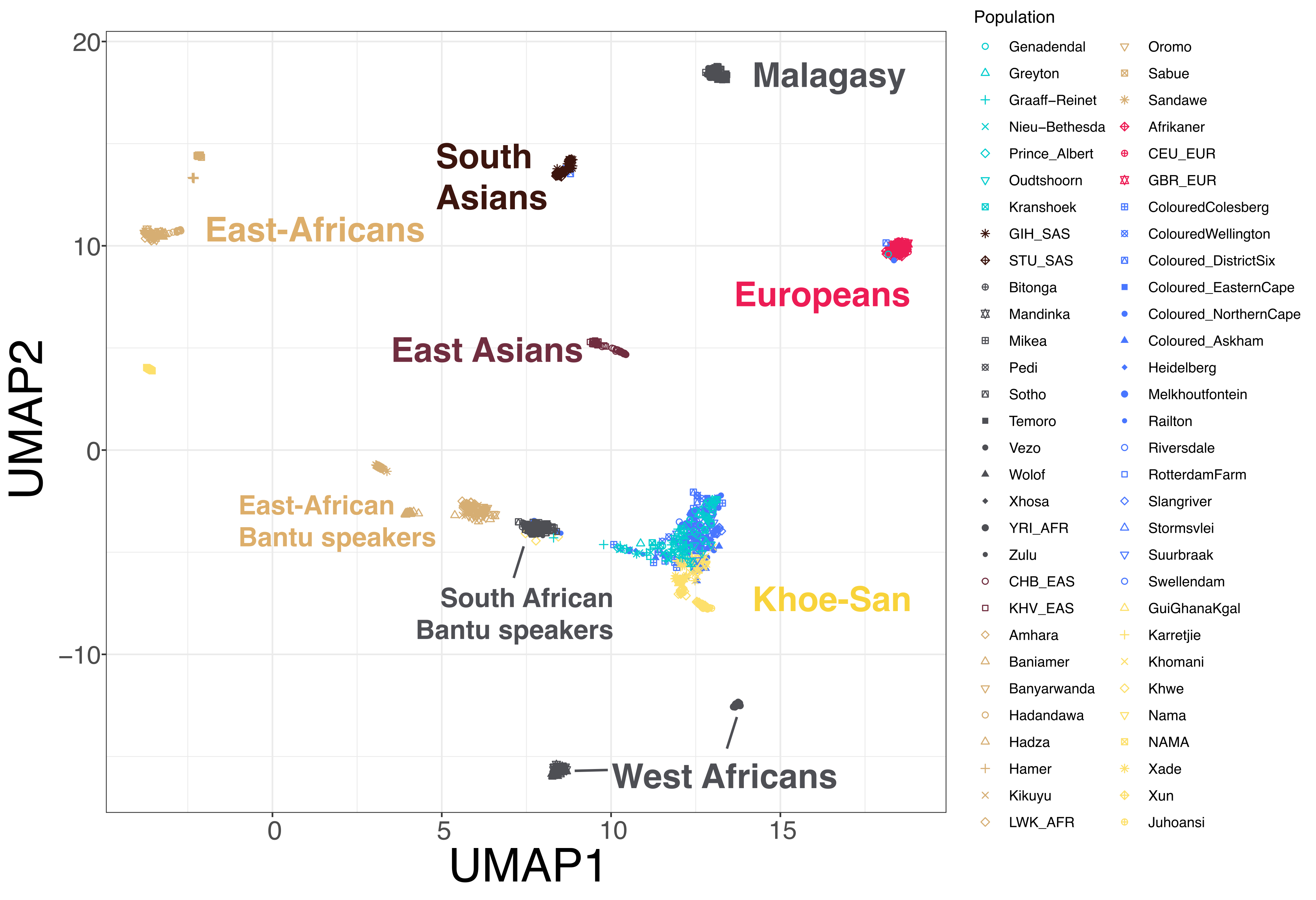

Supplement: Supplementary file 5 — Additional file 5. Uniform Manifold Approximation and Projection for dimension reduction (UMAP) of the populations in the dataset. Projections are based on genotype calls. Colours indicate continental ancestry. Labels according to continental groups were added a posteriori to help with legibility. [file 12915_2025_2317_MOESM5_ESM.jpeg]

Cross-validation error

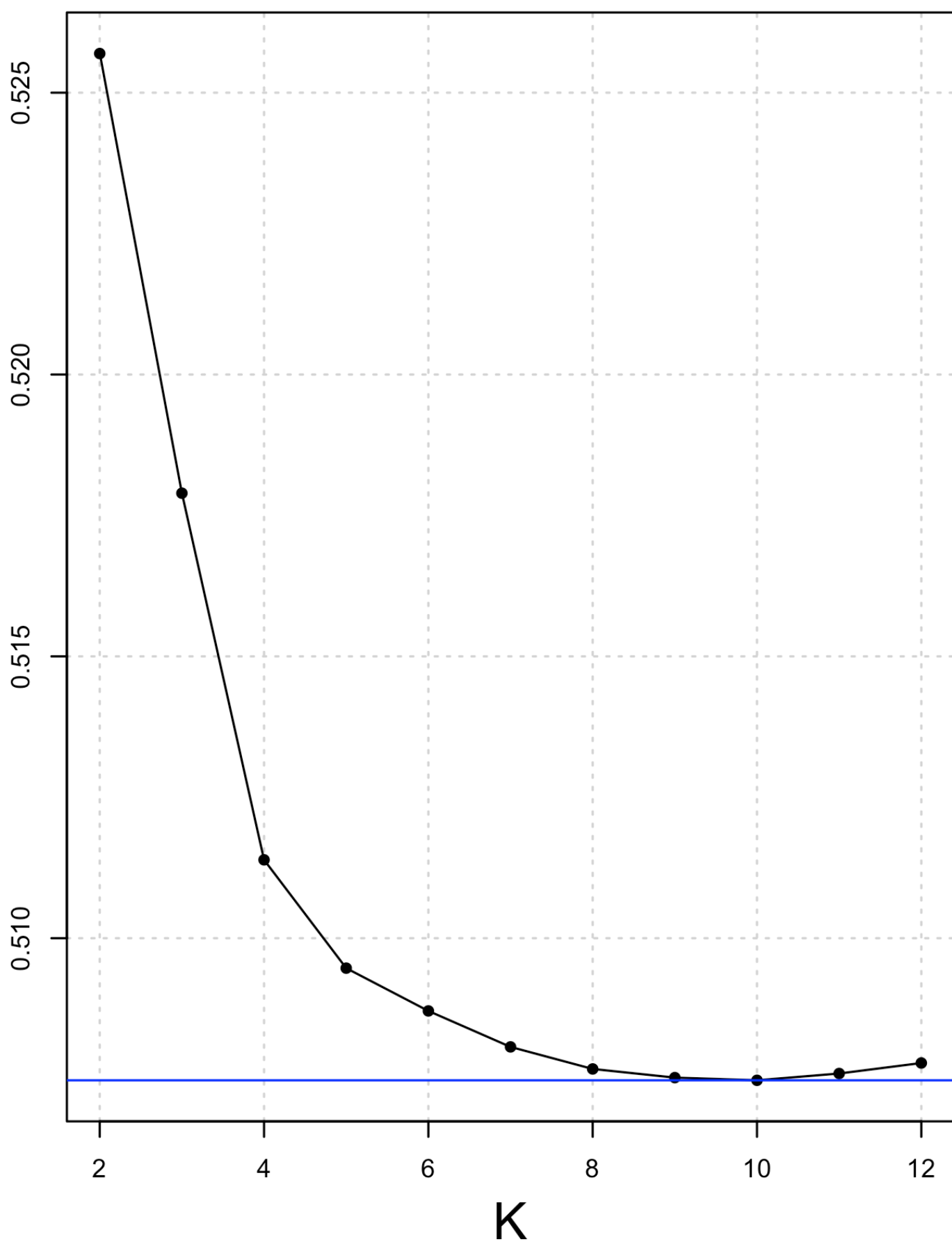

Supplement: Supplementary file 7 — Additional file 7. Cross validation (CV) error for K = 2 to K =12, averaged over the 50 repetitions. The K with the lowest CV error was K = 10 (horizontal blue line). [file 12915_2025_2317_MOESM7_ESM.pdf]

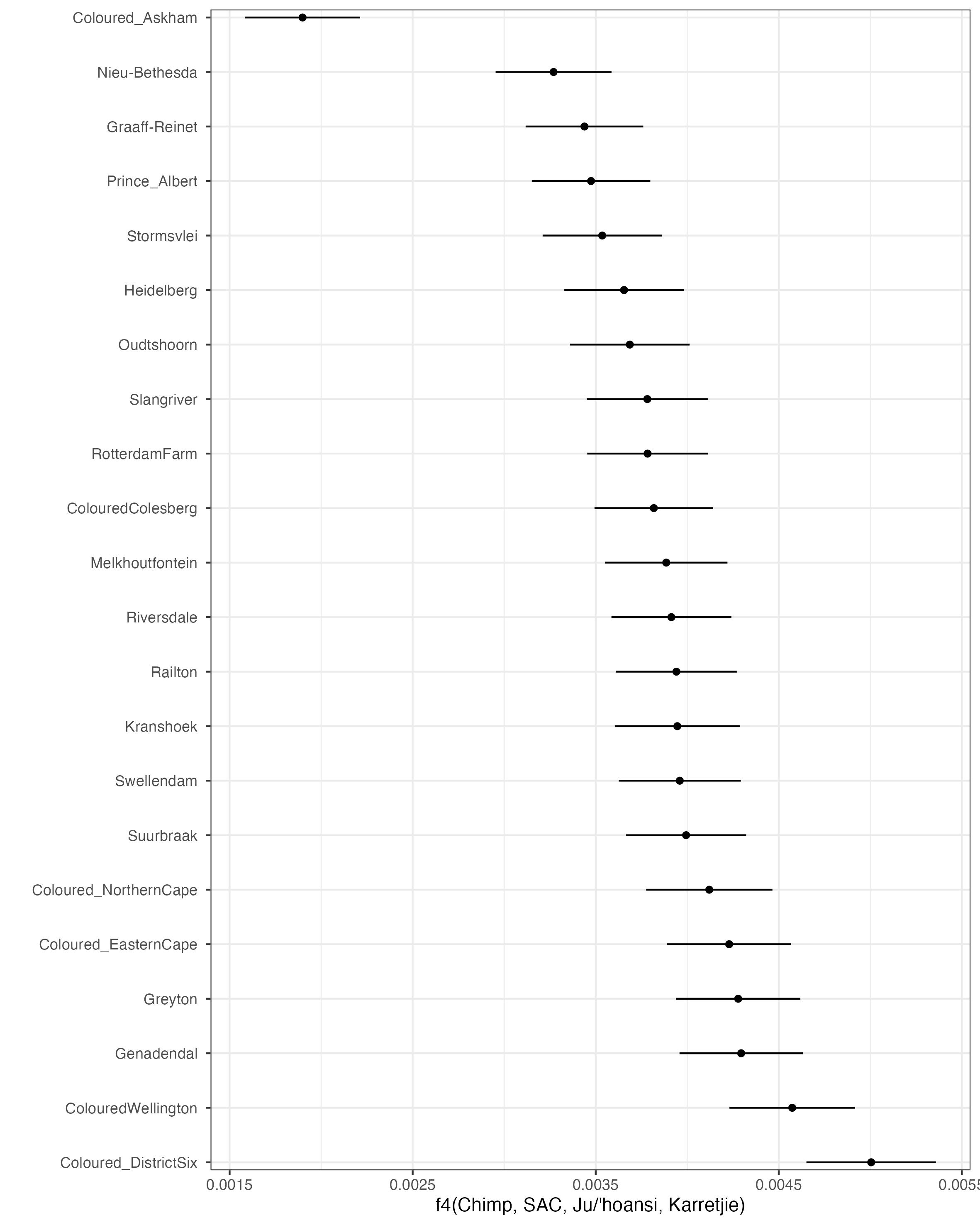

Supplement: Supplementary file 8 — Additional file 8. Values of admixture f4-statistic in the form f4(Chimp, SAC, Ju/’hoansi, Karretjie). Positive values indicate more genetic affinity with Karretjie, negative values indicate more genetic affinity with the Ju/’hoansi. [file 12915_2025_2317_MOESM8_ESM.jpeg]

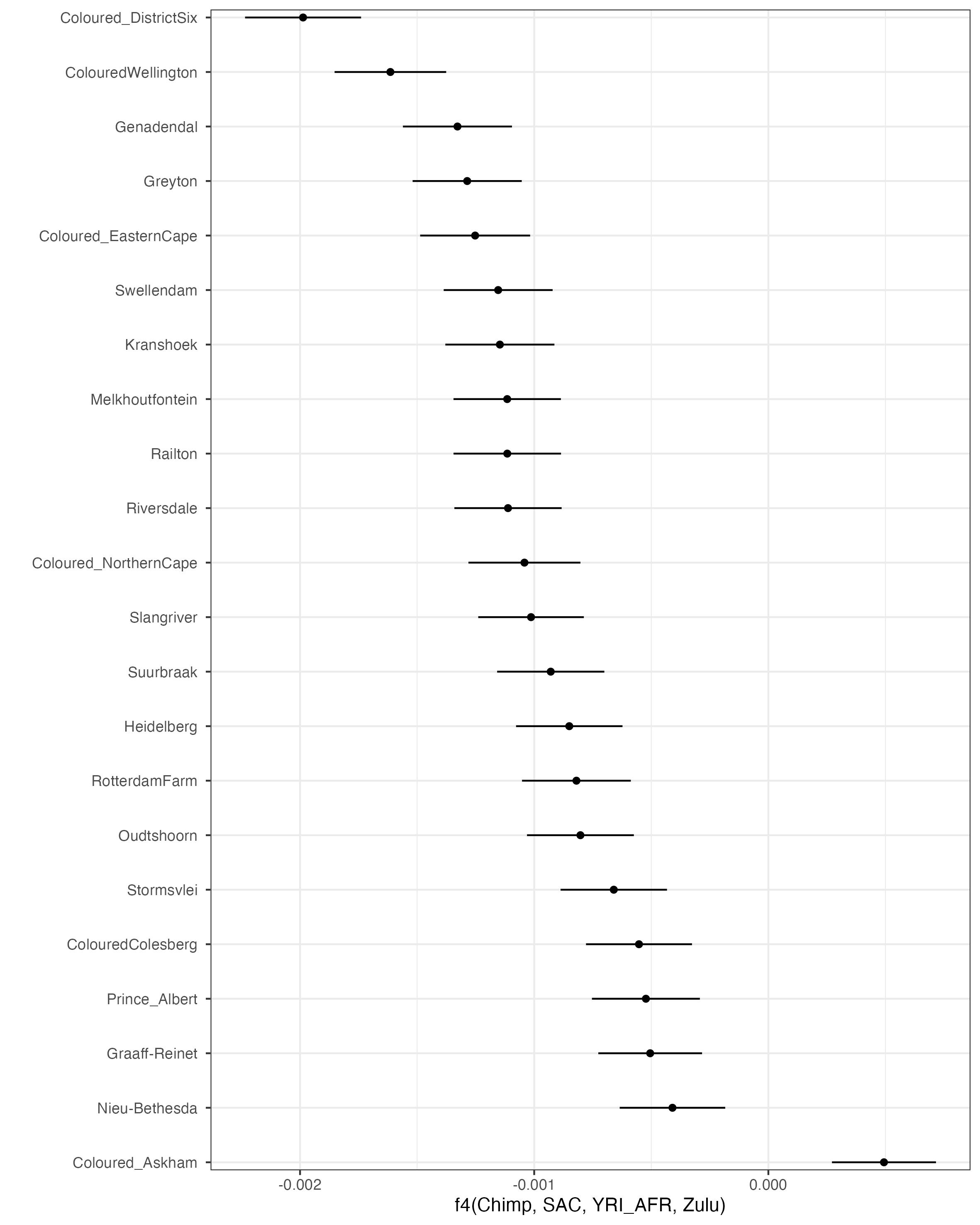

Supplement: Supplementary file 9 — Additional file 9. Values of admixture f4-statistic in the form f4(Chimp, SAC, YRI_AFR, Zulu). Positive values indicate more genetic affinity with Zulu, negative values indicate more genetic affinity with the Yoruba (YRI_AFR). [file 12915_2025_2317_MOESM9_ESM.jpeg]

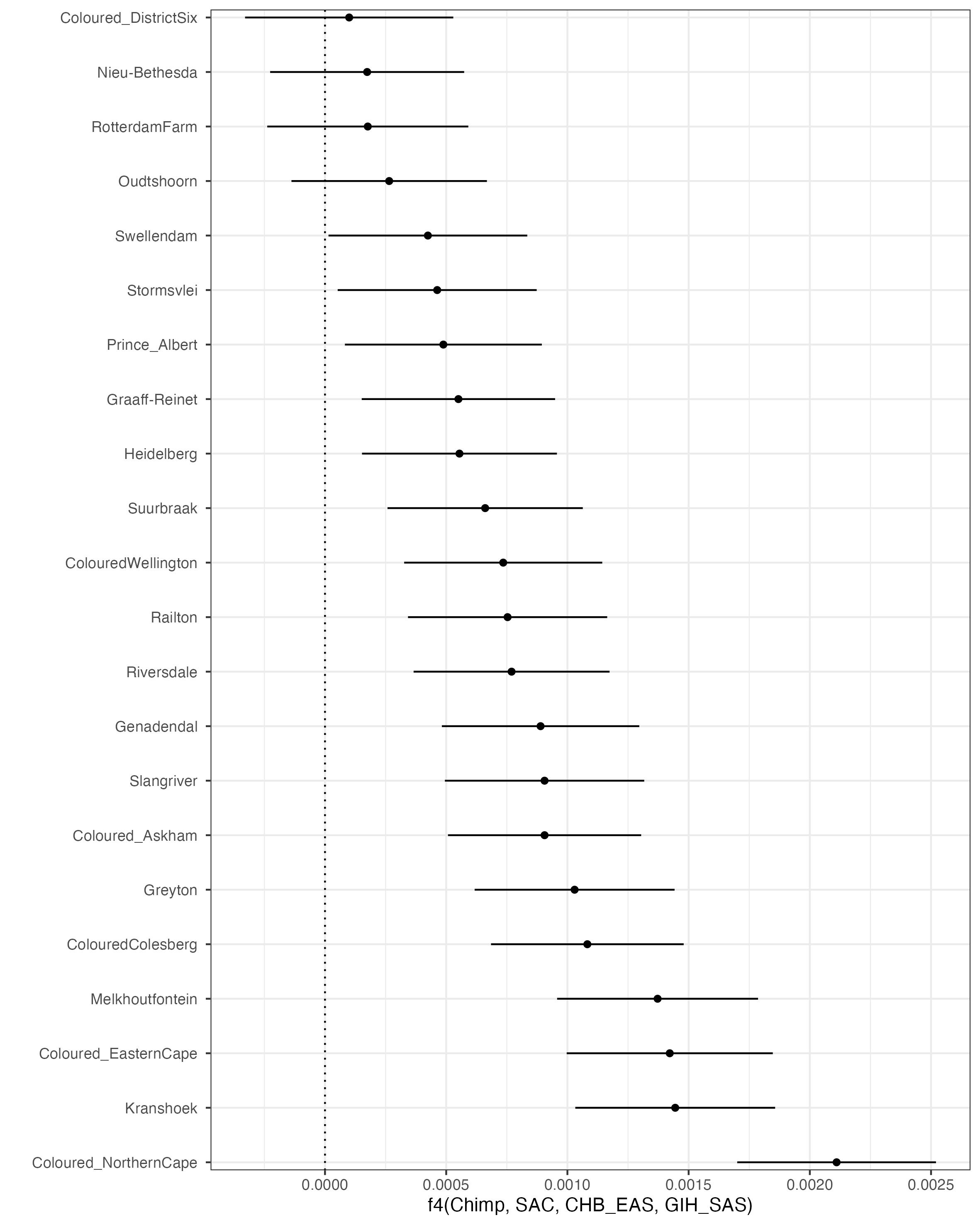

Supplement: Supplementary file 10 — Additional file 10. Values of admixture f4-statistic in the form f4(Chimp, SAC, CHB EAS, GIH SAS). Positive values indicate more genetic affinity with GIH_SAS, negative values indicate more genetic affinity with CHB_EAS. [file 12915_2025_2317_MOESM10_ESM.jpeg]

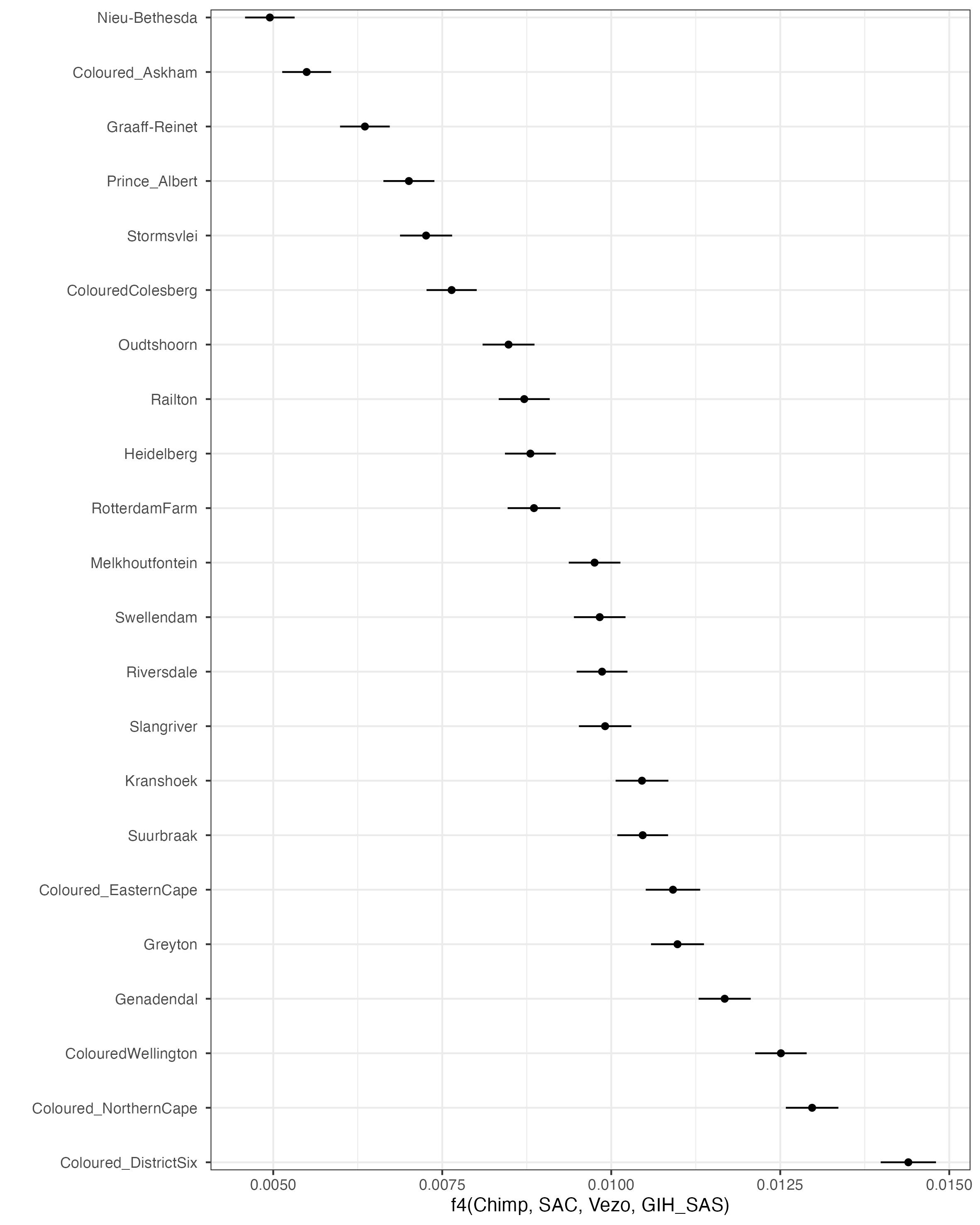

Supplement: Supplementary file 11 — Additional file 11. Values of admixture f4-statistic in the form f4(Chimp, SAC, Vezo, GIH SAS). Positive values indicate more genetic affinity with GIH_SAS, negative values indicate more genetic affinity with Vezo. [file 12915_2025_2317_MOESM11_ESM.jpeg]

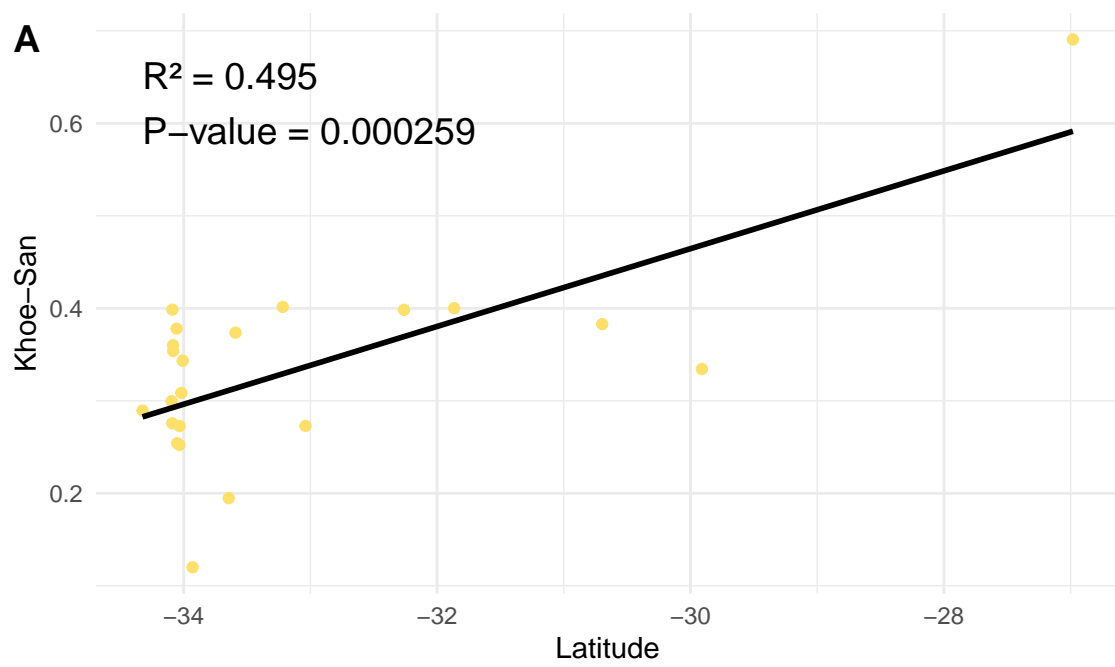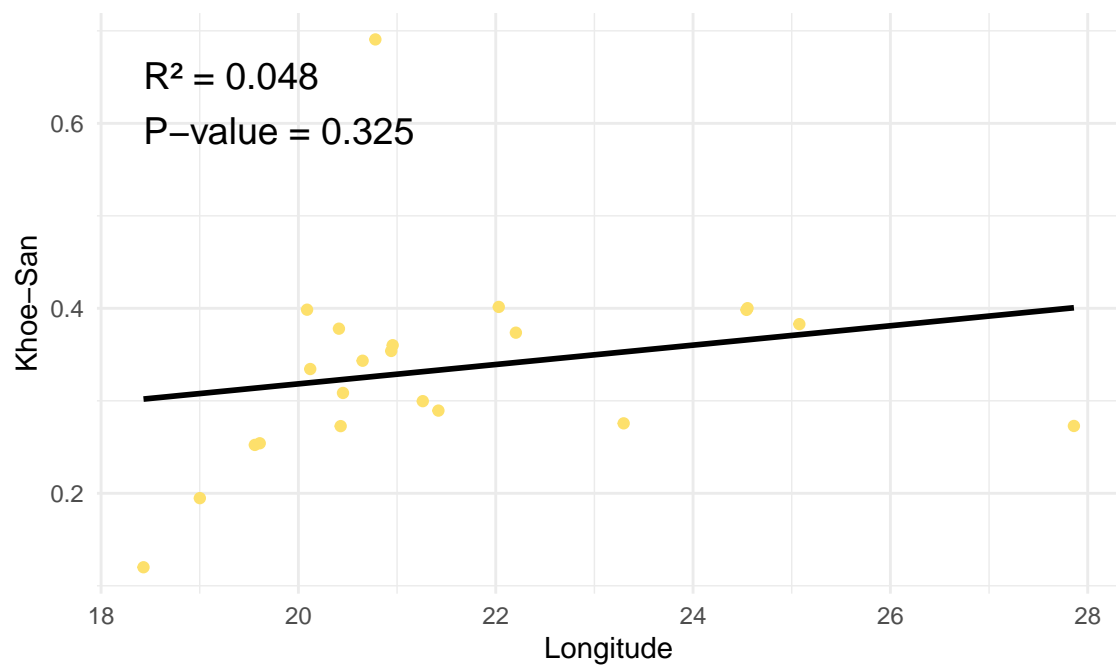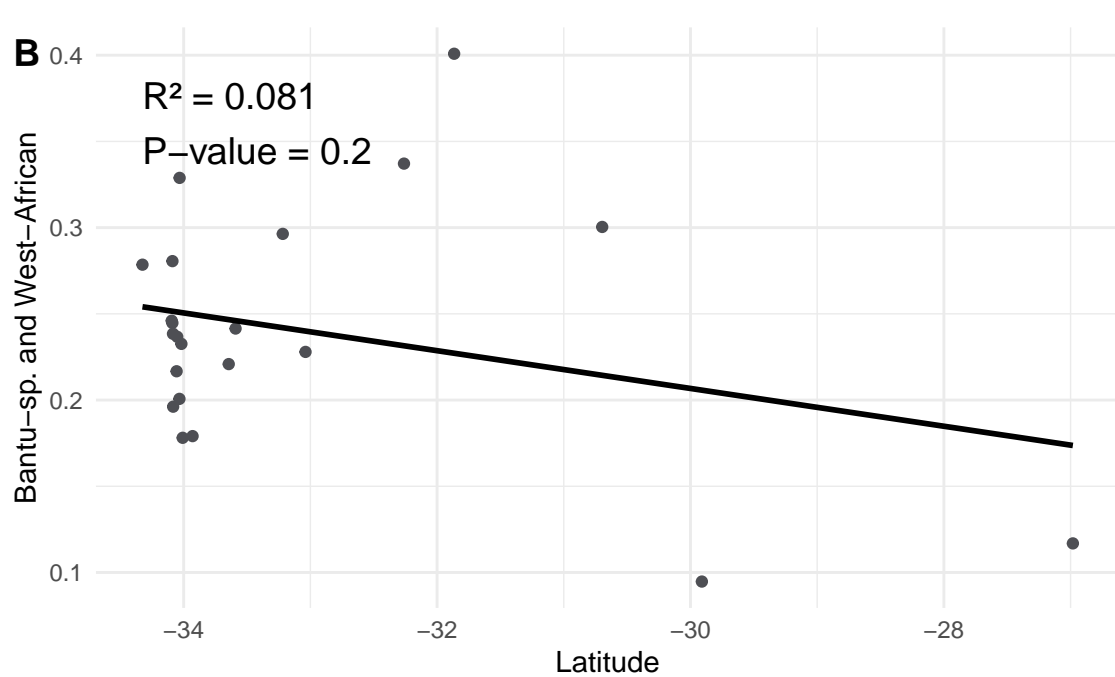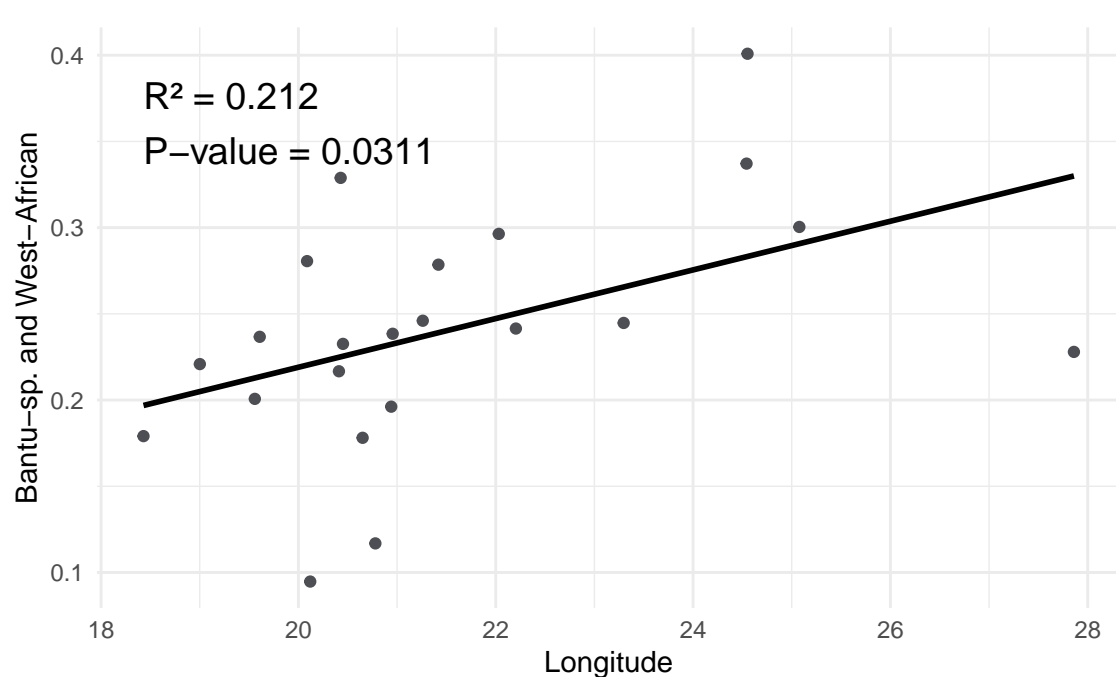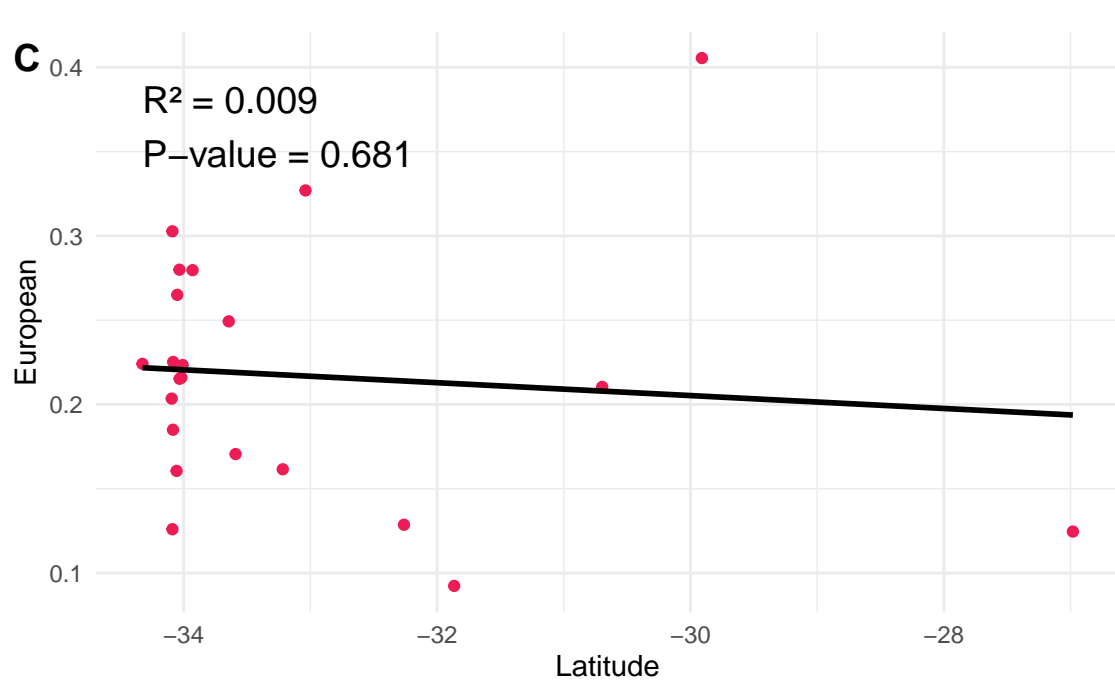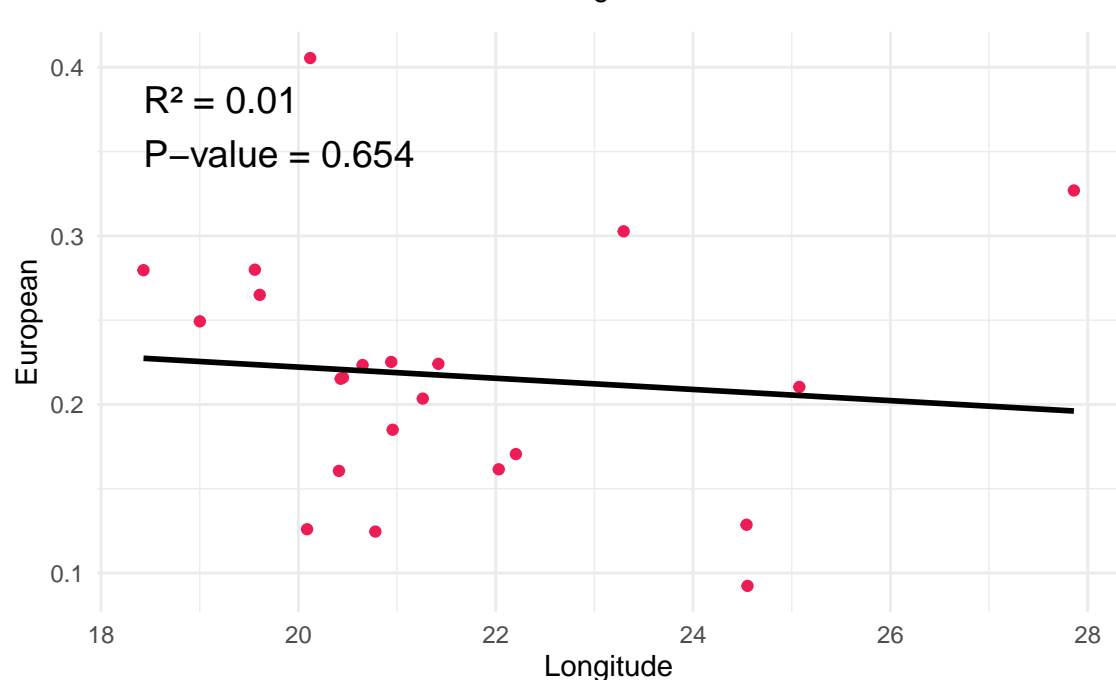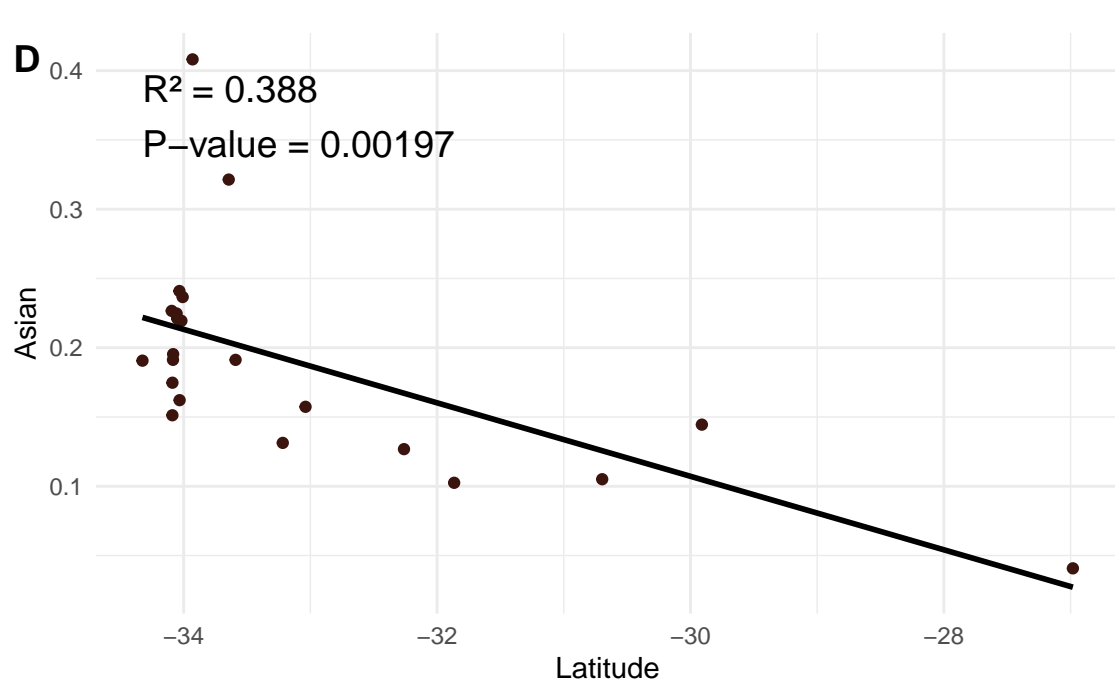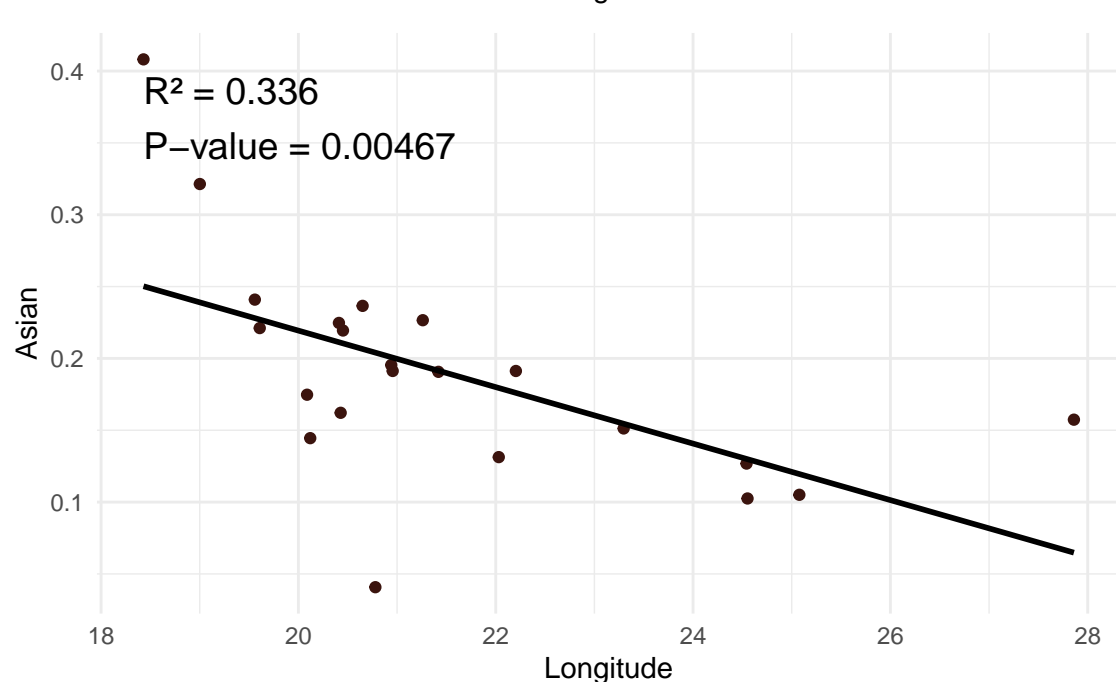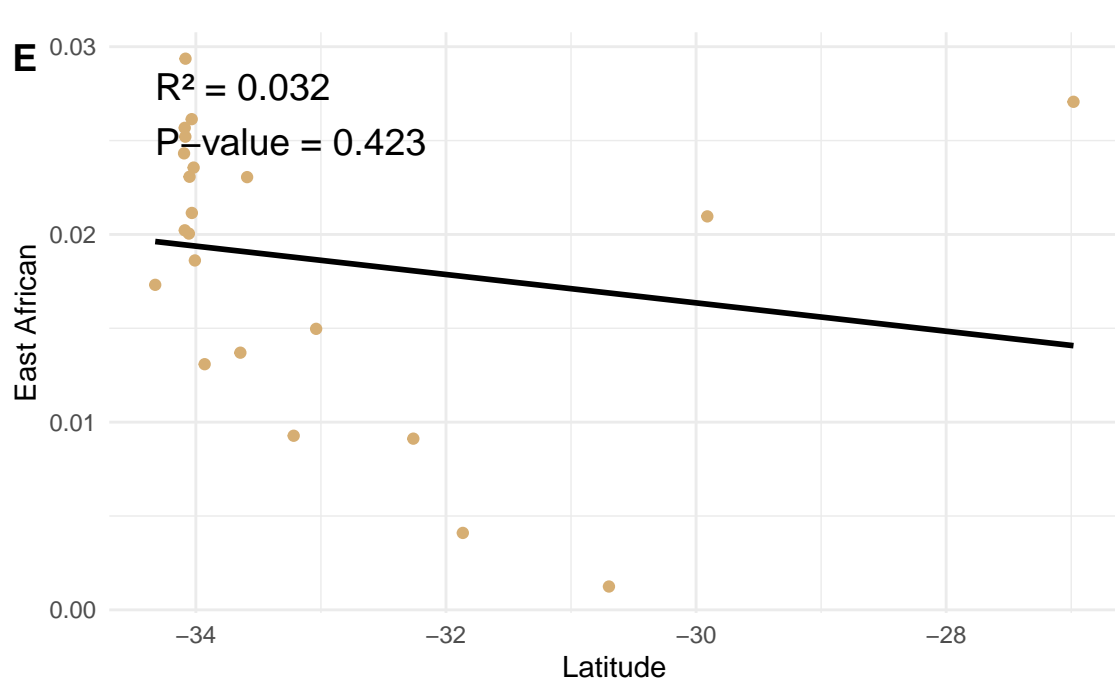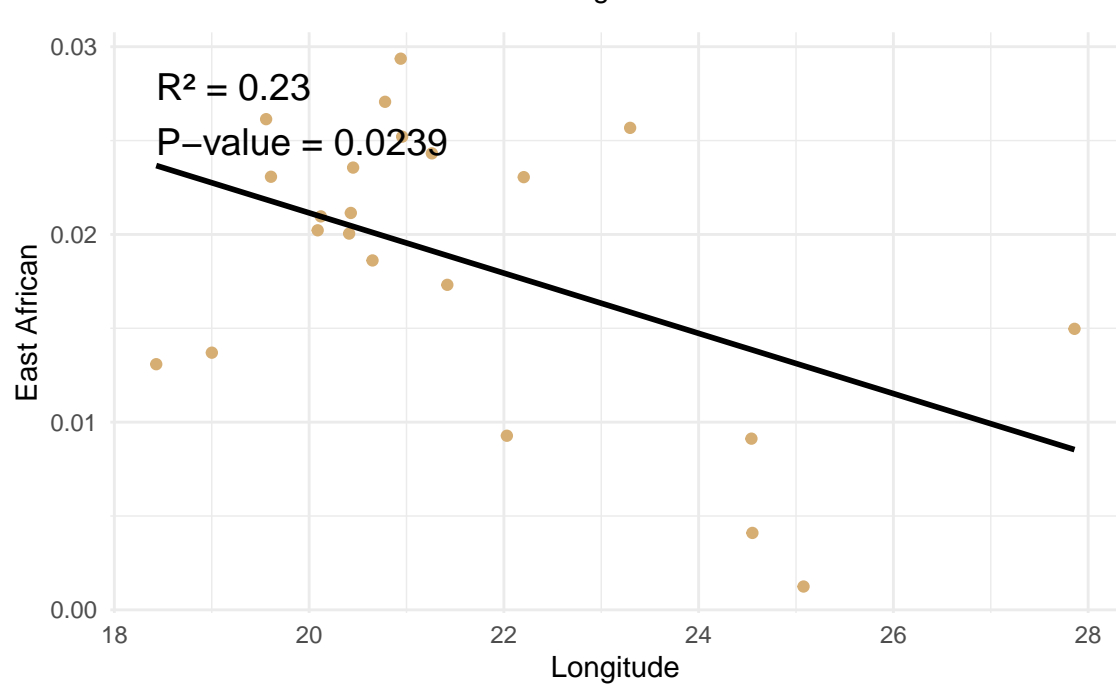

Supplement: Supplementary file 12 — Additional file 12. Averaged ADMIXTURE derived ancestry proportions from K = 6 plotted against latitude and longitude. For each ancestry, the average ancestry proportion as reported by ADMIXTURE was calculated per site and then plotted against latitude (left panels) and longitude (right panels). A linear model was fitted through the data and the $R^2$R2 is indicated, indicating how well the model fits the variance in the data. South and East Asian ancestries were combined in D). [file 12915_2025_2317_MOESM12_ESM.pdf]

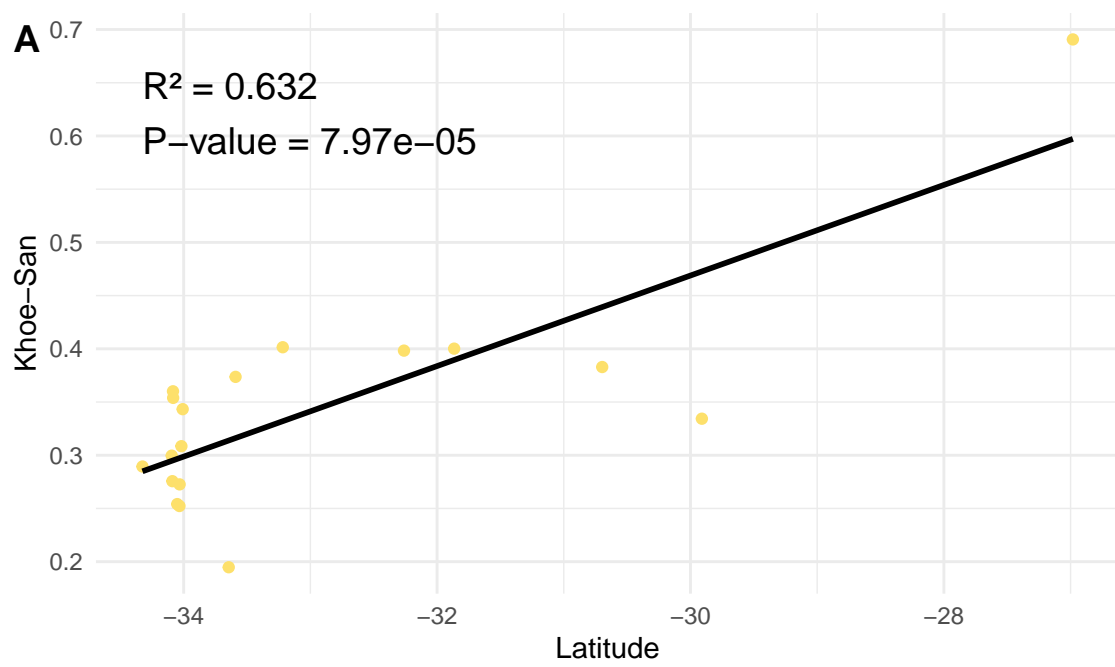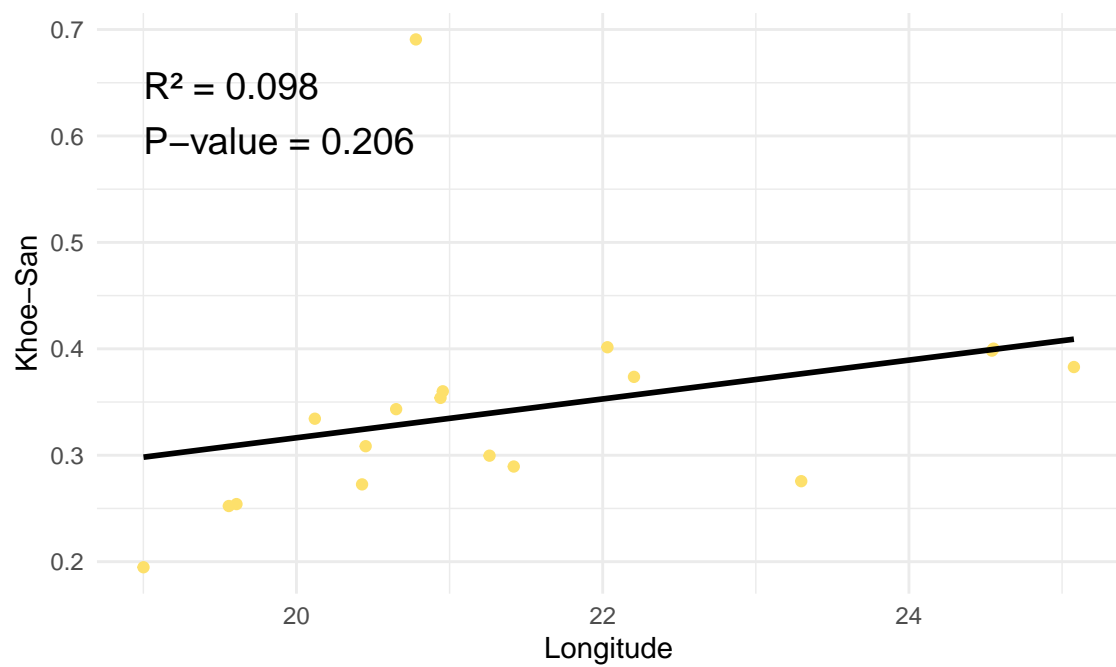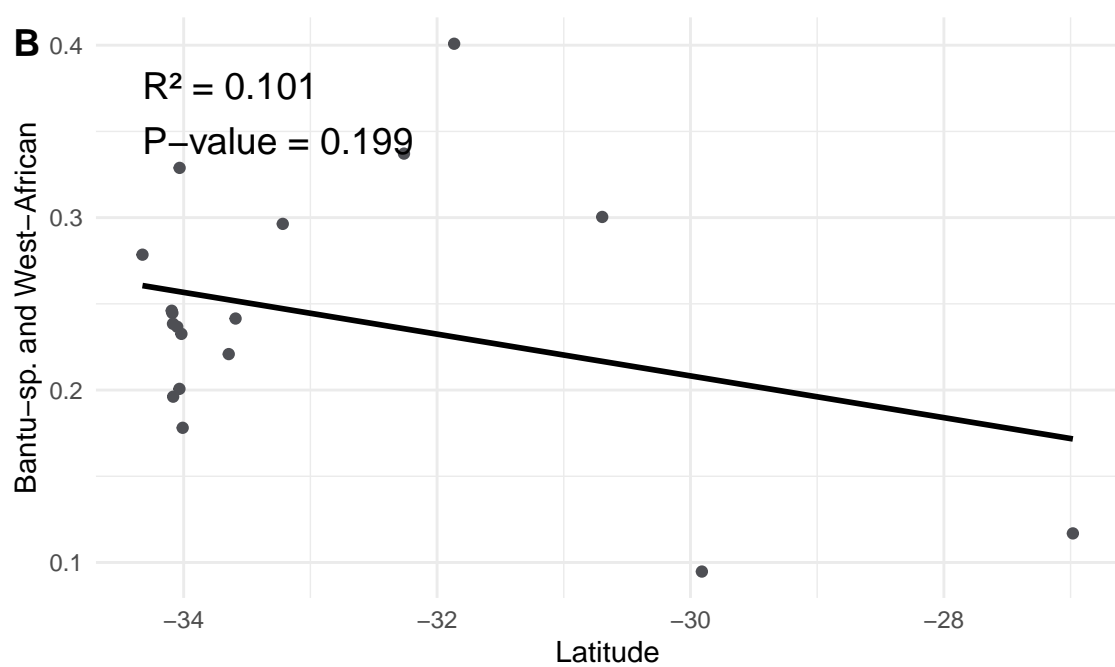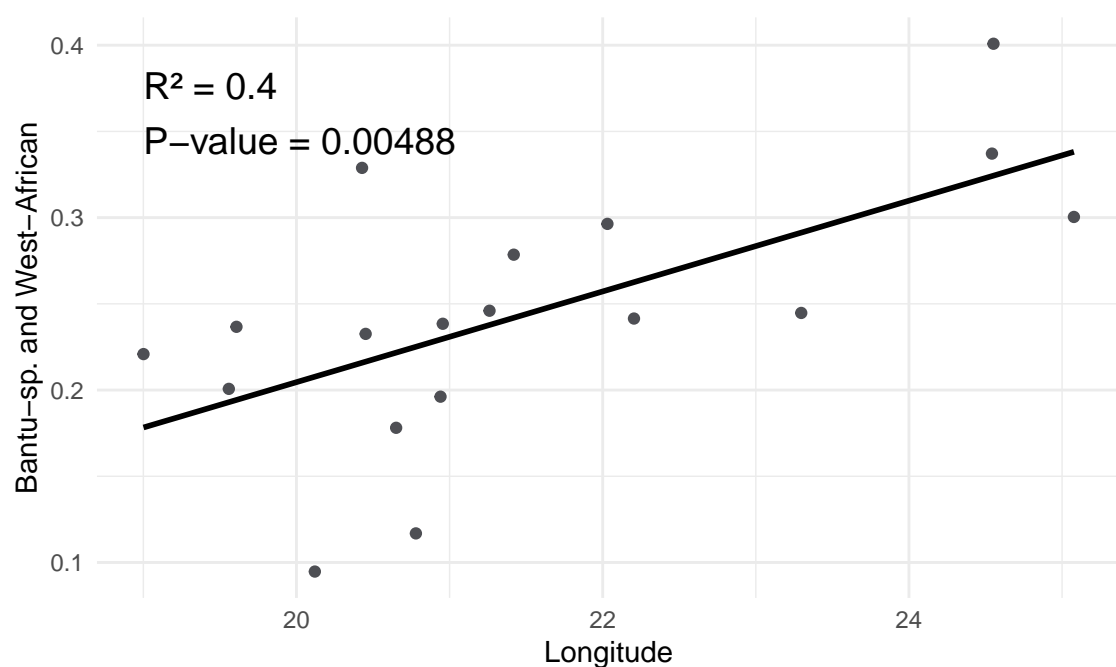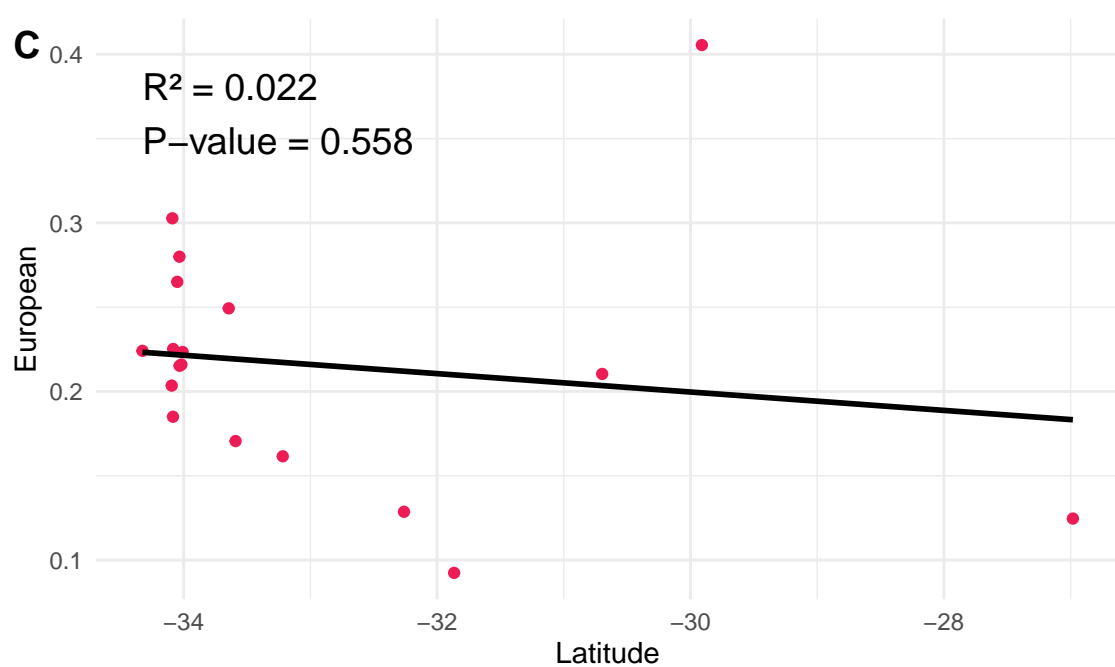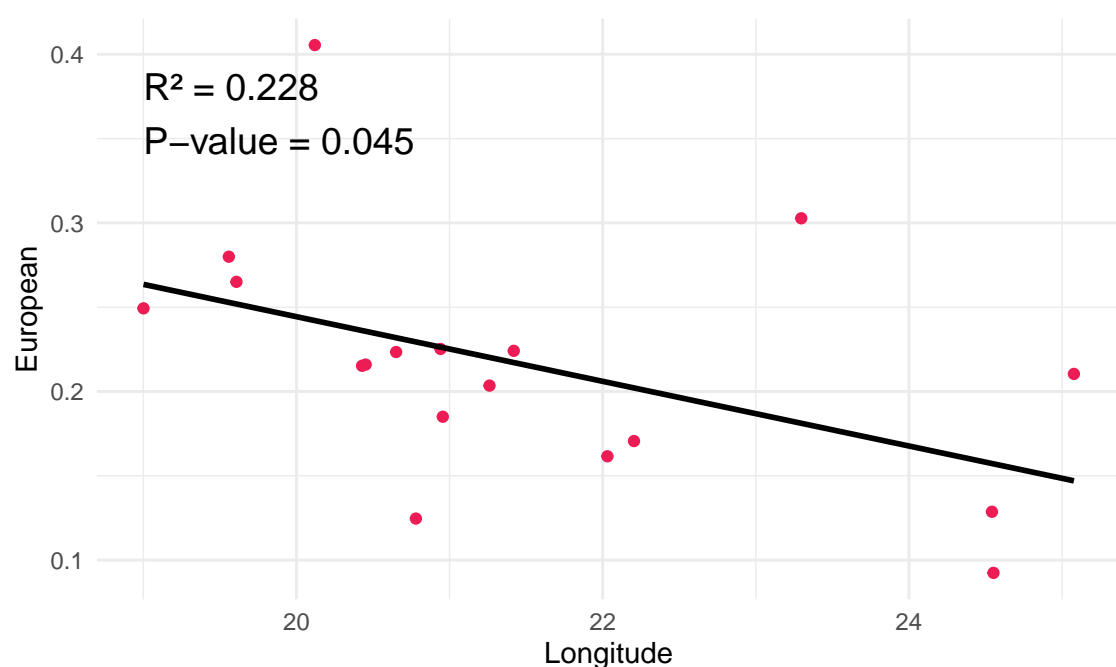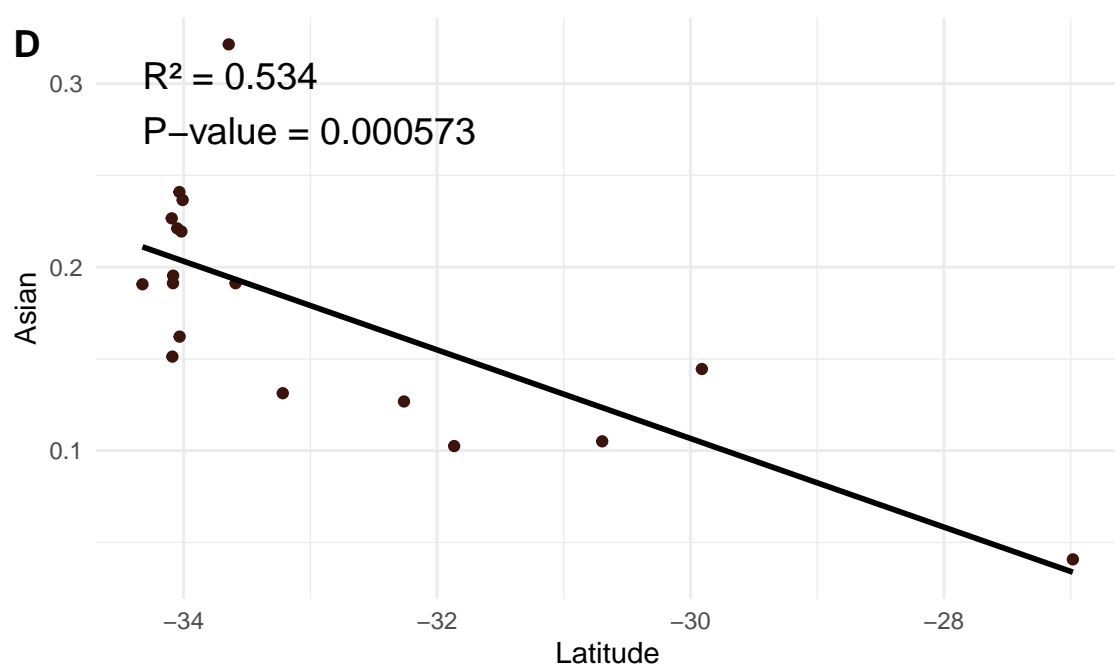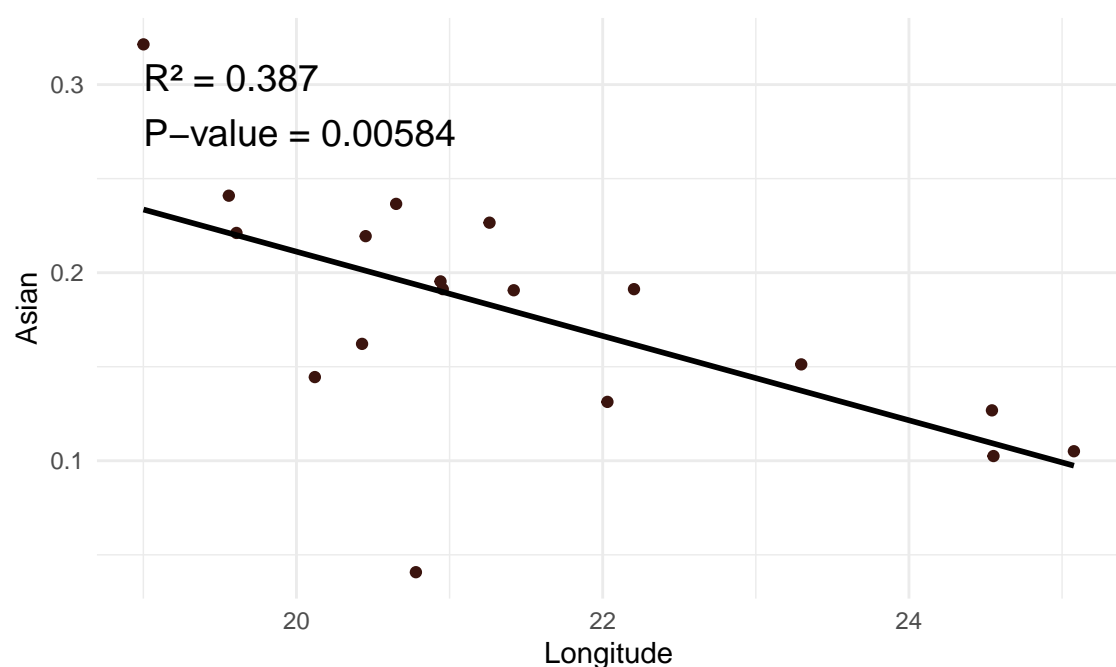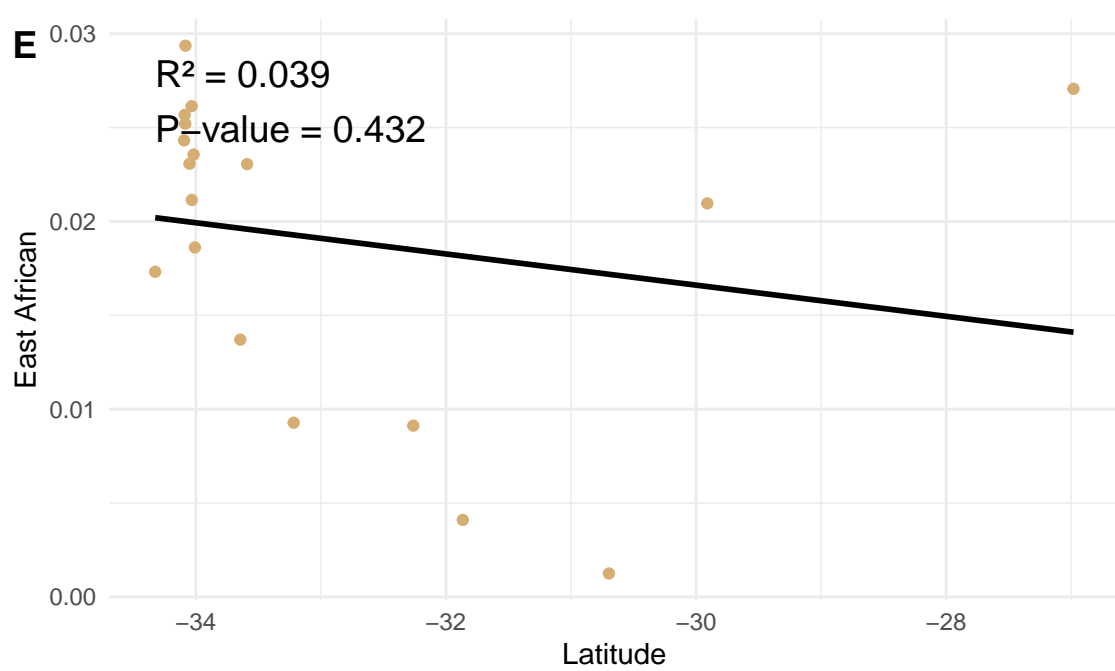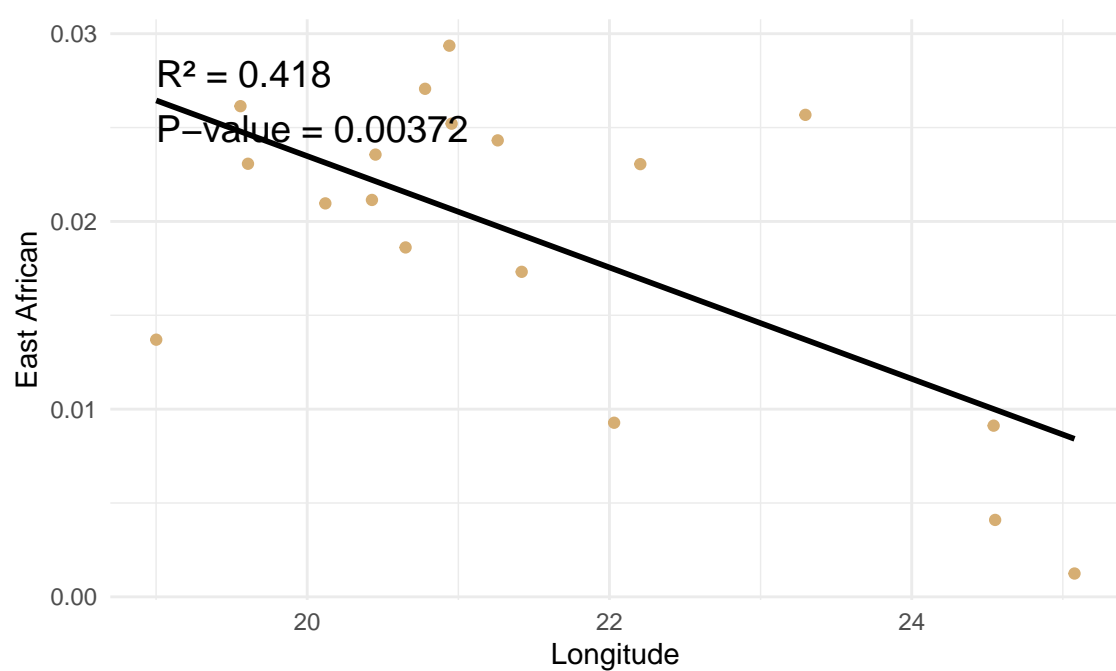

Supplement: Supplementary file 13 — Additional file 13. Averaged ADMIXTURE derived ancestry proportions from K = 6 plotted against latitude and longitude. For each ancestry, the average ancestry proportion as reported by ADMIXTURE was calculated per site and then plotted against latitude (left panels) and longitude (right panels). Sites with less than 10 individuals were removed for this analysis. A linear model was fitted through the data and the $R^2$R2 is indicated, indicating how well the model fits the variance in the data. South and East Asian ancestries were combined in D). [file 12915_2025_2317_MOESM13_ESM.pdf]

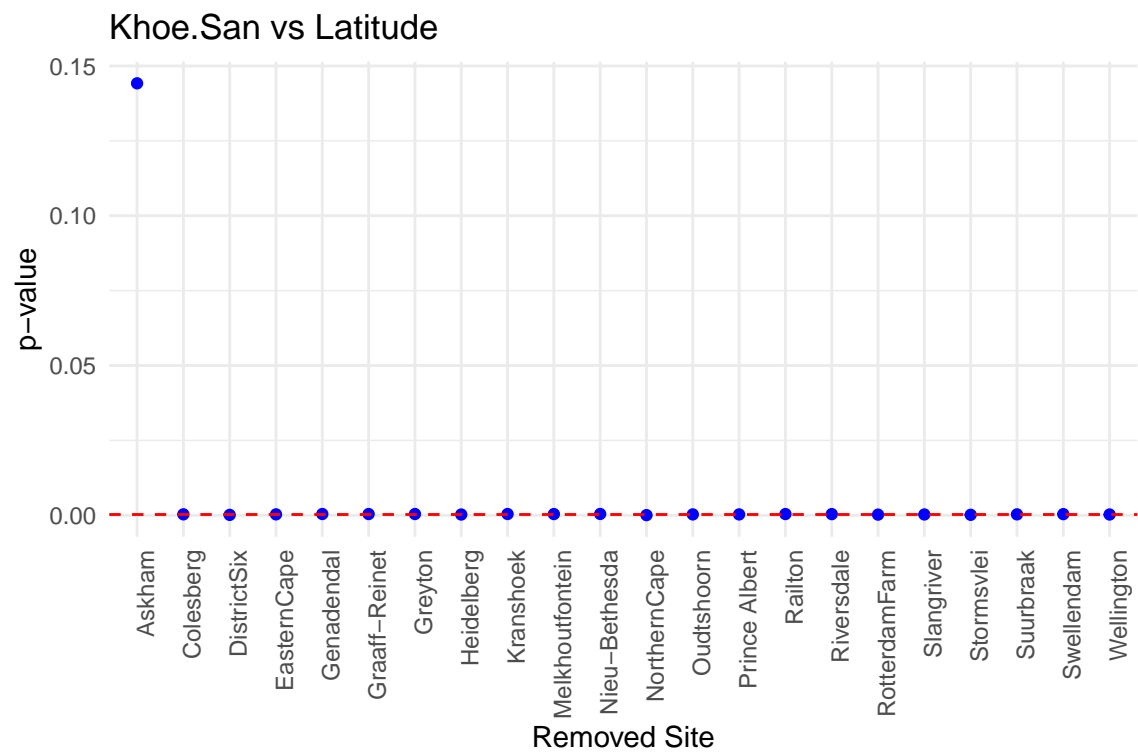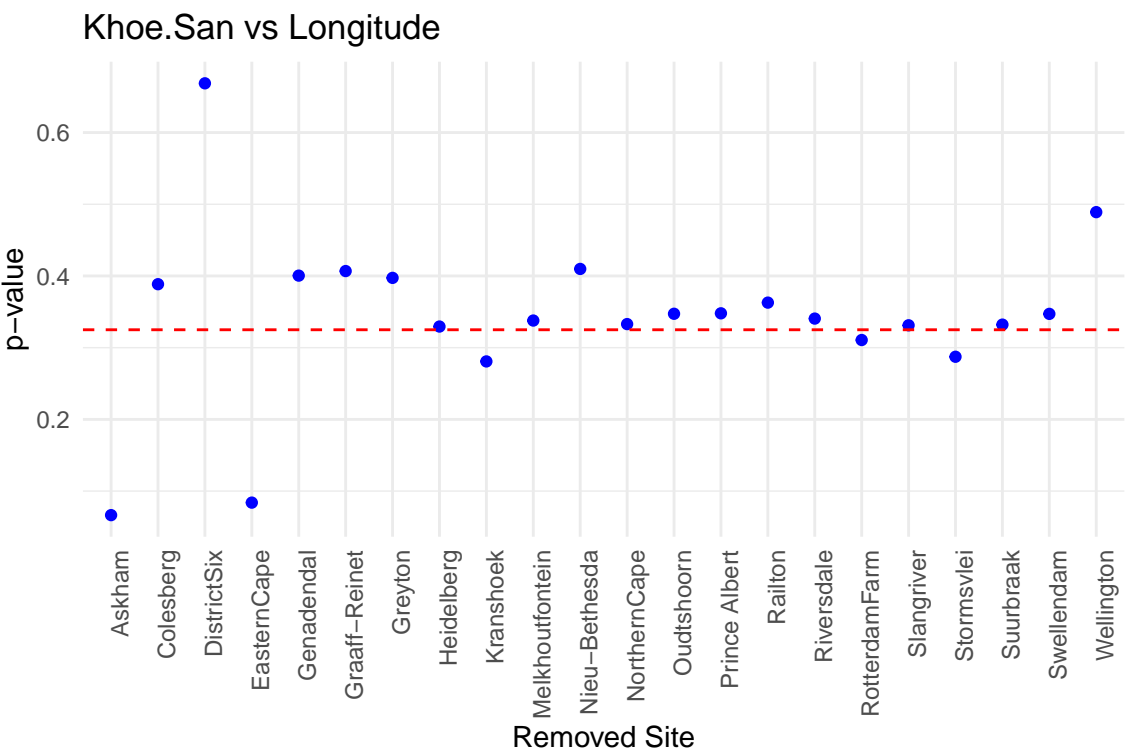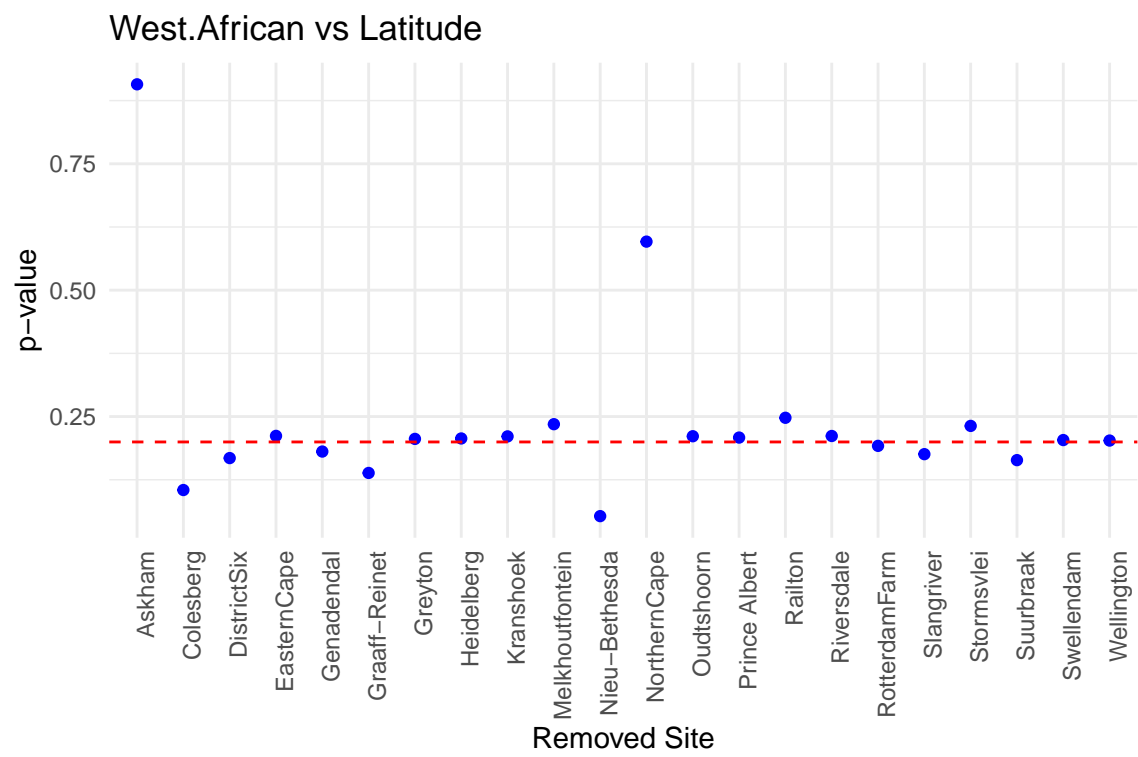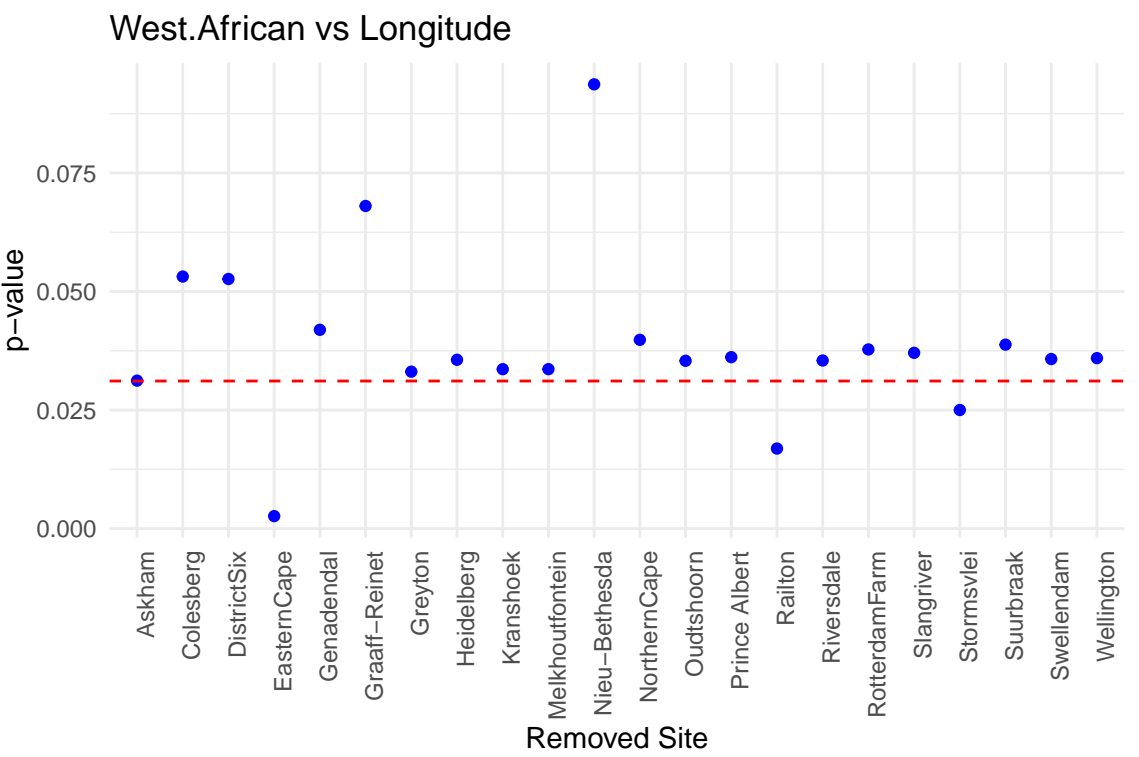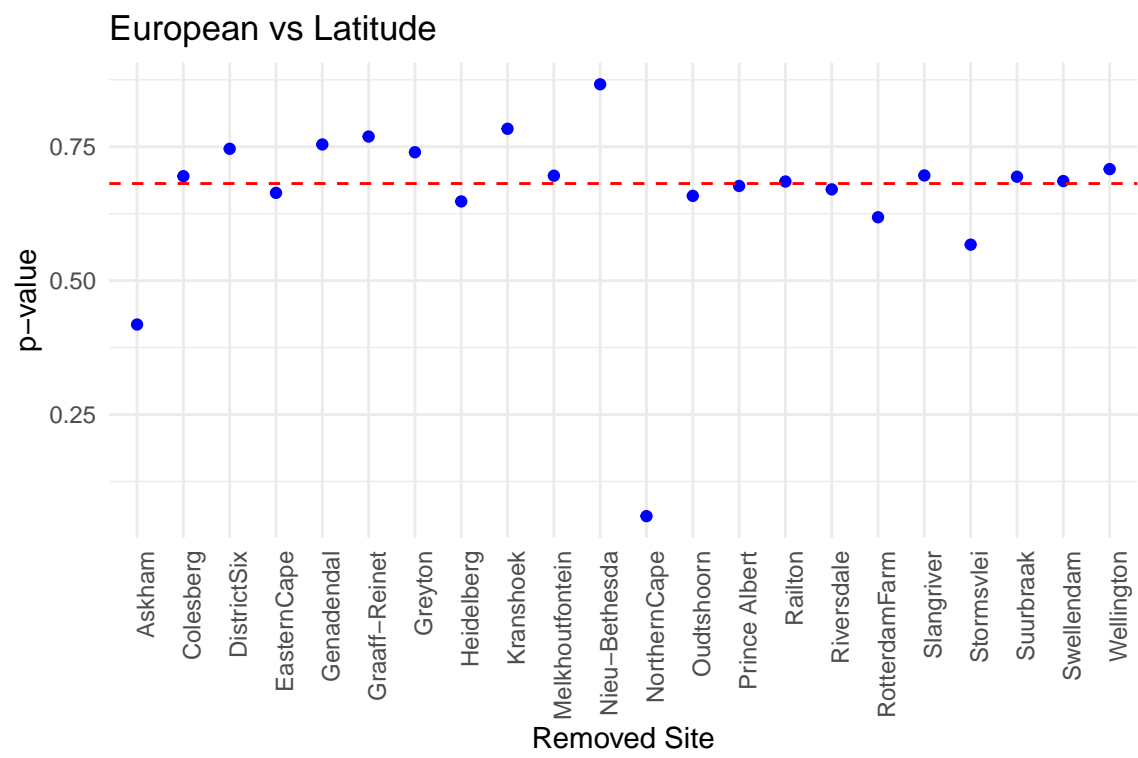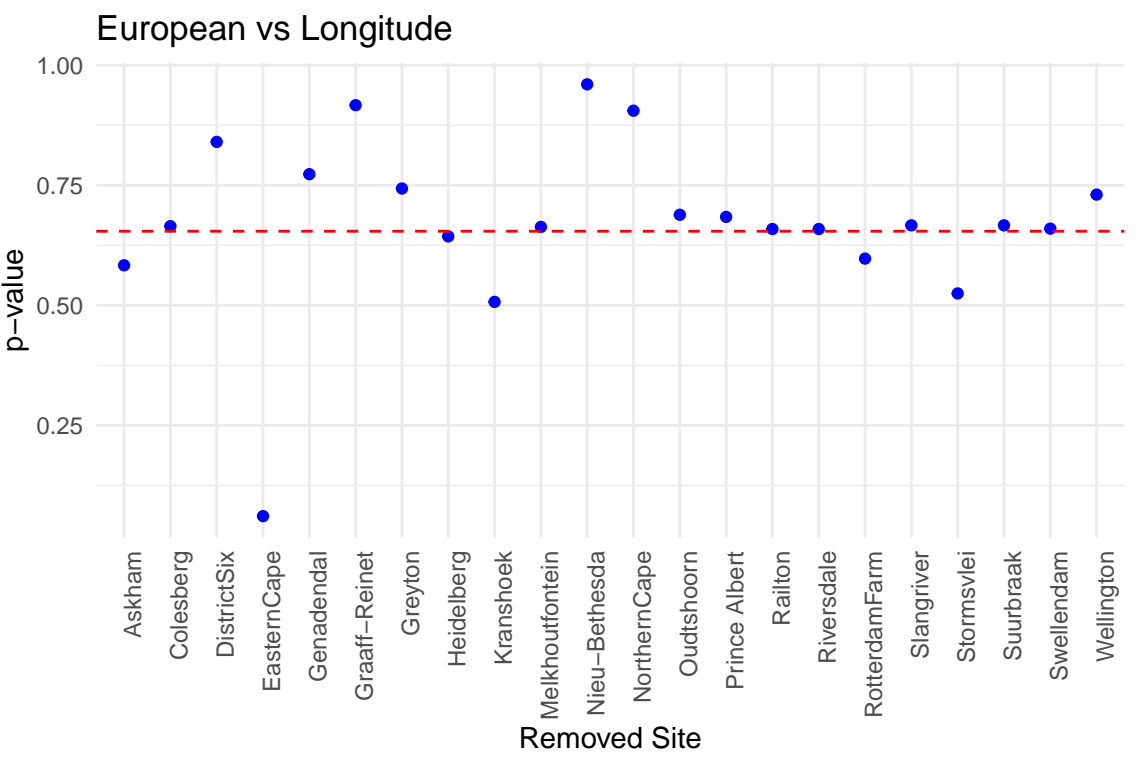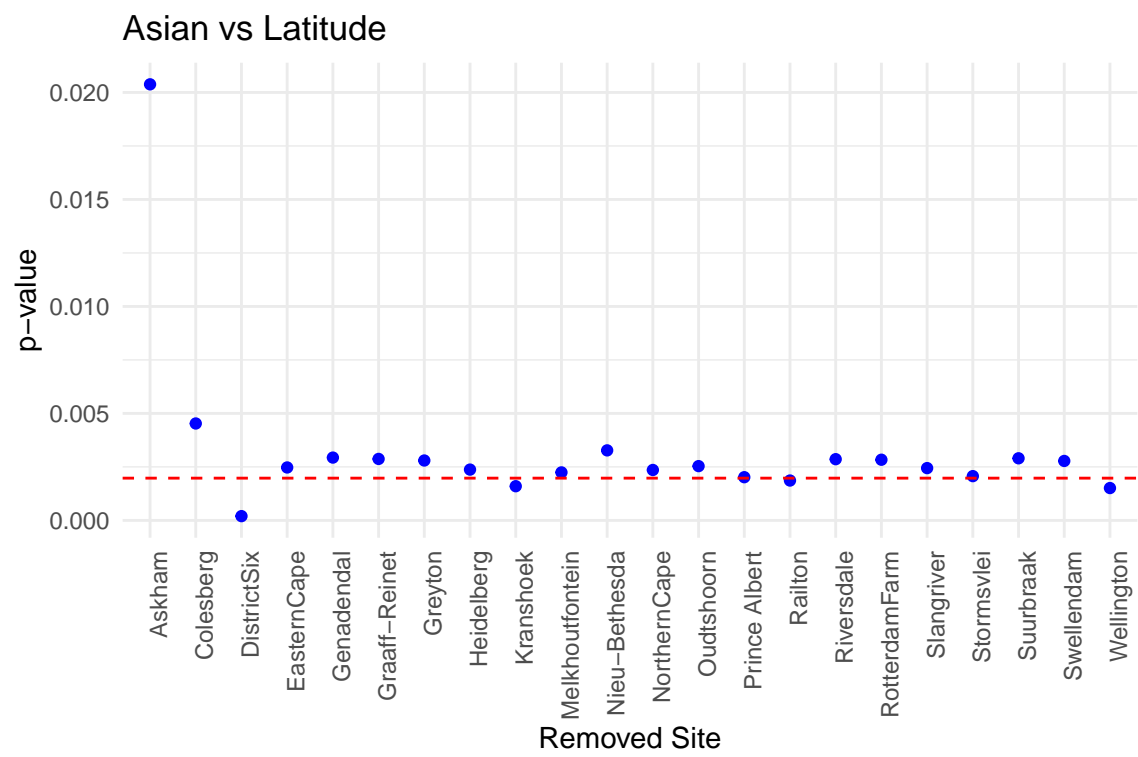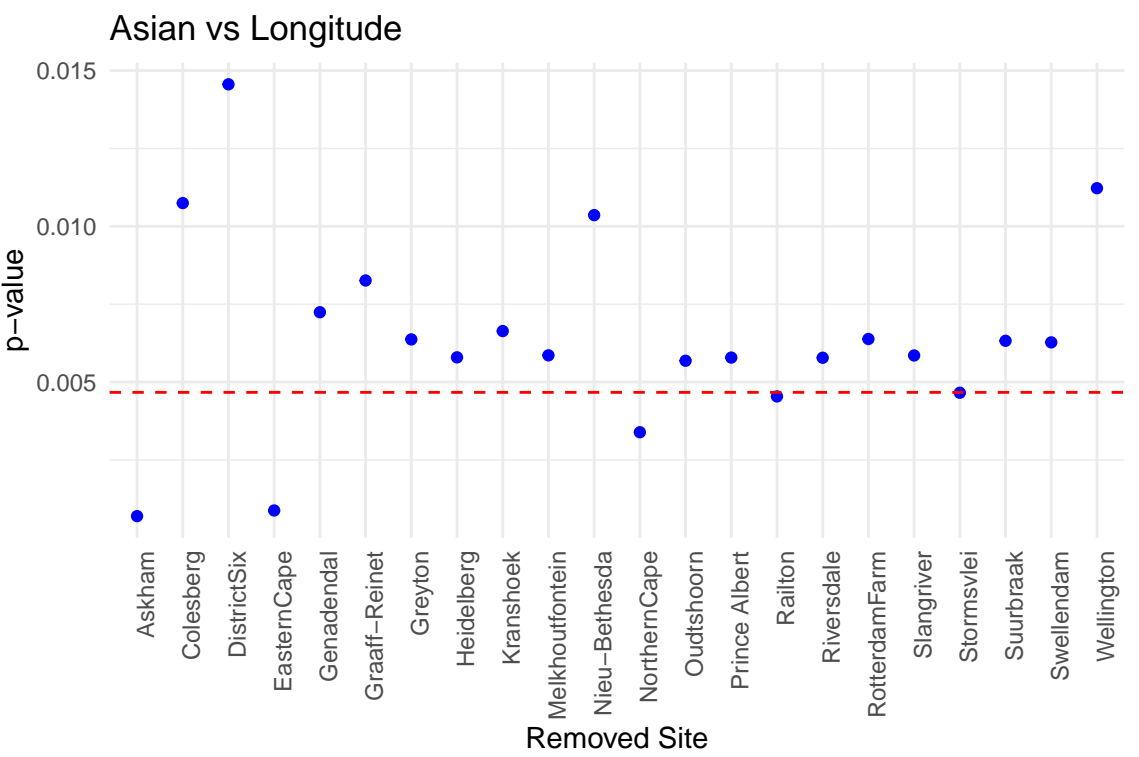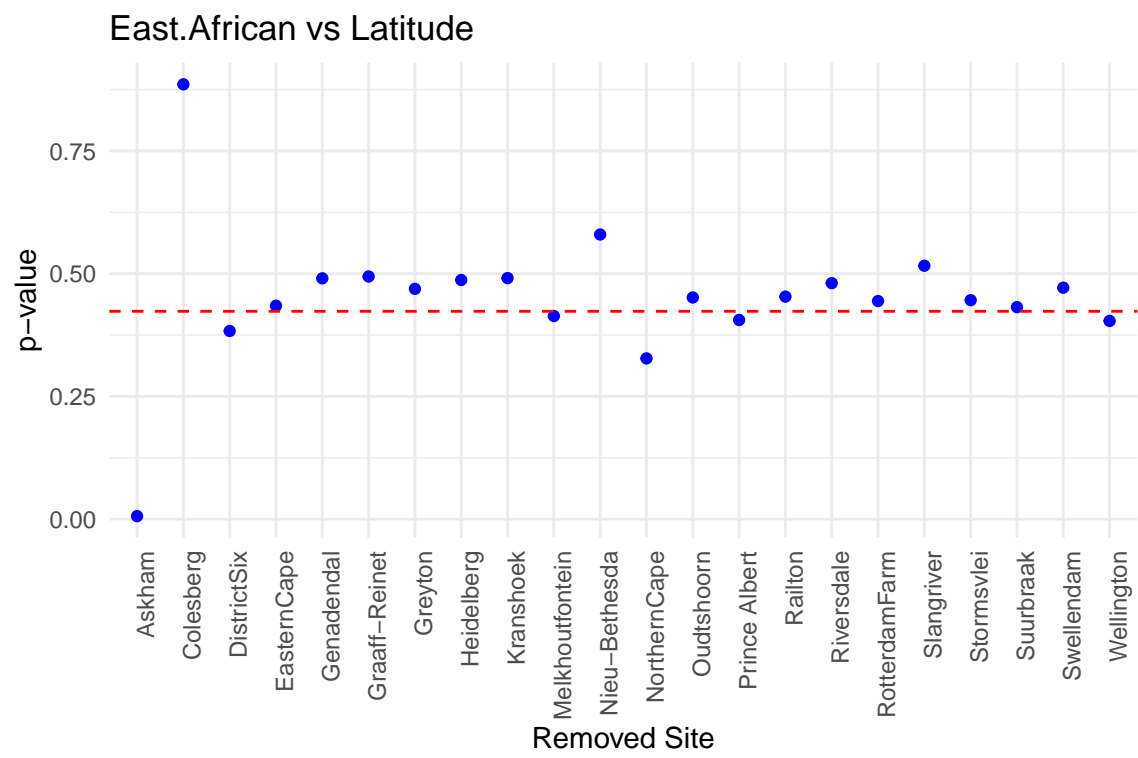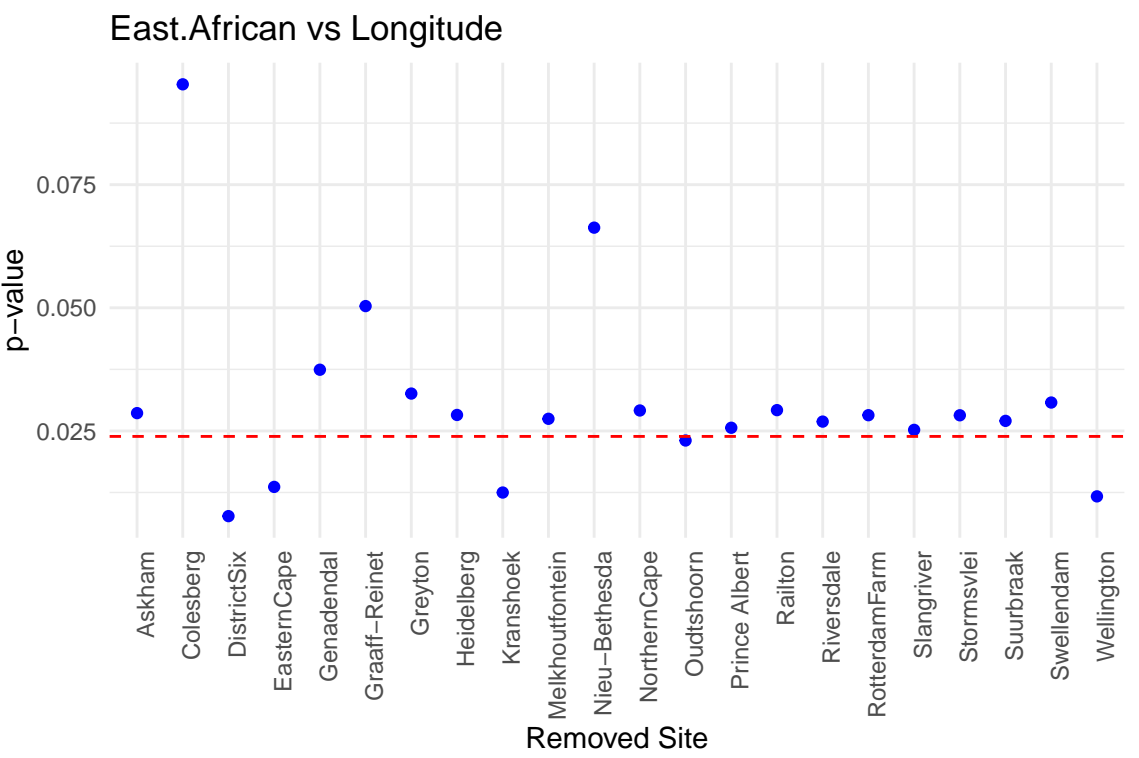

Supplement: Supplementary file 14 — Additional file 14. P-value changes in the leave-one-out analysis. This figure displays the results of a leave-one-out analysis, where the p-value for the relationship between each ancestry and latitude or longitude was recalculated after removing one site at a time. Each point represents the p-value for a model excluding a specific site, and the dashed red line indicates the p-value from the full model (using all sites). [file 12915_2025_2317_MOESM14_ESM.pdf]

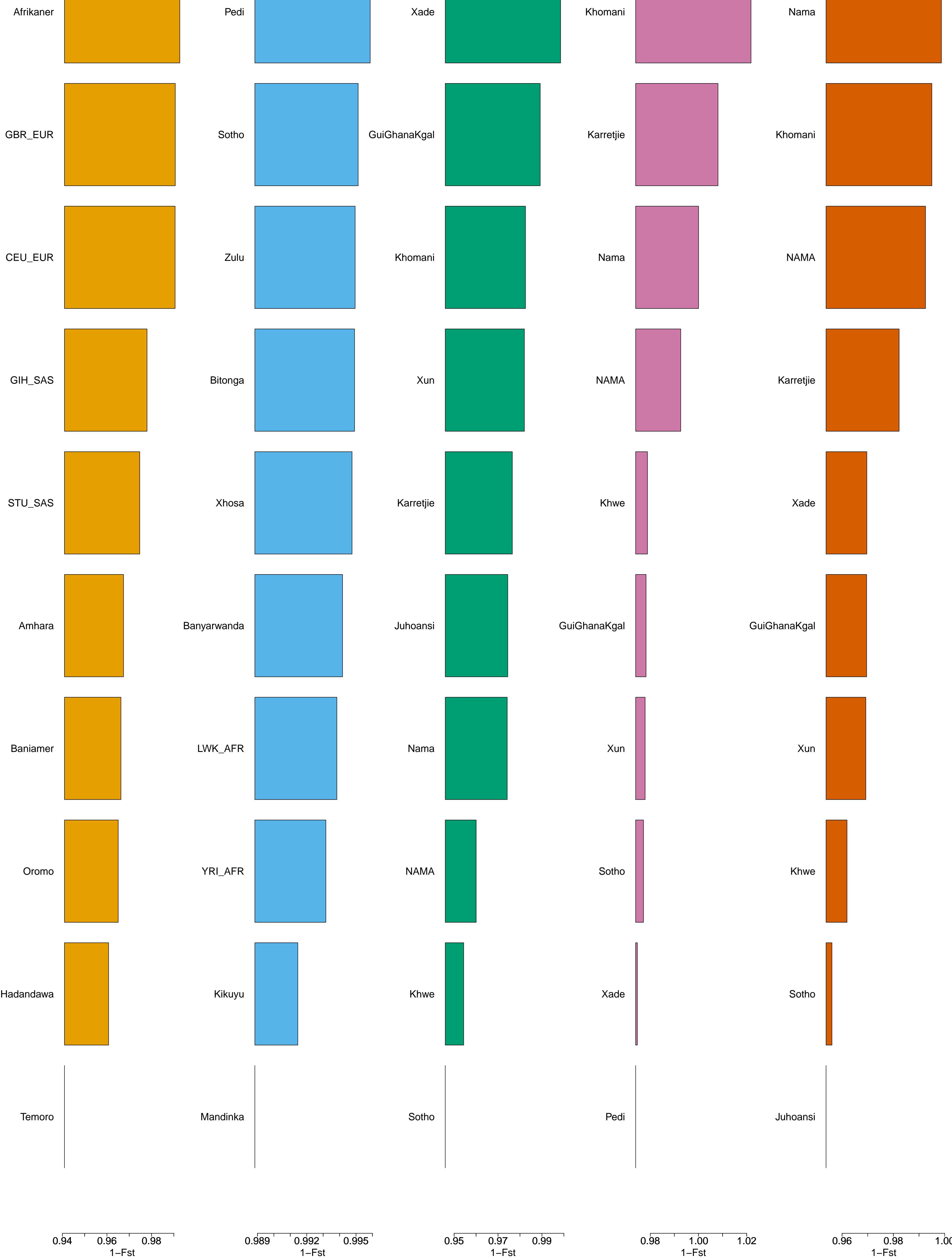

Supplement: Supplementary file 15 — Additional file 15. 1-Fst values from MOSAIC for the 5 constructed ancestries for Askham. [file 12915_2025_2317_MOESM15_ESM.pdf]

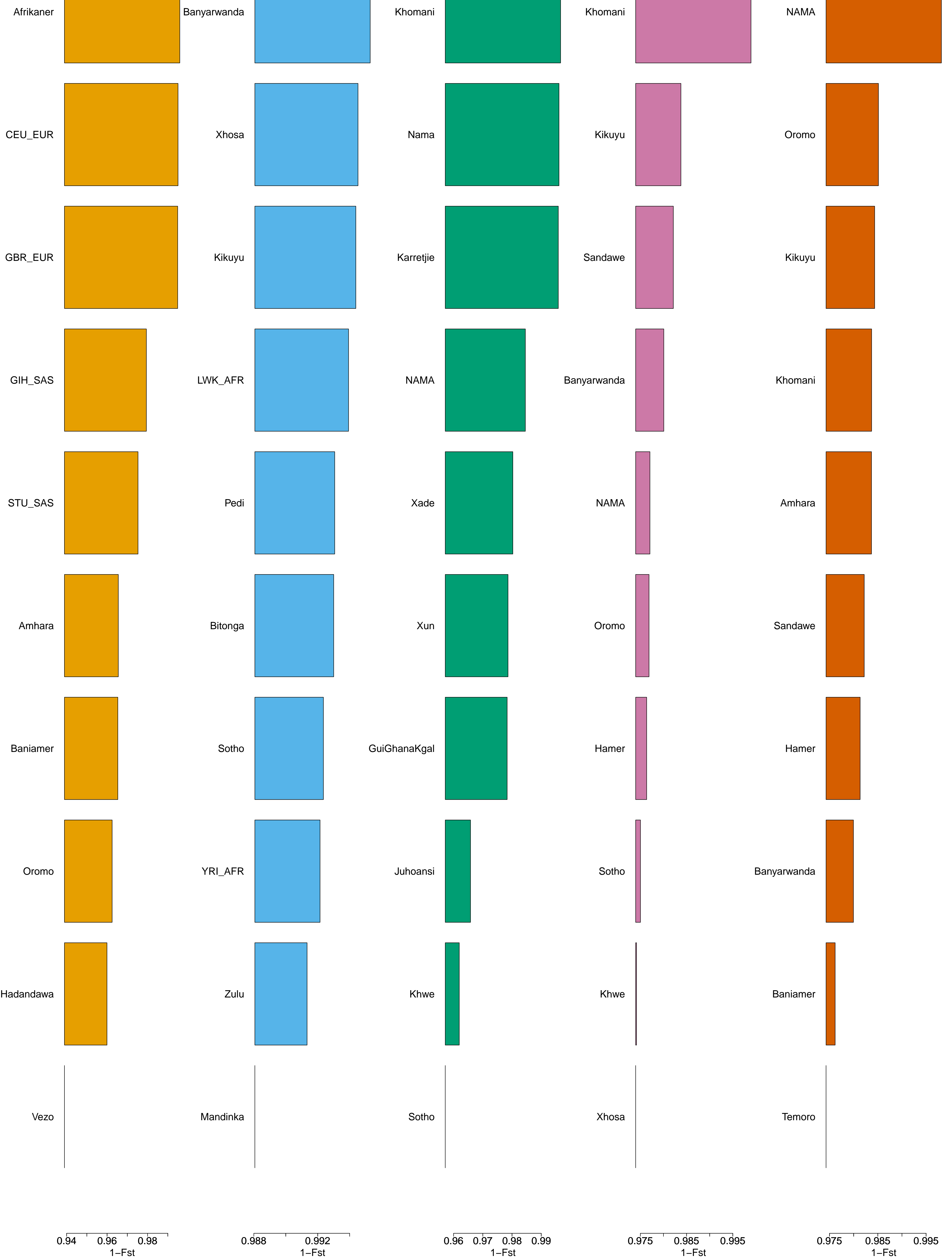

Supplement: Supplementary file 16 — Additional file 16. 1-Fst values from MOSAIC for the 5 constructed ancestries for Coloured Northern Cape. [file 12915_2025_2317_MOESM16_ESM.pdf]

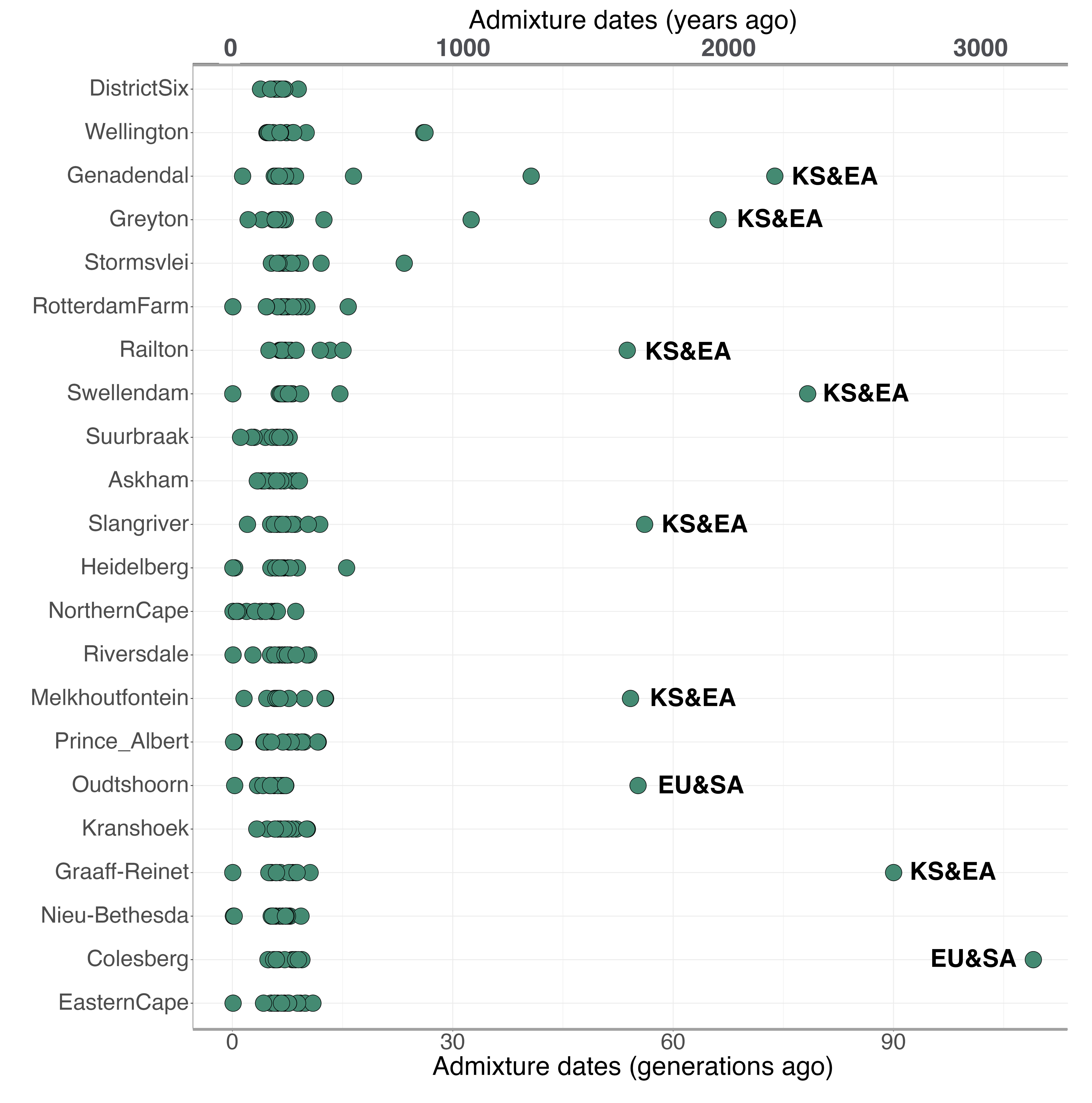

Supplement: Supplementary file 17 — Additional file 17. Inferred admixture dates (MOSAIC) for the 5-way admixture scenario for the 22 SAC populations using all reference populations as putative sources. Dots labeled with “EA & KS” indicate admixture events between Khoe-San and East African constructed ancestries, as determined by Fst. Dots labeled with “EU & SA” indicate admixture events between European and South Asian constructed ancestries. Sites are shown from West (high) to East (low) on the y-axis. X-axis on top shows the time in years, x-axis at the bottom shows time in generations ago. [file 12915_2025_2317_MOESM17_ESM.jpeg]

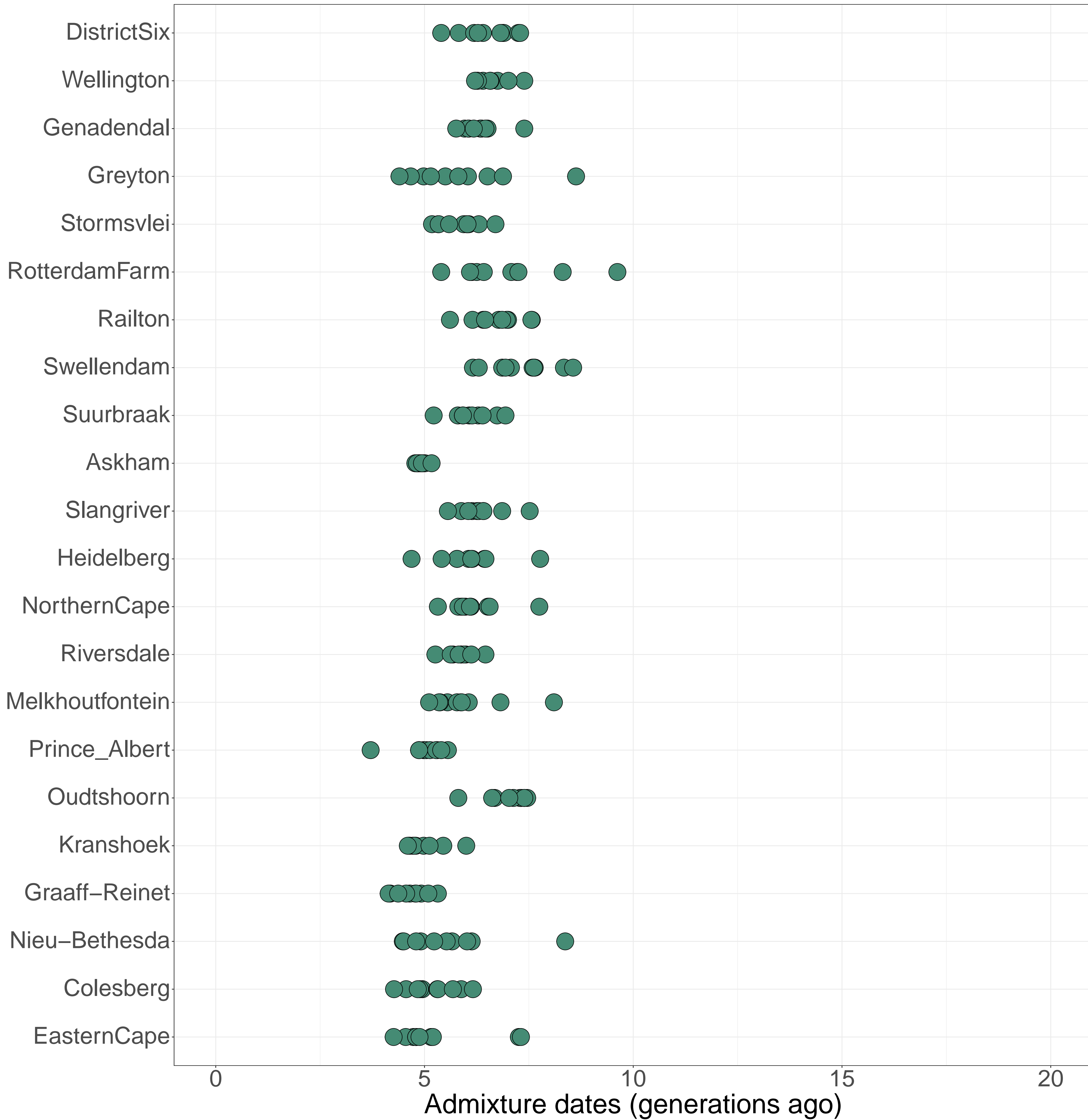

Supplement: Supplementary file 18 — Additional file 18. Inferred admixture dates (MALDER) for the 5-way admixture scenario for the SAC populations using Juhoansi, YRI_AFR, Amhara, STU_SAS, and CEU_EUR as putative sources. Sites are shown from West (high) to East (low) on the y-axis. X-axis shows time in generations ago. [file 12915_2025_2317_MOESM18_ESM.pdf]

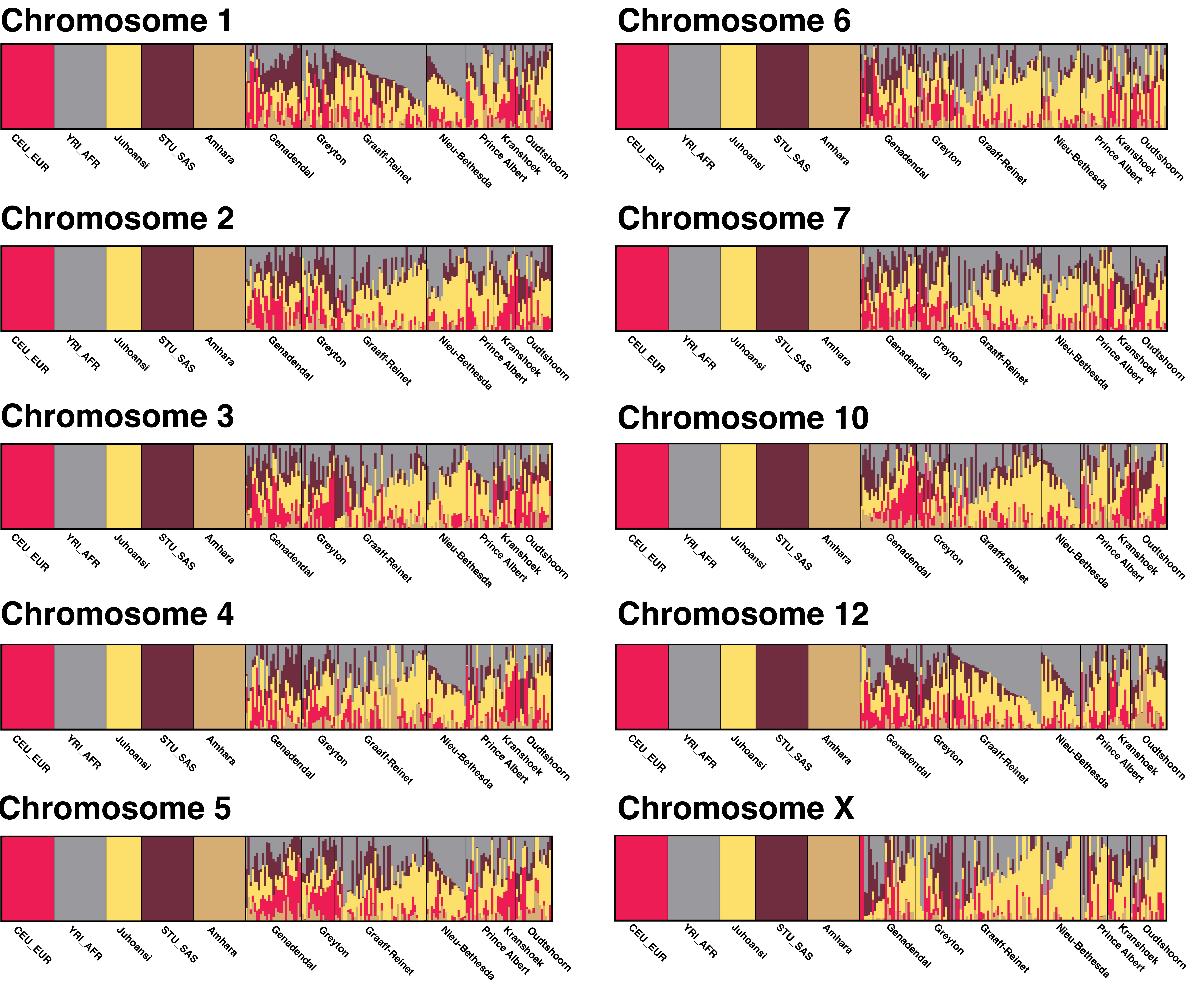

Supplement: Supplementary file 19 — Additional file 19. Supervised ADMIXTURE results per chromosome, visualized using PONG for K = 5. These ADMIXTURE results were used to calculate the $\Delta$ΔAdmix ratios depicted in Figure 3. Each ADMIXTURE run is based on 13,000 SNPs. [file 12915_2025_2317_MOESM19_ESM.jpeg]

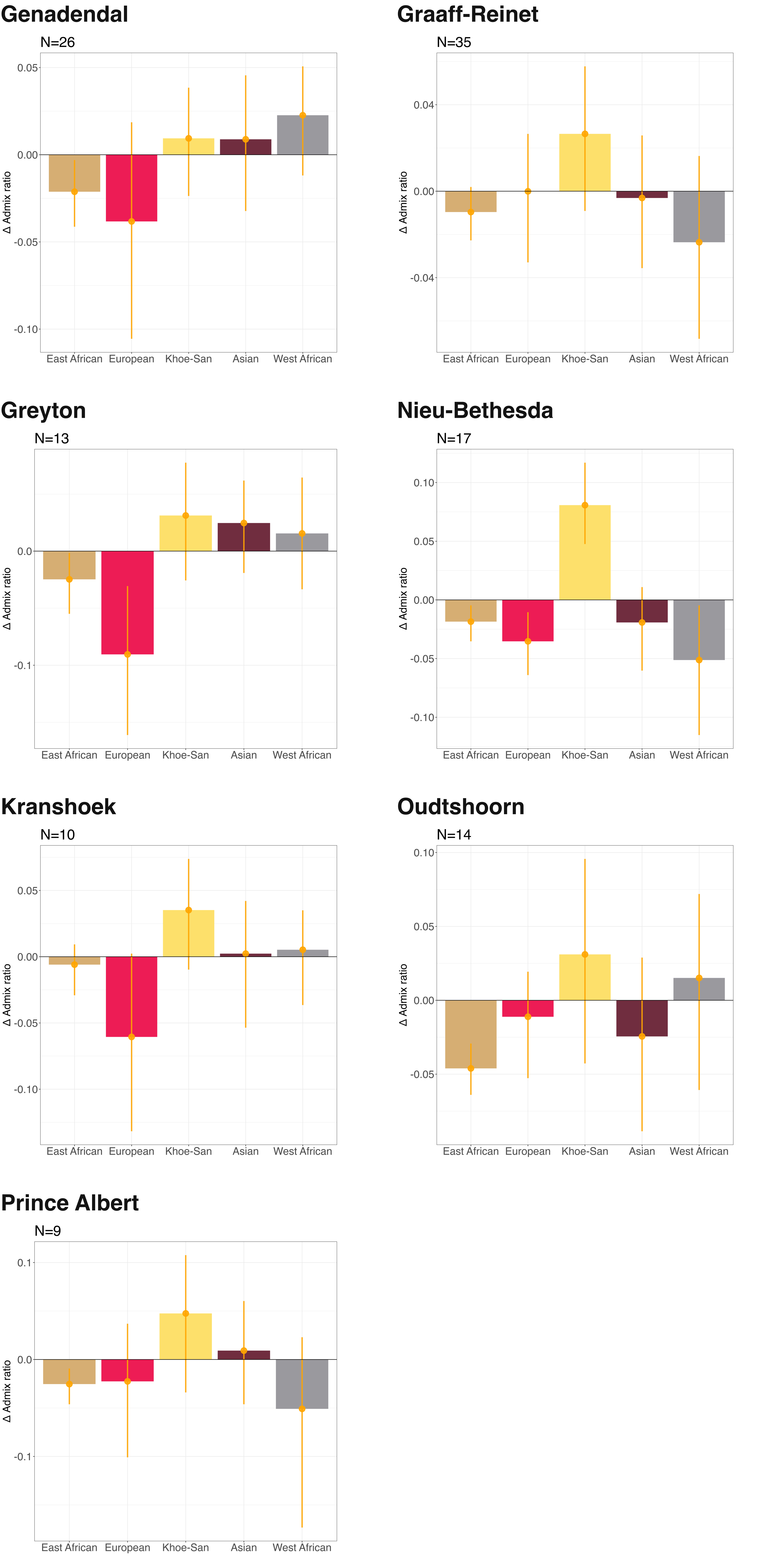

Supplement: Supplementary file 20 — Additional file 20. $\Delta$ΔAdmix ratios for each of the seven ancestries, shown separately for the seven investigated sites. X and autosomal proportions were bootstrapped (10,000 times) and average X-to-autosomal difference ratios were calculated for each of the five ancestries, as well as standard deviations. The error bars indicate the 95% confidence interval. Negative X-to-autosomal difference ratios are indicative of male-biased admixture for that ancestry, positive X-to-autosomal difference ratios are indicative of a female-biased admixture for that ancestry. [file 12915_2025_2317_MOESM20_ESM.jpeg]

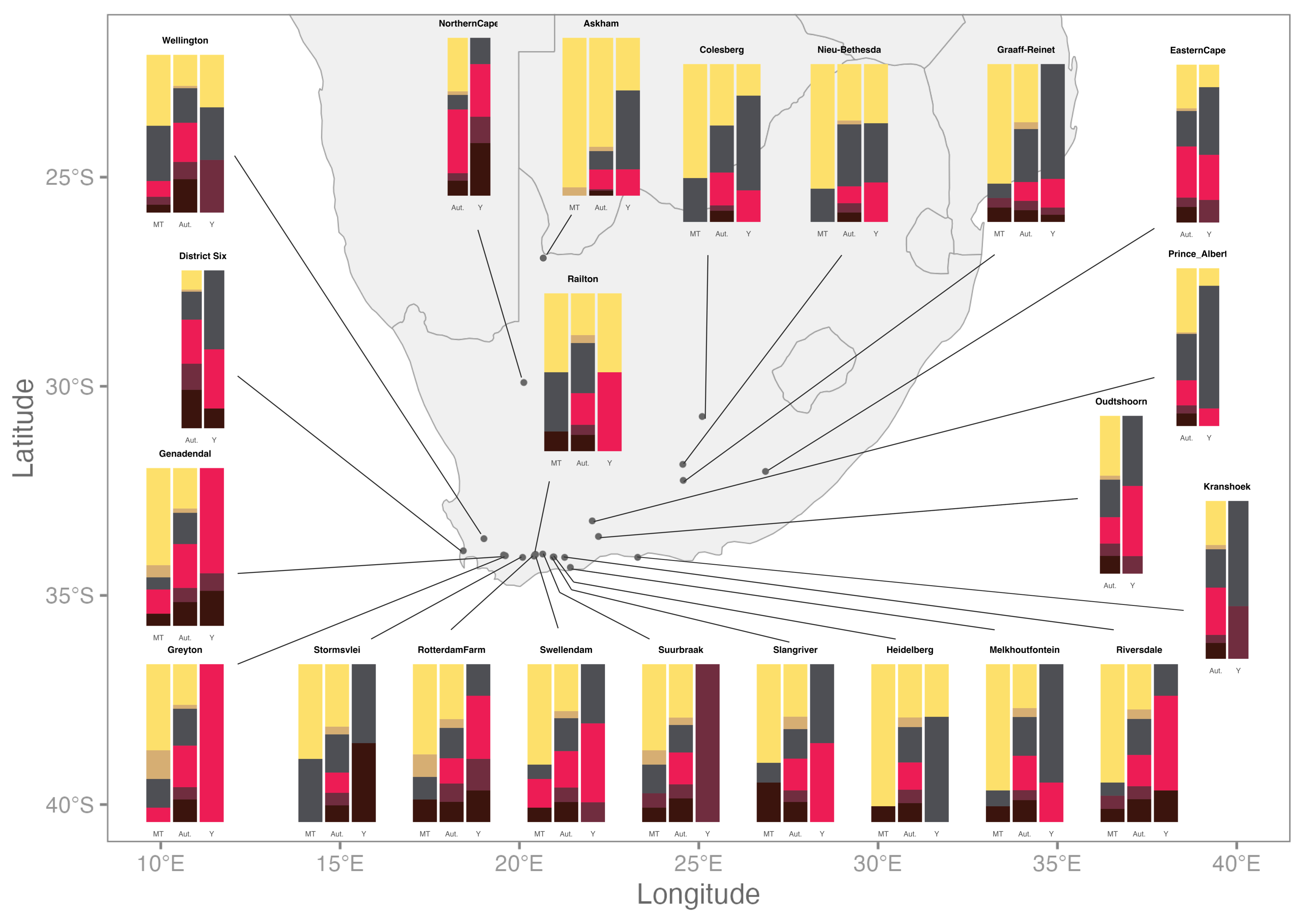

Supplement: Supplementary file 21 — Additional file 21. Maternal, autosomal, and paternal ancestries at each location with SAC individuals. Where no information about maternal or paternal ancestries was available, these are not shown. [file 12915_2025_2317_MOESM21_ESM.jpeg]
