# Supplementary material for: Wide-scale geographical analysis of genetic ancestry in the South African Coloured population
Source: BMC Biol. 2025 Jul 22;23:219. doi: 10.1186/s12915-025-02317-5 (PMC12281806; doi:10.1186/s12915-025-02317-5)
Supplement: Supplementary file 22 — Additional file 22: Table S1. Admixture proportions at K = 6 for the 22 SAC populations. Newly investigated sites are denoted in bold. Table S2. Admixture proportions at K = 10 for the 22 SAC populations. Newly investigated sites are denoted in bold. Table S3. Mitochondrial ancestries at the 16 sites for which mitochondrial sequences were available. Newly investigated sites are denoted in bold. Table S4. Y chromosome ancestries at the 22 sites. Newly investigated sites are denoted in bold. Table S5. Number of individuals used at each site to make inferences about mitochondrial, autosomal and Y chromosomal ancestries. Table S6. List of populations included in the dataset. Table S7. Expected r-squared values for the local ancestry inference from MOSAIC for each of the 22 sites. Table S8. Mitochondrial DNA haplogroup assignment by ancestry. Table S9. Y-chromosome haplogroup assignment by ancestry [68–90]. [file 12915_2025_2317_MOESM22_ESM.pdf]

Additional file 22: Supplementary Tables

Table S1: Admixture proportions at K = 6 for the 22 SAC populations. Newly investigated sites are denoted in bold.

Site	European	East African	East Asian	West African	Khoe-San	South Asian
Colesberg	0.210	0.001	0.033	0.300	0.382	0.071
DistrictSix	0.279	0.013	0.163	0.179	0.120	0.244
EasternCape	0.326	0.014	0.058	0.227	0.272	0.099
NorthernCape	0.405	0.020	0.046	0.094	0.334	0.097
Wellington	0.249	0.013	0.107	0.220	0.194	0.213
Askham	0.124	0.027	0.009	0.116	0.690	0.031
**Genadendal**	0.279	0.026	0.089	0.200	0.252	0.151
**Graaff-Reinet**	0.128	0.009	0.042	0.337	0.398	0.084
**Greyton**	0.265	0.023	0.076	0.236	0.254	0.144
Heidelberg	0.185	0.025	0.069	0.238	0.360	0.121
**Kranshoek**	0.302	0.025	0.049	0.244	0.275	0.101
Melkhoutfontein	0.224	0.017	0.044	0.278	0.289	0.145
**Nieu-Bethesda**	0.092	0.004	0.043	0.400	0.400	0.059
**Oudtshoorn**	0.170	0.023	0.077	0.241	0.373	0.113
**Prince Albert**	0.161	0.009	0.050	0.296	0.401	0.080
Railton	0.215	0.021	0.058	0.328	0.272	0.103
Riversdale	0.203	0.024	0.075	0.245	0.299	0.150
RotterdamFarm	0.160	0.020	0.097	0.216	0.377	0.127
Slangriver	0.225	0.029	0.063	0.196	0.353	0.131
Stormsvlei	0.125	0.020	0.058	0.280	0.398	0.115
Suurbraak	0.223	0.018	0.087	0.178	0.343	0.148
Swellendam	0.215	0.023	0.086	0.232	0.308	0.132
Minimum value	0.092	0.001	0.009	0.094	0.120	0.031
Maximum value	0.405	0.029	0.163	0.400	0.690	0.244
Average	0.217	0.018	0.067	0.240	0.334	0.121

Table S2: Admixture proportions at K = 10 for the 22 SAC populations. Newly investigated sites are denoted in bold.

Site	Bantu speaker	Sabue-related	Northern San	European European	East Asian	Hadza--related	Southern San	East African	South Asian	West African
Colesberg	0.293	0.001	0.061	0.200	0.033	0.002	0.328	0.003	0.065	0.009
DistrictSix	0.148	0.002	0.011	0.273	0.163	0.006	0.109	0.011	0.240	0.033
EasternCape	0.214	0.002	0.029	0.314	0.058	0.005	0.250	0.016	0.093	0.016
NorthernCape	0.076	0.005	0.025	0.385	0.046	0.005	0.324	0.021	0.090	0.017
Wellington	0.198	0.007	0.014	0.240	0.107	0.002	0.182	0.011	0.208	0.025
Askham	0.097	0.004	0.165	0.096	0.009	0.002	0.552	0.026	0.026	0.019
**Genadendal**	0.183	0.004	0.014	0.262	0.089	0.005	0.245	0.028	0.145	0.019
**Graaff-Reinet**	0.331	0.004	0.048	0.114	0.042	0.002	0.358	0.009	0.078	0.009
**Greyton**	0.220	0.005	0.021	0.249	0.076	0.006	0.237	0.025	0.139	0.018
Heidelberg	0.222	0.007	0.031	0.163	0.069	0.004	0.340	0.025	0.115	0.018
**Kranshoek**	0.228	0.003	0.027	0.284	0.049	0.004	0.255	0.030	0.096	0.020
Melkhoutfontein	0.260	0.008	0.023	0.210	0.044	0.004	0.271	0.014	0.140	0.020
**Nieu-Bethesda**	0.395	0.004	0.052	0.084	0.043	0.003	0.353	0.001	0.052	0.010
**Oudtshoorn**	0.224	0.006	0.038	0.149	0.077	0.004	0.346	0.024	0.108	0.019
**Prince Albert**	0.295	0.004	0.047	0.148	0.050	0.001	0.364	0.009	0.073	0.004
Railton	0.313	0.005	0.016	0.198	0.058	0.003	0.260	0.026	0.098	0.018
Riversdale	0.235	0.007	0.023	0.186	0.075	0.004	0.283	0.024	0.145	0.013
RotterdamFarm	0.208	0.005	0.023	0.140	0.097	0.006	0.367	0.019	0.120	0.010
Slangriver	0.173	0.009	0.016	0.202	0.064	0.005	0.353	0.026	0.124	0.023
Stormsvlei	0.270	0.006	0.035	0.105	0.058	0.003	0.375	0.021	0.110	0.011
Suurbraak	0.160	0.004	0.031	0.202	0.087	0.003	0.324	0.023	0.142	0.018
Swellendam	0.207	0.005	0.021	0.198	0.086	0.007	0.296	0.022	0.127	0.027
Minimum value	0.076	0.001	0.011	0.084	0.009	0.001	0.109	0.001	0.026	0.004
Maximum value	0.395	0.009	0.165	0.385	0.163	0.007	0.552	0.030	0.240	0.033
Average	0.225	0.005	0.035	0.200	0.067	0.004	0.308	0.019	0.115	0.017

Table S3: Mitochondrial ancestries at the 16 sites for which mitochondrial sequences were available. Newly investigated sites are denoted in bold.

Site	European	East African	East Asian	West African	Khoe-San	South Asian
Askham	0	0.052	0	0	0.947	0
Colesberg	0	0	0	0.277	0.722	0
**Genadendal**	0.154	0.077	0	0.077	0.615	0.077
**Graaff-Reinet**	0	0	0.061	0.091	0.758	0.091
**Greyton**	0.091	0.182	0	0.182	0.545	0
Heidelberg	0	0	0	0	0.9	0.1
Melkhoutfontein	0	0	0	0.1	0.8	0.1
**Nieu-Bethesda**	0	0	0	0.211	0.789	0
Railton	0	0	0	0.375	0.5	0.125
Riversdale	0	0	0.083	0.083	0.75	0.083
RotterdamFarm	0	0.143	0	0.143	0.571	0.143
Slangriver	0	0	0	0.125	0.625	0.25
Stormsvlei	0	0	0	0.4	0.6	0
Suurbraak	0	0.091	0.091	0.182	0.545	0.091
Swellendam	0.182	0	0	0.091	0.636	0.091
Wellington	0.1	0	0.05	0.35	0.45	0.05

Table S4: Y chromosome ancestries at the 22 sites. Newly investigated sites are denoted in bold.

Site	European	East African	East Asian	West-African	Khoe-San	South Asian
Askham	0.166	0	0	0.5	0.333	0
Colesberg	0.2	0	0	0.6	0.2	0
DistrictSix	0.375	0	0	0.5	0	0.125
EasternCape	0.285	0	0.142	0.428	0.142	0
**Genadendal**	0.666	0	0.111	0	0	0.222
**Graaff-Reinet**	0.181	0	0.045	0.727	0	0.045
**Greyton**	1	0	0	0	0	0
Heidelberg	0	0	0	0.666	0.333	0
**Kranshoek**	0	0	0.333	0.666	0	0
Melkhoutfontein	0.25	0	0	0.75	0	0
**Nieu-Bethesda**	0.25	0	0	0.375	0.375	0
NorthernCape	0.333	0	0.166	0.166	0	0.333
**Oudtshoorn**	0.444	0	0.111	0.444	0	0
**Prince Albert**	0.111	0	0	0.777	0.111	0
Railton	0.5	0	0	0	0.5	0
Riversdale	0.6	0	0	0.2	0	0.2
RotterdamFarm	0.4	0	0.2	0.2	0	0.2
Slangriver	0.5	0	0	0.5	0	0
Stormsvlei	0	0	0	0.5	0	0.5
Suurbraak	0	0	1	0	0	0
Swellendam	0.5	0	0.125	0.375	0	0
Wellington	0	0	0	0.333	0.333	0.333

Table S5: Number of individuals used at each site to make inferences about mitochondrial, autosomal and Y chromosomal ancestries.

Site	Mitochondria	Autosomal	Y chromosome
Askham	18	19	12
Colesberg	18	20	5
District Six	0	8	8
Eastern Cape	0	6	6
Genadendal	13	26	9
Graaff-Reinet	33	35	22
Greyton	11	13	7
Heidelberg	10	28	3
Kranshoek	0	10	3
Melkhoutfontein	10	15	4
Nieu-Bethesda	19	17	8
Northern Cape	0	11	6
Oudtshoorn	0	14	9
Prince Albert	0	10	9
Railton	8	15	2
Riversdale	12	24	5
RotterdamFarm	7	8	5
Slangriver	8	14	2
Stormsvlei	10	9	2
Suurbraak	11	24	1
Swellendam	11	10	8
Wellington	20	20	3
All 22 sites	219	356	139

Table S6: List of populations included in the dataset.

Population	Latitude	Longitude	Published in	Language	Subsistence	Geographical area
Amhara	10	39	[50]	Afro-Asiatic	Agriculturalist	EastAfrica
Baniamer	19.15	35.66	[52]	Afro-Asiatic	Agriculturalist	EastAfrica
Banyarwanda	2.16	33.69	[50]	Niger-Kordofanian	Agriculturalist	EastAfrica
Bitonga	-24.25	34.78	[48]	Niger-Kordofanian	Agriculturalist	SouthernAfrica
CEU_EUR	49.89	5.05	[49]	Indo-European	Agriculturalist	Europe
CHB_EAS	39.88	116.41	[49]	NA	NA	EastAsia
ColouredColesberg	-30.7	25.08	[1]	Indo-European	Agriculturalist	SouthernAfrica
ColouredWellington	-33.64	19	[1]	Indo-European	Agriculturalist	SouthernAfrica
Coloured_Askham	-26.98	20.78	[1]	Indo-European	Agriculturalist	SouthernAfrica
GBR_EUR	51.51	-0.13	[49]	Indo-European	Agriculturalist	Europe
GIH_SAS	22.75	73.09	[49]	Indo-European	Agriculturalist	SoutheastAsia
GuiGhanaKgal	-23.65	24.67	[1]	Khoisan	Hunter-Gatherer	Khoisan
Hadandawa	20.8	35.53	[52]	Afro-Asiatic	Agriculturalist	EastAfrica
Hadza	-3.11	33.25	[52]	Khoisan	Agriculturalist	EastAfrica
Hamer	4.84	36.52	[52]	Afro-Asiatic	Agriculturalist	EastAfrica
Heidelberg	-34.08	20.96	[13]	Indo-European	NA	SouthernAfrica
Ju/'hoansi	-23.54	24.18	[1]	Khoisan	Hunter-Gatherer	SouthernAfrica
Karretjie	-30.71	25.1	[1]	Khoisan	Agriculturalist	SouthernAfrica
Khomani	-26.94	20.66	[1]	Khoisan	Agriculturalist	SouthernAfrica
KHV_EAS	10.51	106.67	[49]	NA	NA	EastAsia
Khwe	-18.2	22.16	[1]	Khoisan	Hunter-Gatherer	SouthernAfrica
Kikuyu	-2.06	37.68	[50]	Niger-Kordofanian	Agriculturalist	EastAfrica
LWK_AFR	0.6	34.57	[49]	Niger-Kordofanian	Agriculturalist	EastAfrica
Mandinka	13.32	-16.16	[50]	Niger-Kordofanian	Agriculturalist	WestAfrica
Melkhoutfontein	-34.33	21.42	[13]	Indo-European	NA	SouthernAfrica
Oromo	9.25	36.89	[50]	Afro-Asiatic	Agriculturalist	EastAfrica
Pedi	-23.84	29.98	[19]	Niger-Kordofanian	Agriculturalist	SouthernAfrica
Railton	-34.03	20.43	[13]	Indo-European	NA	SouthernAfrica
Riversdale	-34.09	21.26	[13]	Indo-European	NA	SouthernAfrica
RotterdamFarm	-34.06	20.41	[13]	Indo-European	NA	SouthernAfrica
Sabue	7.1	35.55	[52]	Nilo-Saharan	Agriculturalist	EastAfrica
Sandawe	-7.16	35.42	[52]	Khoisan	Agriculturalist	EastAfrica
Slangriver	-34.08	20.94	[13]	Indo-European	NA	SouthernAfrica
Sotho	-29.35	25.38	[50]	Niger-Kordofanian	Agriculturalist	SouthernAfrica
Stormsvlei	-34.09	20.09	[13]	Indo-European	NA	SouthernAfrica
STU_SAS	7.2	80.86	[49]	NA	NA	SoutheastAsia
Suurbraak	-34.01	20.65	[13]	Indo-European	NA	SouthernAfrica
Swellendam	-34.02	20.45	[13]	Indo-European	NA	SouthernAfrica
Wolof	15.8	-16.52	[50]	Niger-Kordofanian	Agriculturalist	WestAfrica
Xade	-22.34	23.01	[46]	Khoisan	Hunter-Gatherer	SouthernAfrica
Xhosa	-32.72	26.88	[19]	Niger-Kordofanian	Agriculturalist	SouthernAfrica
Xun	-14.63	17.67	[1]	Khoisan	Hunter-Gatherer	SouthernAfrica
YRI_AFR	8.01	3.98	[49]	Niger-Kordofanian	Agriculturalist	WestAfrica
Zulu	-30.4	29.5	[50]	Niger-Kordofanian	Agriculturalist	SouthernAfrica
Afrikaner	-28.82	24.99	[21]	Indo-European	Agriculturalist	SouthernAfrica
Nama	-29	17.1	[51]	Khoisan	Pastoralist	SouthernAfrica
NAMA	-22.7	17.11	[1]	Khoisan	Pastoralist	SouthernAfrica
Temoro	-22.03	47.91	[45]	NA	Agriculturalist	SouthernAfrica
Vezo	-23.54	43.75	[45]	NA	Pastoralist	SouthernAfrica
Mikea	-21.65	43.86	[45]	NA	Hunter-Gatherer	SouthernAfrica
Coloured_NorthernCape	-29.91	20.12	[28]	Indo-European	NA	SouthernAfrica
Coloured_EasternCape	-32.04	26.86	[28]	Indo-European	NA	SouthernAfrica
Coloured_DistrictSix	-33.93	18.43	[28]	Indo-European	NA	SouthernAfrica
Nieu-Bethesda	-31.87	24.55	This study	Indo-European	NA	SouthernAfrica
Oudtshoorn	-33.59	22.2	This study	Indo-European	NA	SouthernAfrica
Prince Albert	-33.22	22.03	This study	Indo-European	NA	SouthernAfrica
Genadendal	-34.03	19.56	This study	Indo-European	NA	SouthernAfrica
Graaff-Reinet	-32.26	24.54	This study	Indo-European	NA	SouthernAfrica
Greyton	-34.05	19.61	This study	Indo-European	NA	SouthernAfrica
Kranshoek	-34.09	23.3	This study	Indo-European	NA	SouthernAfrica

Table S7: Expected r-squared values for the local ancestry inference from MOSAIC for each of the 22 sites.

Site	Expected r-squared
Coloured_Askham	0.7332515
ColouredColesberg	0.6987475
Coloured_DistrictSix	0.8595979
Coloured_EasternCape	0.7743049
Coloured_NorthernCape	0.8319147
ColouredWellington	0.7729508
Genadendal	0.7511367
Graaff-Reinet	0.6416341
Greyton	0.7612225
Heidelberg	0.6598167
Kranshoek	0.8508349
Melkhoutfontein	0.7026651
Nieu-Bethesda	0.6554671
Oudtshoorn	0.7353771
Prince Albert	0.6410863
Railton	0.6908134
Riversdale	0.6848675
RotterdamFarm	0.6781834
Slangriver	0.6842144
Suurbraak	0.6961573
Stormsvlei	0.6438075
Swellendam	0.7664059

Table S8: Mitochondrial DNA haplogroup assignment by ancestry.

Haplogroup	Most prevalent ancestry	Reference
B4a	East Asian	[27]
B4b	East Asian	[27]
B5b	East Asian	[68]
E1a	South Asian	[69]
H	European	[27]
H1a	European	[27]
H1c	European	[27]
J1c	European	[70]
L0a	Bantu-speaker	[6]
L0d	Khoe-San	[71];[72];
L0f	East African	[73]
L1b	Bantu-speaker	[74]
L1c	Bantu-speaker	[6]
L2a	Bantu-speaker	[6]
L3	Bantu-speaker	[75]
L3d	Bantu-speaker	[76]
L3e	Bantu-speaker	[6]
L4b	East African	[77]
L5a	East African	[77]
M1a	European	[78]
M6a	South Asian	[75]
M18	South Asian	[79]
M2a	South Asian	[79]
M2b	South Asian	[79]
M33	South Asian	[79]
M42	South Asian	[80]
M5a	South Asian	[79]
U2a	South Asian	[81]
U7a	South Asian	[81]

Table S9: Y-chromosome haplogroup assignment by ancestry.

Y-chr haplogroup	Most prevalent ancestry	Reference
A1b	Khoe-San	[82]
A0-T	Khoe-San	[83]
B2a	Bantu-speaker and West African	[84]
B2b	Bantu-speaker and West African	[82]
C	East Asian	[85]
E1b	Bantu-speaker and West African	[82]
E2b	Bantu-speaker and West African	[82]
E2	Bantu-speaker and West African	[86]
G2a	European	[75]
G2b	West Asian	[87]
H	South Asian	[75]
I1	European	[75]
I1a	European	[75]
I2	European	[75]
I2a	European	[75]
J	European	[75]
J2a	European	[75]
J2b	European	[75]
L1a	South Asian	[88]
N1c	East Asian	[75]
O1a	East Asian	[75]
R1a	European	[75]
R1b	European	[89]
R2	South Asian	[75]
R2a	South Asian	[75]
T	European	[90]
