# Supplementary material for: Wide-scale geographical analysis of genetic ancestry in the South African Coloured population
Source: BMC Biol. 2025 Jul 22;23:219. doi: 10.1186/s12915-025-02317-5 (PMC12281806; doi:10.1186/s12915-025-02317-5)
Supplement: Supplementary file 6 — Additional file 6. PONG visualization of unsupervised ADMIXTURE analysis with 50 iterations for the full dataset, for ancestries K = 2 to K = 12. The best identified K through cross validation was K = 10 (Additional File 7: Fig. S7). [file 12915_2025_2317_MOESM6_ESM.pdf]

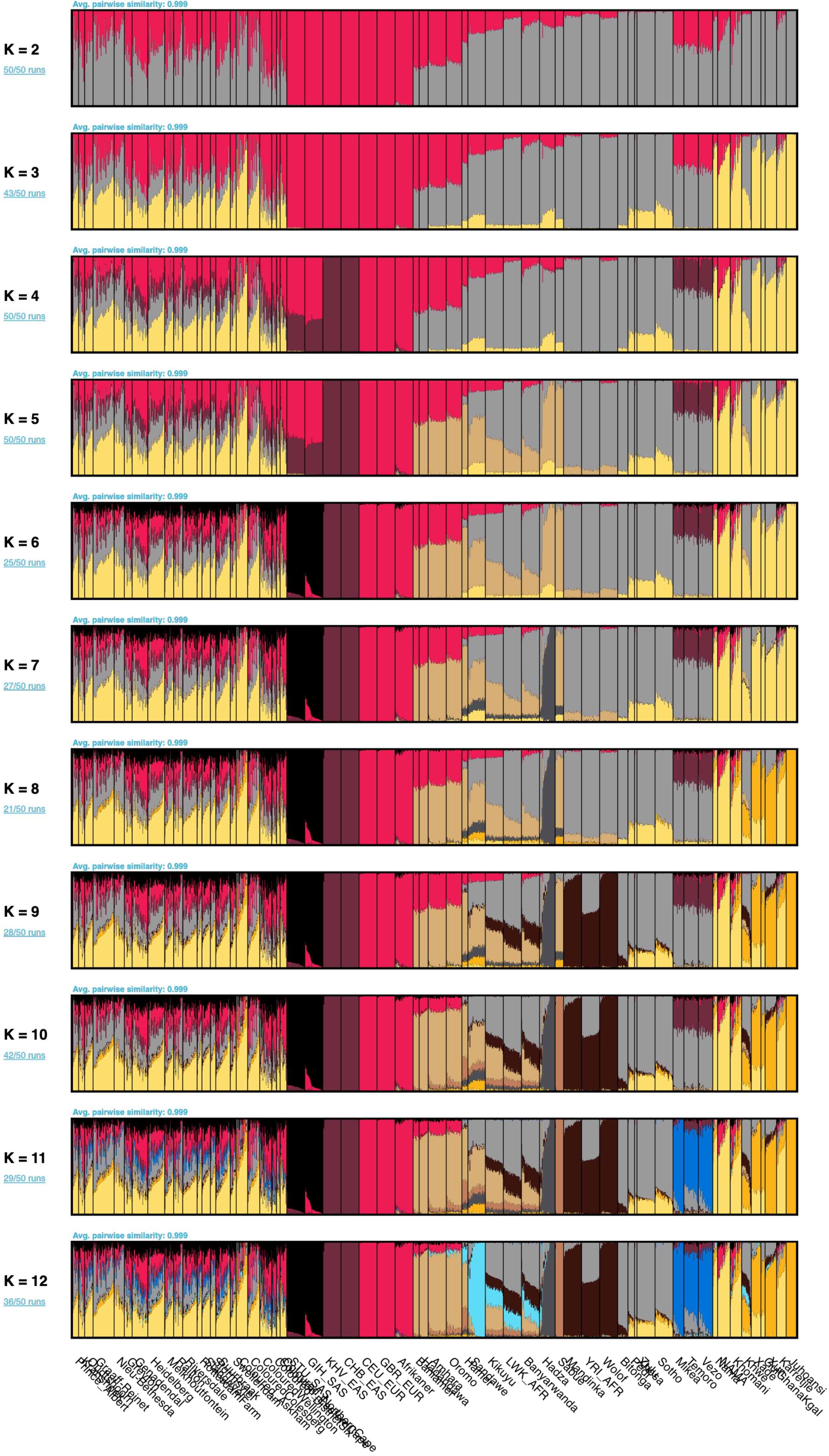
Avg. pairwise similarity: 0.999
K = 2
50/50 runs
K = 3
43/50 runs
K = 4
50/50 runs
K = 5
50/50 runs
K = 6
25/50 runs
K = 7
27/50 runs
K = 8
21/50 runs
K = 9
28/50 runs
K = 10
42/50 runs
K = 11
29/50 runs
K = 12
36/50 runs
Graaff-Reinet
Nieu-Bethesda
Genadendal
Heidelberg
Riversdale
GIH_SAS
KHV_EAS
CHB_EAS
CEU_EUR
GBR_EUR
Afrikaner
Amhara
Oromo
Sandawe
Kikuyu
LWK_AFR
Banyarwanda
Hadza
Mandinka
YRI_AFR
Wolof
Bitonga
Sotho
Mikea
Temoro
Vezo
Khomani
GhanaKgal
Juhoansi
